# Usefulness of the C_2_HEST Score in Predicting the Clinical Outcomes of COVID-19 in Diabetic and Non-Diabetic Cohorts

**DOI:** 10.3390/jcm11030873

**Published:** 2022-02-07

**Authors:** Damian Gajecki, Adrian Doroszko, Małgorzata Trocha, Katarzyna Giniewicz, Krzysztof Kujawa, Marek Skarupski, Jakub Gawryś, Tomasz Matys, Ewa Szahidewicz-Krupska, Piotr Rola, Barbara Stachowska, Jowita Halupczok-Żyła, Barbara Adamik, Krzysztof Kaliszewski, Katarzyna Kilis-Pstrusinska, Krzysztof Letachowicz, Agnieszka Matera-Witkiewicz, Michał Pomorski, Marcin Protasiewicz, Marcin Madziarski, Klaudia Konikowska, Agata Remiorz, Maja Orłowska, Krzysztof Proc, Małgorzata Szymala-Pedzik, Joanna Zorawska, Karolina Lindner, Janusz Sokołowski, Ewa A. Jankowska, Katarzyna Madziarska

**Affiliations:** 1 Clinical Department of Internal Medicine, Hypertension and Clinical Oncology, Faculty of Medicine, Wroclaw Medical University, Borowska 213, 50-556 Wroclaw, Poland; damian.gajecki@umw.edu.pl (D.G.); jakub.gawrys@umw.edu.pl (J.G.); tomasz.matys@umw.edu.pl (T.M.); ewa.szahidewicz-krupska@umw.edu.pl (E.S.-K.); 2 Department of Pharmacology, Faculty of Medicine, Wroclaw Medical University, Mikulicz-Radecki Street 2, 50-345 Wroclaw, Poland; malgorzata.trocha@umw.edu.pl; 3 Statistical Analysis Centre, Wroclaw Medical University, K. Marcinkowski Street 2-6, 50-368 Wroclaw, Poland; katarzyna.giniewicz@umw.edu.pl (K.G.); krzysztof.kujawa@umw.edu.pl (K.K.); 4 Faculty of Pure and Applied Mathematics, Wroclaw University of Science and Technology, Wybrzeże Wyspiańskiego Street 27, 50-370 Wroclaw, Poland; marek.skarupski@pwr.edu.pl; 5 Department of Cardiology, Provincial Specialized Hospital, Iwaszkiewicza 5 Street, 59-220 Legnica, Poland; piotr.rola@gmail.com; 6 Department of Endocrinology, Diabetes and Isotope Therapy, Faculty of Medicine, Wroclaw Medical University, Ludwik Pasteur 4, 50-367 Wroclaw, Poland; barbara.stachowska@umw.edu.pl (B.S.); jowitahz@gmail.com (J.H.-Ż.); 7 Clinical Department of Anaesthesiology and Intensive Therapy, Faculty of Medicine, Wroclaw Medical University, Borowska Street 213, 50-556 Wroclaw, Poland; barbara.adamik@umw.edu.pl; 8 Department of General, Minimally Invasive and Endocrine Surgery, Faculty of Medicine, Wroclaw Medical University, Borowska Street 213, 50-556 Wroclaw, Poland; krzysztof.kaliszewski@umw.edu.pl; 9 Clinical Department of Paediatric Nephrology, Faculty of Medicine, Wroclaw Medical University, Borowska Street 213, 50-556 Wroclaw, Poland; katarzyna.kilis-pstrusinska@umw.edu.pl; 10 Clinical Department of Nephrology and Transplantation Medicine, Faculty of Medicine, Wroclaw Medical University, Borowska Street 213, 50-556 Wroclaw, Poland; krzysztof.letachowicz@umw.edu.pl (K.L.); or katarzyna.madziarska@umw.edu.pl (K.M.); 11 Laboratory of Biological Activity Tests and Collection of Biological Material, Wroclaw Medical University, Borowska Street 211A, 50-556 Wroclaw, Poland; agnieszka.matera-witkiewicz@umw.edu.pl; 12 Clinical Department of Gynecology and Obstetrics, Faculty of Medicine, Wroclaw Medical University, Borowska Street 213, 50-556 Wroclaw, Poland; michal.pomorski@umw.edu.pl; 13 Clinical Department and Clinic of Cardiology, Wroclaw Medical University, Borowska Street 213, 50-556 Wroclaw, Poland; marcin.protasiewicz@umw.edu.pl; 14 Clinical Department of Rheumatology and Internal Medicine, Faculty of Medicine, Wroclaw Medical University, Borowska Street 213, 50-556 Wroclaw, Poland; madziarski.marcin@gmail.com; 15 Department of Dietetics, Wroclaw Medical University, Parkowa Street 34, 51-616 Wroclaw, Poland; klaudia.konikowska@umw.edu.pl; 16 Clinical Department of Nephrology and Transplantation Medicine, Wroclaw University Hospital, Borowska Street 213, 50-556 Wroclaw, Poland; agata.remiorz@gmail.com; 17 Department of Emergency Medicine, Wroclaw University Hospital, Borowska Street 213, 50-556 Wroclaw, Poland; majaorlowska@gmail.com; 18 Clinical Department of Rheumatology and Internal Medicine, Wroclaw University Hospital, Borowska Street 213, 50-556 Wroclaw, Poland; kproc@usk.wroc.pl; 19 Clinical Department of Geriatrics, Wroclaw Medical University, Pasteur 4 Street, 50-367 Wroclaw, Poland; Malgorzata.szymala-pedzik@umw.edu.pl (M.S.-P.); Joanna.zorawska@umed.wroc.pl (J.Z.); karolina.lindner@umw.edu.pl (K.L.); 20 Department of Emergency Medicine, Faculty of Medicine, Wroclaw Medical University, Borowska Street 213, 50-556 Wroclaw, Poland; janusz.sokolowski@umw.edu.pl; 21 Institute of Heart Diseases, Wroclaw Medical University, Borowska Street 213, 50-556 Wroclaw, Poland; ewa.jankowska@umw.edu.pl; 22 Institute of Heart Diseases, University Hospital in Wroclaw, Borowska Street 213, 50-556 Wroclaw, Poland

**Keywords:** COVID-19, SARS-CoV-2, diabetes mellitus, outcomes, C_2_HEST score, mortality, prediction

## Abstract

Background: Diabetes mellitus is among the most frequent comorbidities worsening COVID-19 outcome. Nevertheless, there are no data regarding the optimal risk stratification of patients with diabetes and COVID-19. Since individual C_2_HEST components reflect the comorbidities, we assumed that the score could predict COVID-19 outcomes. Material and Methods: A total of 2184 medical records of patients hospitalized for COVID-19 at the medical university center were analyzed, including 473 diabetic patients and 1666 patients without any glucose or metabolic abnormalities. The variables of patients’ baseline characteristics were retrieved to calculate the C_2_HEST score and subsequently the diabetic and non-diabetic subjects were assigned to the following categories: low-, medium- or high-risk. The measured outcomes included: in-hospital mortality; 3-month and 6-month all-cause mortality; non-fatal end of hospitalization (discharged home/sudden-deterioration/rehabilitation) and adverse in-hospital clinical events. Results: A total of 194 deaths (41%) were reported in the diabetic cohort, including 115 in-hospital deaths (24.3%). The 3-month and 6-month in-hospital mortality was highest in the high-risk C_2_HEST stratum. The C_2_HEST score revealed to be more sensitive in non-diabetic-group. The estimated six-month survival probability for high-risk subjects reached 0.4 in both cohorts whereas for the low-risk group, the six-month survival probability was 0.7 in the diabetic vs. 0.85 in the non-diabetic group—levels which were maintained during whole observation period. In both cohorts, receiver operating characteristics revealed that C_2_HEST predicts the following: cardiogenic shock; acute heart failure; myocardial injury; and in-hospital acute kidney injury. Conclusions: We demonstrated the usefulness and performance of the C_2_HEST score in predicting the adverse COVID-19 outcomes in hospitalized diabetic subjects.

## 1. Introduction

Since the outbreak of coronavirus disease 2019 (COVID-19) in China in December 2019, considerable attention has been focused on its elucidation. The natural history and outcome of COVID-19 patients initially hospitalized in a medical ward remain unpredictable. Since the risk score systems have proven useful in clinical decision-making, there is an urgent need to help physicians effectively triage COVID-19 subjects. Numerous patients rapidly deteriorate after a period of relatively mild symptoms, emphasizing the need for early risk stratification. A few studies have aimed to develop predictive or risk score models in order to facilitate clinical decision making associated with COVID-19. Nevertheless, risk scores, such as SOFA and MEWS, lack sufficient sensitivity and specificity to predict mortality when applied to the COVID-19 cohort [1,2]. As a result, prognostic factors of COVID-19 patients among the European population are missing. Moreover, it remains unclear whether these models could be applied to the cohorts with specific comorbidities which are already known to worsen the disease course and affecting the outcome. 

Diabetes mellitus is among the most frequent comorbidities in patients with COVID-19. Individuals with diabetes have been identified as having worse outcomes when infected by SARS-CoV-2. In diabetes, an imbalance between coagulation and fibrinolysis occurs, with increased levels of coagulation factors and the relative inhibition of the fibrinolytic system. Both insulin resistance and diabetes are associated with endothelial dysfunction, and enhanced platelet aggregation and activation [3]. These abnormalities favor the development of a hypercoagulable pro-thrombotic state. Nevertheless, as far as the literature is concerned, there are no data regarding the most appropriate risk stratification and management of patients with diabetes and COVID-19 [4]. The European Society of Cardiology (ESC) recognized hypertension, diabetes and severe obesity as concomitant conditions that may be associated with a more severe course of COVID-19 in its recent guidance for the diagnosis and management of cardiovascular disease during the COVID-19 pandemic. Of note, hypertension, obesity, and CVD are frequent comorbidities in patients with diabetes [5]. Therefore, special attention is required to stratify the risk appropriately, particularly since the disease may be associated with an increased severity of symptoms and complications [6]. 

The C_2_HEST score was initially implemented to predict the risk for atrial fibrillation and it is based on the simple comorbidities’ calculation. Since individual C_2_HEST components reflect the comorbidities, we assumed that the score could predict unfavorable clinical COVID-19 outcomes in subjects with concomitant diabetes mellitus. Liang et al. were the first to show that the count of comorbidities predicted critical illness in hospitalized subjects [7], which prompted us to investigate the predictive value of the C_2_HEST score in the COVID-19 cohort.

Hence, in this study, the analysis of hospitalized COVID-19 patients with and without diabetes mellitus was performed to verify the prognostic efficacy of the C_2_HEST score in predicting the outcomes, including death as well as non-fatal clinical events in the course of hospitalization in these subpopulations.

## 2. Materials and Methods

### 2.1. Study Design and Participants

We retrospectively analyzed the medical records of patients hospitalized for COVID-19 at the medical university center between February 2020 and June 2021. The protocol for the COLOS retrospective study was approved by the Institutional Review Board and Ethics Committee of Wroclaw Medical University, Wroclaw, Poland (No: KB-444/2021). The routine data were retrospectively collected; therefore, written informed consent to participate in this study was not required. The Bioethics Committee approved the publication of fully anonymized data.

All patients were admitted to the hospital because of COVID-19 symptoms and a positive test result for SARS-CoV-2. The testing was strictly based on the protocol published by the World Health Organization (WHO). Nasopharyngeal swab specimens were obtained from all patients and SARS-CoV-2 RNA was detected in the samples by reverse-transcription polymerase chain reaction (RT-PCR), strictly according to the manufacturer’s instructions. Diabetes mellitus was confirmed according to the American and Polish Diabetes Associations’ criteria.

The analyzed data included: demographic information; clinical characteristics; breathing support; smoking; comorbidities; home medication; laboratory results—and the course of hospitalization including applied treatment and adverse clinical events: shock; pulmonary embolism; deep vein thrombosis; myocardial infarction; myocardial injury; acute heart failure; stroke/TIA; pneumonia; complete respiratory failure; systemic inflammatory response syndrome SIRS; sepsis; acute kidney injury; acute liver; dysfunction; multiple organ dysfunction syndrome (MODS); and bleeding.

### 2.2. Follow Up and Outcomes

The follow up period started from the day of admission to the hospital and ended on the day of discharge or death. The entire hospitalization period was analyzed. Further information regarding the patients’ deaths was collected after 90 and 180 days from admission. Patient characteristics were obtained from individual clinical records. 

The measured outcomes included: in-hospital mortality; 3-month and 6-month all-cause mortality; and the end of hospitalization not due to death (discharged home/emergency transfer to another center or deterioration/transferred for rehabilitation). Secondary outcomes included: the need for mechanical ventilation support; myocardial injury; shock; acute heart failure; pulmonary embolism; stroke; acute kidney injury; acute liver dysfunction; pneumonia; sepsis; systemic inflammatory response syndrome (SIRS); multiple organ dysfunction syndrome (MODS); and bleeding.

### 2.3. C_2_HEST Score Stratification

In this study, we included 473 patients with diabetes mellitus and 1666 non-diabetic patients who acted as the control group. The patients’ characteristics at baseline were retrieved from the dataset in order to calculate the C_2_HEST score with a total of 6 individual components including coronary artery disease (1 point); chronic obstructive pulmonary disease (COPD, 1 point each); hypertension (1 point); elderly (age  ≥  75 years, 2 points); systolic heart failure (HF, 2 points); and thyroid disease (1 point). Of note, the coronary artery disease criterion was met with a positive history of myocardial infarction or coronary revascularization (MI, as 1 point). Moreover, in subsequent sensitivity analyses, the “thyroid disease” was replaced more precisely with “hyperthyroidism” and “hypothyroidism”. These risk factors were determined based on a combination of medical record review and interview at baseline visits. Afterwards, the subjects were assigned to one of the three primary risk categories:

- 0–1—low;
- 2–3—medium;
- ≥4—high.

### 2.4. Statistical Analysis

Descriptive data are presented as numbers and percentages for categorical variables, and as the mean with a standard deviation range (minimum–maximum) and number of non-missing values for numerical variables. As an omnibus test, a chi-square test was used for categorical variables with more than 5 expected cases in each group, whereas a Fisher exact test was used for cases with fewer cell counts. Welch’s ANOVA was performed for continuous variables due to unequal variances between risk-strata and a sample size large enough for appropriateness of asymptotic results. Post hoc analysis for continuous variables was performed using a Games–Howell test with Tukey correction. For categorical variables, a post hoc test was the same as the omnibus test but performed in subgroups with Bonferroni correction.

In-hospital mortality and all-cause mortality was available as right-censored data, so time-dependent ROC analysis with an inverse probability of censoring weighting (IPCW) estimation was performed for those variables. The C_2_HEST score was assessed through the time-dependent area under the curve (AUC). A log-rank test was used to confirm the differences in survival curves between risk strata. Proportional hazard assumption was verified using the Grambsch–Therneau test. A Cox proportional hazard model was used to analyze the hazard ratio (HR) for the C_2_HEST score, its components, and risk strata.

For the secondary outcomes, due to their dichotomic nature, a logistic regression model was fitted. Classical ROC analysis was performed, and an AUC measure was used for assessing predictive capabilities. An odds ratio (OR) was reported as an effect size for the influence of the C_2_HEST score, its components and risk strata. 

All statistical analyses were performed with R version 4.0.4 using the packages time-ROC, pROC [8], survival [9], Coin [10], and odds ratio [11]. A significance level of 0.05 was selected for all statistical analyses.

## 3. Results

### 3.1. Baseline Characteristics of the Studied Population and Comorbidities

The baseline characteristics of this study and control group are summarized in Table 1 and in Supplementary Table S1. In both groups, a higher C_2_HEST score was associated with a higher average age and number of comorbidities. Moreover, the prevalence of cigarette smoking, moderate/severe valvular heart disease, previous TIA/stroke and chronic kidney disease was significantly greater in the high- than in low-risk C_2_HEST stratum. The prevalence of asthma did not differ between groups (Table 1 and Supplementary Table S1). 

Among the patient-reported symptoms, vital signs, and abnormalities measured during the baseline physical examination of the non-diabetic group, the subjects from the high-risk compared to low-risk stratum were characterized by a higher prevalence of dyspnea and diarrhea, a decreased occurrence of smell dysfunction, as well as a lower baseline heart rate and saturation. In the physical examination of non-diabetic patients, the frequency of crackles, pulmonary congestion, and hemiplegia were significantly higher with the increase in the C_2_HEST stratum. Interestingly, the diabetic cohort was more homogeneous and such differences in the vital signs and symptoms at admission to the hospital were not found. However, patients from both groups, with a higher C_2_HEST score, presented a higher pulse pressure and more frequently demonstrated wheezing and peripheral edema. Interestingly, no significant differences regarding cough, chest pain, taste dysfunction, and body temperature were found between the strata of the diabetic and non-diabetic groups (Table 2).

### 3.2. Characteristics of the In-Hospital Laboratory Tests and Treatment Applied

#### 3.2.1. Laboratory Assays

The detailed characteristics of the laboratory parameters measured during hospitalization for the diabetic and non-diabetic cohorts are presented in the Supplementary Table S2. 

There were no differences between the C_2_HEST strata in both cohorts regarding white blood cells upon admission; however, upon discharge, the high-risk subjects were more commonly characterized with lymphopenia and a low platelet count. The lower hemoglobin level and higher INR in the high-risk C_2_HEST stratum were found in both groups during the whole observation period. Interestingly, no significant differences between the strata were noted in terms of acid-base balance parameters nor in terms of the procalcitonin, IL-6, ESR and D-dimer levels, at baseline or at the time of discharge. The CRP level was similar in different strata at the time of admission. It was significantly lower in the low-risk stratum in the non-diabetic cohort. Such a difference was not seen in the diabetic group. 

In the diabetic group, contrary to the non-diabetic one, the higher C_2_HEST score was related to more frequent respiratory failure at discharge. The parameters of kidney function, including urea, creatinine, eGFR were maintained as significantly worse in the high-risk C_2_HEST stratum during the whole hospitalization period in both groups. No significant differences between groups regarding Fe, TIBC, sTfR, vitamin B12, and folic acid levels both upon admission and upon discharge were observed. Nevertheless, the baseline ferritin level was lower in the high-risk stratum of the diabetic cohort at admission. In the diabetic cohort, no differences were noted between strata in terms of the glucose level during the whole observation; however, the high-risk group had a significantly lower HbA1c concentration. 

The markers of cardiac injury evaluated in both cohorts, including troponin T and NT-proBNP levels, were greater in the high-risk stratum during the whole observation period. Furthermore, acute myocardial injury, as assessed by the increase in the troponin level of >5-fold above the upper-range limit, was more common in this group. No significant differences regarding the cortisol and TSH between the groups were noted. No differences regarding ALT, bilirubin, and GGTP upon admission were present. 

#### 3.2.2. Specific Treatment Applied during the Hospitalization Period

In terms of management, there were no differences regarding the use of systemic corticoids, remdesivir, tocilizumab nor convalescent plasma between the C_2_HEST risk strata. Only specific antimicrobial treatment was more commonly applied in the subjects from the high-risk C_2_HEST stratum (Supplementary Table S3). 

#### 3.2.3. Supportive Treatment Applied during Hospitalization

The need for oxygen supplementation increased with C_2_HEST in the non-diabetic cohort, including via low-oxygen support and high flow nasal cannula, whereas the oxygenation parameters during the period of qualification for advanced respiratory support decreased. 

The need for urgent coronary angiography and revascularization also increased with the C_2_HEST score in non-diabetic patients. Interestingly, no significant differences between groups in terms of the need for the use of catecholamines or *de novo* hemodialysis were observed in either cohort. (Table 3). 

### 3.3. Associations of the C_2_HEST Score with Fatal Outcomes

#### 3.3.1. C_2_HEST Score Results and Mortality

A total of 194 deaths (41%) were reported in the diabetic cohort (473 diabetic subjects), including 115 in-hospital deaths (24.3%). The in-hospital, 3-month and 6-month mortality was highest in the high-risk C_2_HEST stratum, reaching 34.7%, 53.4%, and 64.8%, respectively, and lowest in the low-risk stratum: 15.3%, 24.4%, and 37.2%, as appropriate. Similarly, in the non-diabetic cohort, a total of 376 deaths (22.6%) were reported. The in-hospital, 3-month, and 6-month mortality was also the highest in the high-risk C_2_HEST stratum—reaching 37.0%, 55.5%, and 68.0%, respectively (Table 4).

#### 3.3.2. Discriminatory Performance of the C_2_HEST Score on the Total All-Cause Mortality

**Figure 1:**
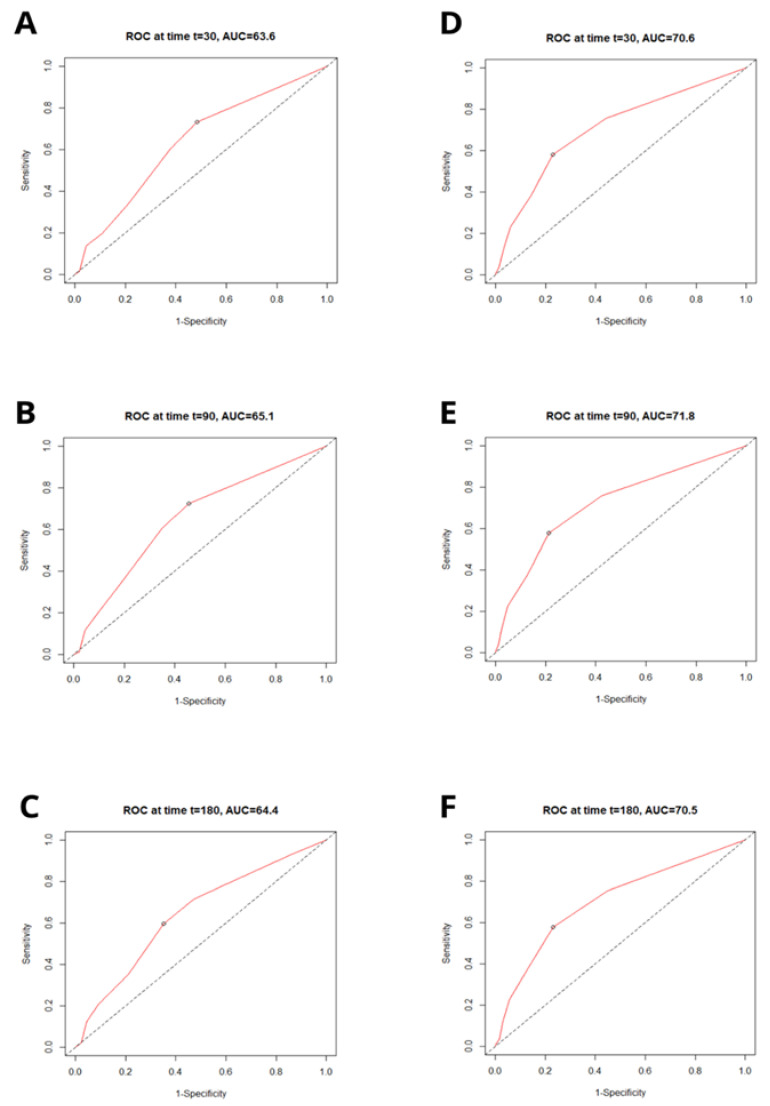
Receiver operating characteristic (ROC) curves for the C_2_HEST score in predicting total mortality in the diabetic (**A**–**C**) and non-diabetic (**D**–**F**) cohorts at 3 time points following the positive RT-PCR test (t = 30 days; t = 90 days; t = 180 days). AUC—area under curve.

The receiver operating characteristics (ROC) revealed that C_2_HEST moderately predicts the 1-, 3-, and 6-month mortality in diabetic cohort. Additionally, the C_2_HEST score revealed to be an even more sensitive tool in the non-diabetic group where it demonstrated a better predictive value. The C_2_HEST predicting the AUC in the diabetic vs. non-diabetic cohorts presents as follows: the 1-month AUC_30_ = 63.6% vs. 70.6%; 3-month AUC_90_ = 65.1% vs. 71.8%; and 6-month AUC_180_ = 64.4% vs. 70.5%. All the data were calculated for all-cause death without competing risk (Figure 1). 

**Figure 2:**
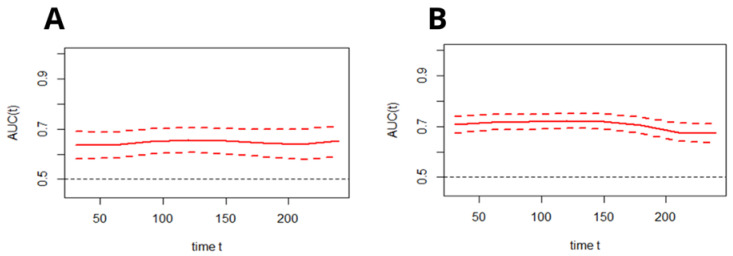
Time-dependent ROC analysis for the C_2_HEST predictive abilities of all-cause death in the diabetic (**A**) and non-diabetic (**B**) cohorts (mean with CI). AUC—area under curve.

Subsequently, the time–ROC analysis was performed in order to assess the predictive value of the C_2_HEST scale for all-cause mortality at a particular time from admission to the hospital in both cohorts. Figure 2 presents the time-dependent changes in the predictive value of the C_2_HEST score (changes of AUC in time, alongside with the CI).

#### 3.3.3. Discriminatory Performance of the C_2_HEST Score on the In-Hospital All-Cause Mortality–Time–ROC Analysis

**Figure 3:**
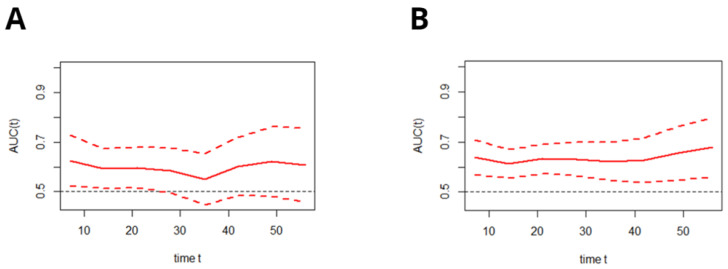
Time-dependent ROC analysis for the C_2_HEST predictive abilities of death during hospitalization in the diabetic (**A**) and non-diabetic (**B**) cohorts (mean with CI). AUC—area under curve.

As presented in Figure 3, the time-dependent AUC for the C_2_HEST score in predicting the in-hospital deaths in both cohorts remained moderate, regardless of the time of hospitalization.

#### 3.3.4. The Survival Probability for Hospitalized COVID-19 Patients

**Figure 4:**
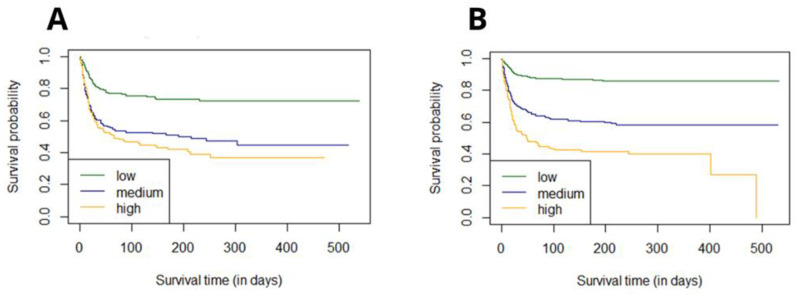
Analysis of the 6-month survival for the *low, medium,* and *high* C_2_HEST risk strata in the diabetic (**A**) and non-diabetic (**B**) cohorts.

The survival curves for groups were estimated using Kaplan–Meier functions, based on the original stratification-low/medium/high for 0–1/2–3/≥4 points, respectively. The *p* value for the Log-rank test was <0.0001 (Figure 4). The estimated six-month survival probability for high-risk subjects reached 0.4 in both cohorts whereas for the low-risk group, the six-month survival probability was 0.7 in the diabetic vs. 0.85 in the non-diabetic group—levels which were maintained during whole observation period. 

#### 3.3.5. Risk Strata Matching Analysis

In order to verify whether the original risk stratification included the low/medium/high-risk categories for 0–1/2–3/≥4 points, respectively, all the possible C_2_HEST intervals were analyzed, and for each one, the log-rank statistics test was performed (Supplementary Table S4). 

The highest value of the log-rank statistics for the diabetic cohort corresponded with the risk strata estimated as follows:

- 0–1—low;
- 2–5—medium;
- 6–8—high.

Such risk calculation results in better risk stratification than the generally accepted one, however, the subpopulation division classically used in the literature will be used in the rest of this study.

Nevertheless, the same statistical analysis for the non-diabetic cohort revealed that, for this subpopulation, the primary risk categories (0–1 = low; 2–3 = medium; >4 = high) best reflect the total mortality curves.

This analysis was also repeated for the in-hospital mortality (Supplementary Table S5). 

#### 3.3.6. Effect of the C_2_HEST Risk Stratification Result on COVID-19 Survival

Subsequently, two Cox models were analyzed to assess the effect of the C_2_HEST score stratification on COVID-19 mortality. The overall model takes an uncategorized value of the C_2_HEST score, and the assumption of proportional hazard was met. Increase in one point in the C_2_HEST score increased the total-death intensity in approximately 24.9% of the diabetic cohort (HR 1.249, 95% CI 1.163–1.341 *p* < 0.0001) and in 45.2% in the non-diabetic subjects (HR 1.452, 95% CI 1.382–1.525 *p* < 0.0001).

For diabetic patients, the change from the low to the medium category increased death likelihood by 2.34 times, whereas between the low- and high-risk group, the hazard ratio was 2.84. The results for the non-diabetic subjects reflects an increased death chance between the low- and medium-risk stratum by 3.51 and the low vs. high with 6.0 times. The results are presented in Table 5.

A Similar analysis was performed for in-hospital deaths. The overall model took into consideration an uncategorized value of the C_2_HEST score, and the assumption of proportional hazard was met. In the diabetic cohort, the one-point increase relates to a 19% increase in in-hospital death (HR 1.19, 95% CI 1.081–1.314, *p* < 0.0005). The risk of in-hospital death increases 2.16-fold and 2.11-fold between the medium- vs. low- and high- vs. low-risk strata, respectively (Table 6).

The analogous statistical model in the non-diabetic cohort revealed that the assumption of proportional hazard was met. However, for the group model, the *p* value was 0.033, so the null hypothesis was excluded, and the confidence intervals and *p* values were omitted. An increase in one point in the C_2_HEST score increased the in-hospital death intensity by 29%. The change from the low to the medium category increased the in-hospital death intensity by 2.1-fold, whereas between the low- and high-risk categories, the hazard ratio was 3.35. The results are presented in Table 6.

The associations of individual C_2_HEST score components in the C_2_HEST score with mortality are presented in Supplementary Table S10. As the results from the analysis of the Cox proportional hazard model (all-cause death) and competing risk regression model for other outcomes, among individual comorbidities, the highest prognostic value for in-hospital mortality had coronary artery disease and age in both cohorts.

### 3.4. Associations of the C_2_HEST Score with Other, Non-Fatal Outcomes 

In both cohorts, receiver operating characteristics (ROC) revealed that the C_2_HEST predicts: cardiogenic shock (AUC_diabetes_ = 0.725 vs. AUC_non-diabetes_ = 0.749) and acute heart failure (AUC_diabetes_ = 0.819 vs. AUC_non-diabetes_ = 0.868) well. The increase in one point in the C_2_HEST score increased the risk for cardiogenic shock by 53% in the diabetic vs. 63.6% in the non-diabetic group (OR_diabetes low vs. high_: 7.53 95% CI = 1.85–50.45 *p* = 0.012 OR_non-diabetes low vs. high_: 10.1 95% CI = 3.0–35.44, *p* < 0.0001). Similarly, myocardial injury (MI), as assessed by the >5-fold in-hospital increase in the troponin levels, as well as the acute heart failure, increased with the C_2_HEST score in both cohorts. For MI, the OR_diabetes overall_ = 1.17 (95% CI = 1.03–1.33, *p* = 0.014) and OR_non-diabetes overall_ = 1.44 (95% CI = 1.31–1.59, *p* < 0.0001), while the OR between strata for MI are: OR_diabetes low vs. high_: 2.16 (95% CI = 1.2–3.92, *p* = 0.01) and OR_non-diabetes low vs. high_: 5.59 (95% CI = 3.5–8.94, *p* < 0.0001). For acute heart failure (AHF), the OR_diabetes overall_ = 1.94 (95% CI = 1.59–2.43 *p* < 0.0001) and OR_non-diabetes overall_ = 2.07 (95% CI = 1.77–2.44, *p* < 0.0001) while the OR between the low and high groups for AHI are: OR_diabetes low vs. high_: 25.06 (95% CI = 7.22–158.17 *p* < 0.0001) and OR_non-diabetes low vs. high_: 36.68 (95% CI = 15.58–100.86, *p* < 0.0001). Table 7 and Supplementary Table S6). A similar tendency in both cohorts was observed for the occurrence of in-hospital acute kidney injury (AKI). Increase in one point in C_2_HEST increased the risk by 20.5% in the diabetic and by 29.9% in the non-diabetic group. The OR for the AKI was: OR_diabetes low vs. high_: 2.3 (95% CI = 1.3–4.1 *p* < 0.0042) and OR_non-diabetes low vs. high:_ 2.81 (95% CI = 1.7–4.51, *p* < 0.0001). Table 7 and Supplementary Table S6).

Interestingly, in both cohorts, there were no differences in the occurrence of stroke/TIA, complete respiratory failure, systemic inflammatory response syndrome (SIRS), nor multi-organ dysfunction syndrome (MODS). Additionally, an increase in the C_2_HEST score did not increase the prevalence of deep vein thrombosis and pulmonary embolism. 

It is worth noting that in the non-diabetic cohort, the increase in the C_2_HEST score was also related to the increased risk of: pneumonia OR_non-diabetes overall_ = 1.35 (95% CI = 1.26–1.45 *p* < 0.0001), sepsis OR_non-diabetes overall_ = 1.43 (95% CI = 1.1–1.84 *p* < 0.0054), acute liver dysfunction OR_non-diabetes overall_ = 1.3 (95% CI = 1.1–1.51 *p* < 0.008), and all types of bleeding OR_non-diabetes overall_ = 1.21 (95% CI = 1.06–1.37 *p* < 0.0038), with increased risk of upper-gastrointestinal tract bleeding for every C_2_HEST score: OR_non-diabetes overall_ = 1.49 (95% CI = 1.2–1.83 *p* < 0.002) and myocardial infarction OR_non-diabetes overall_ = 1.47 (95% CI = 1.11–1.91 *p* < 0.0042). Described differences had not been seen in the diabetic cohort; however, in this group, the increase in one point of the C_2_HEST scale coexists with a higher risk of complete respiratory failure OR_diabetes overall_ = 1.29 (95% CI = 1.02–1.69 *p* = 0.0377). 

All of the odds ratios for quantifying the strength of the association between the CH2EST score and the study endpoints and adverse events are demonstrated in Supplementary Table S6. The summarized discriminatory performance of the C_2_HEST score on the clinical events is presented in Supplementary Table S7.

### 3.5. Sensitivity Analysis

The results of the sensitivity analysis are summarized in Supplementary Tables S8a,b and S9a,b.

Interestingly, replacing the general definition of “thyroid disease” with the more precise term “hypothyroidism” and the cut-off point for age to a more liberal “>65 years” as scoring items in the C_2_HEST score resulted in a significant increase in the predictive value for the endpoints and most of the adverse clinical events in both cohorts. The all-cause mortality HR shows as follows: HR_diabetes overall_ = 1.21, (95% CI 1.08–1.34, *p* < 0.0006) HR_diabetes low vs. high_ = 2.5 (95% CI 1.01–6.17, *p* < 0.047—and for the non-diabetic cohort: HR_non-diabetes overall_ = 1.49, (95% CI 1.42–1.57, *p* < 0.0001), HR_non-diabetes low vs. high_ = 6.69 (95% CI 5.01–8.92, *p* < 0.0001). Interestingly, the predictive value for pulmonary embolism (PE) and venous thromboembolic disease (VTD) reached statistical significance, but still with a relatively low OR –PE OR_non-diabetes overall_ = 1.20 (95% CI 1.07–1.35 *p* = 0.0013) and OR _non-diabetes low vs. high_ = 2.17 (95% CI = 1.33–3.53, *p* = 0.0018). For VTD, OR_non-diabetes overall_ = 1.18 (95% CI 1.05–1.31 *p* = 0.0037) and OR_non-diabetes low vs. high_ = 1.98 (95% CI = 1.23–3.19, *p* = 0.0049). Additionally, the fact that statistical modification ends with changes in the end-point analysis in the non-diabetic cohort. In addition to conversion, the one point in the C_2_HEST score increases the risk of: all causes of shock by 23.3%; septic shock by 19.1%; and the cardiogenic shock the most—by 60.8%. Additionally, the modified C_2_HEST score had a better prognostic value for the TIA/stroke and complete respiratory failure which achieved statistical significance in non-diabetic subjects.

## 4. Discussion

To the best of our knowledge, this is the first study evaluating the usefulness of the C_2_HEST scale in predicting the outcome of a COVID-19 cohort with respect to the presence of diabetes mellitus. Since diabetes mellitus is considered to be an independent risk factor worsening COVID-19 course and mortality, its prediction abilities in this cohort required detailed analysis. The clinical manifestations of COVID-19 patients have shown that comorbidities including diabetes, hypertension, and atherosclerotic cardiovascular disease are very common and diabetes mellitus has emerged as a critical risk factor [12,13,14]. Previous studies have highlighted that those patients with cardiometabolic risk factors such as diabetes mellitus (DM) have been associated with worse clinical manifestation and higher mortality in COVID-19 [15,16]. Due to the heterogeneity of the diabetic cohort, including its metabolic compensation and the severity of the target organ damage, an individualized approach is pivotal for the early identification of patients being more susceptible to a poor COVID-19 outcome.

Hyperglycemia itself as well as chronically impaired glucose metabolism in the course of diabetes trigger numerous metabolic signaling pathways leading to chronic inflammatory disease and impaired immune response to infection [17], which include the uncontrolled secretion of INF-α, other pro-inflammatory cytokines, and chemokines [18]. According to the results from a recent study by Guo J. et al. [19], the mortality of COVID19 is associated with elevated serum levels of innate inflammatory cytokine IL-6 and inflammatory chemokines IL-8 and IP10 consistently across strata of diabetes. Consequently, the mortality rate of COVID-19 patients particularly associated with DM in the presence of some other comorbidities [20] is considerably greater than that observed in patients without diabetes due to the higher risk of developing cytokine storm and greater susceptibility to multiorgan failure, particularly in those previously affected by diabetes. Therefore, diabetic subjects are more likely to present with clinical symptoms and complications when compared to those without glucose metabolism abnormalities [21]. Our study revealed that, alongside a higher C_2_HEST stratum, the mortality significantly increases irrespectively in the presence of glucose metabolism abnormalities. The ROC analysis revealed that C_2_HEST moderately predicts the 1-, 3- and 6-month mortality in the diabetic cohort and its predictive value was even better in non-diabetic subjects. Noteworthily, 1.82-fold higher mortality was seen in the diabetic cohort, which is in accordance with the meta-analysis by Kumar et al., in which a two-fold increase in mortality associated with diabetes mellitus was demonstrated [22]. According to the results of the Kulcsar et al. model of the MERS-CoV infection in diabetic mice, diabetes mellitus is related to more severe systemic inflammation with a higher expression of inflammation mediators playing a pivotal role in the pathogenesis of the COVID-19 cytokine storm [23].

The increased vascular superoxide production with endothelial dysfunction, defective CD4+ and CD8+ T-cells and the impaired complement system in diabetic patients make this group more susceptible to poor outcomes in the course of COVID-19 [24,25,26,27].

The survival curves estimated the six-month survival probability for high-risk subjects reaching 0.4 in both cohorts and reaching approximately 0.7 in the diabetic vs. 0.85 in the non-diabetic subjects in the low-risk cohort, respectively. This proves that diabetes mellitus per se determines high COVID-19 mortality, also in the post-hospital follow-up period, which, however, seems to be less modified by the presence of additional comorbidities. Together with our other findings, all results point towards the significant increase in cardiogenic shock, myocardial injury, and acute heart failure with each additional point of C_2_HEST score in both cohorts, which indicates that cardio-metabolic burden plays a pivotal role in COVID-19 complication and notably increases mortality. In contrast, in relatively healthier subjects, not suffering from diabetes mellitus, the change from a low to high C_2_HEST stratum evokes more severe results. 

Even though the total mortality is significantly different between the C_2_HEST strata, the patient-reported symptoms, and their severity at the moment of admission in the diabetic cohort were similar apart from the frequency of peripheral edemas and wheezing occurrence. The ROC analysis indicates that C_2_HEST poorly predicts in-hospital deaths, which indicates the need to use other scales for this purpose. To date, the ABC2-SPH risk score was validated for predicting in-hospital mortality in COVID-19 and displayed better discrimination ability compared to other existing scores [28]. However, its effectiveness and similarly other scales predicting the mortality and severe course of the disease in the diabetic cohort require further detailed analysis. 

### Limitations

Our study has several limitations. First, our results are based on the data of a retrospective analysis of cases of patients hospitalized in a single center, which could affect the validity of our conclusions. Second, this study analyzed the whole COVID-19 cohort whose usefulness has not been verified under special circumstances, such as subpopulations with a specific comorbid condition. Finally, the data for this study were entirely from central Europe, which could potentially limit the generalizability of the risk score in other areas of the world. 

## 5. Conclusions

This study shows the usefulness and performance of the C_2_HEST score in predicting adverse COVID-19 outcomes in hospitalized subjects with type 2 diabetes mellitus. The simplicity of this scale, which can be calculated based on comorbidities, may address the medical needs in the risk stratification of COVID-19 subjects with diabetes mellitus.

## Figures and Tables

**Table 1:** Baseline demographics, clinical characteristics, and comorbidities in the diabetic and non-diabetic cohort.

Variables, Units	Low Risk (0–1)		Medium (2–3)		High Risk (>4)		ANOVA *p* Value		*p* Value for Post Hoc Analysis in Significant ANOVA L–M a L–H b M–H c	
Demographics	Diabetes N = 209	Non-Diabetes N = 1183	Diabetes N = 146	Non-Diabetes N = 337	Diabetes N = 118	Non-Diabetes N = 146	Diabetes	Non-Diabetes	Diabetes	Non- Diabetes
**Age, years** mean ± SD min–max N=	61.7 ± 11.7 17–74 209	49.4 ± 15.8 17–74 1183	75.3 ± 9.7 41–97 146	76.0 ± 12.5 29–100 337	76.9 ± 10.3 38–93 118	80.4 ± 8.2 50–100 146	<0.0001	<0.0001	<0.0001 ^a^ <0.0001 ^b^ 0.409 ^c^	<0.0001 ^a^ <0.0001 ^b^ 0.000016 ^c^
**Age ≥ 65 years** n, n (%)	106, (50.7)	268, (22.7)	130, (89.0)	284, (84.3)	103, (87.3)	141, (96.6)	<0.0001	<0.0001	<0.0001 ^a^ <0.0001 ^b^ 1.0 ^c^	<0.0001 ^a^ <0.0001 ^b^ 0.00074 ^c^
**Male sex** n, n (%)	129, (61.7)	597, (50.5)	69, (47.3)	134, (39.8)	62, (52.5)	71, (48.6)	0.022	0.0024	0.0287^a^ 0.4003 ^b^ 1.0 ^c^	0.002 ^a^ 1 ^b^ 0.2615 ^c^
**BMI. kg/m^2^** mean ± SD min–max N=	29.6 ± 5.7 17.1–42.4 65	27.8 ± 4.7 15.4–49.4 321	29.7 ± 3.8 23.0–36.7 27	28.9 ± 6.3 18.6–47.8 60	29.8 ± 6.3 19.6 -48.2 33	25.6 ± 4.6 16.4–34.9 32	0.9886	0.016	**N/A**	0.445 ^a^ 0.034 ^b^ 0.015 ^c^
**Normal body weight** (BMI = 18.5–24.9 kg/m^2^) n, n (%) N=	14, (21.5) 65	85, (26.5) 321	5, (18.5) 27	14, (23.3) 60	8, (24.2) 33	14, (43.8) 32	0.9961	0.0332	**N/A**	1 ^a^ 0.0304 ^b^ 0.0496 ^c^
**Underweight** (BMI < 18.5 kg/m^2^) n, n (%) N=	1, (1.5) 65	2, (0.6) 321	0, (0.0) 27	0, (0.0) 60	0, (0.0) 33	2, (6.3) 32				
**Overweight** (BMI = 25–29.9 kg/m^2^) n, n (%) N=	19, (29.2) 65	140, (43.6) 321	9, (33.3) 27	24, (40.0) 60	10, (30.3) 33	11, (34.4) 32				
**Obesity** (BMI ≥ 30 kg/m^2^) n, n (%) N=	31, (47.7) 65	94, (29.3) 321	13, (48.1) 27	22, (36.7) 60	15, (45.5) 33	5, (15.6) 32				
**Cigarette smoking, never/previous/current** n, n (%) N=	194, (92.8%)/6, (2.9%)/9, (4.3) 209	1122, (94.8%)/39, (3.3%)/22, (1.9%) 1183	131, (90.3%)/8, (5.5%), /6, (4.1%) 145	294, (87.8%)/24, (7.2%)/17, (5.1%) 335	98, (83.1%) /16, (13.6%) /4, (3.4%) 118	116, (80.0%)/17, (11.7%) /12, (8.3%) 145	0. 0081	<0.0001	1.0 ^a^ 0.0041 ^b^ 0.2695 ^c^	0.0002^a^ <0.0001 ^b^ 0.258 ^c^
**Hypertension,** n, n (%)	145, (69.4)	264, (22.3)	129, (88.4)	219, (65.0)	108, (91.5)	131, (89.7)	<0.0001	<0.0001	0.00015 ^a^ <0.0001 ^b^ 1.0 ^c^	<0.0001 ^a^ <0.0001 ^b^ <0.0001 ^c^
**Dyslipidemia,** n, n (%) N=	57, (62.6) 91	146, (54.7) 267	25, (48.1) 52	43, (39.1) 110	24, (43.6) 55	20, (34.5) 58	0.0545	0.0019	**N/A**	0.025 ^a^ 0.0025^b^ 1.0 ^c^
**Atrial fibrillation/flutter,** n, n (%)	16, (7.7)	31, (2.6)	35, (24)	68, (20.2)	54, (45.8)	77, (52.7)	<0.0001	<0.0001	<0.0001 ^a^ <0.0001 ^b^ 0.00098 ^c^	<0.0001 ^a^ <0.0001 ^b^ <0.0001 ^c^
**Previous coronary revascularization,** n, n (%)	2, (1.0)	4, (0.3)	14, (9.6)	21, (6.2)	55, (46.6)	50, (34.2)	<0.0001	<0.0001	0.00096 ^a^ <0.0001 ^b^ <0.0001 ^c^	<0.0001 ^a^ < 0.0001^b^ < 0.0001^c^
**Previous myocardial infarction** n, n (%)	2, (1.0)	9, (0.8)	28, (19.2)	32, (9.5)	55, (46.6)	57, (39.0)	<0.0001	<0.0001	<0.0001 ^a^ <0.0001 ^b^ <0.0001 ^c^	<0.0001 ^a^ <0.0001 ^b^ <0.0001 ^c^
**Heart failure,** n, n (%)	0, (0)	0, (0)	20, (13.7)	32, (9.5)	94, (79.7)	100, (68.5)	<0.0001	<0.0001	<0.0001 ^a^ <0.0001 ^b^ <0.0001 ^c^	<0.0001 ^a^ <0.0001 ^b^ <0.0001 ^c^
**Moderate/severe valvular heart disease or previous valve heart surgery,** n, n (%)	4, (1.9)	9, (0.8)	12, (8.2)	18, (5.3)	24, (20.3)	25, (17.1)	<0.0001	<0.0001	0.0316 ^a^ <0.0001 ^b^ 0.0226 ^c^	<0.0001 ^a^ <0.0001 ^b^ 0.00025 ^c^
**Peripheral artery disease,** n, n (%)	13, (6.2)	12, (1.0)	12, (8.2)	17, (5.0)	25, (21.2)	15, (10.3)	<0.0001	<0.0001	1 ^a^ 0.00032 ^b^ 0.01357 ^c^	<0.0001 ^a^ <0.0001 ^b^ 0.1354 ^c^
**Previous stroke/TIA,** n, n (%)	12, (5.7)	31, (2.6)	26, (17.8)	33, (9.8)	25, (21.2)	31, (21.2)	<0.0001	<0.0001	0.00172 ^a^ 0.00015 ^b^ 1 ^c^	<0.0001 ^a^ <0.0001 ^b^ 0.0034 ^c^
**Chronic kidney disease** n, n (%)	24, (11.5)	45, (3.8)	24, (16.4)	43, (12.8)	54, (45.8)	34, (23.3)	<0.0001	<0.0001	0.7072 ^a^ <0.0001 ^b^ <0.0001 ^c^	<0.0001 ^a^ <0.0001 ^b^ 0.017 ^c^
**Hemodialysis** n, n (%)	6, (2.9)	13, (1.1)	8, (5.5)	11, (3.3)	13, (11.09)	5, (3.4)	0.0095	0.0065	1 ^a^ 0.0164 ^b^ 0.463 ^c^	0.0314 ^a^ 0.1176 ^b^ 1 ^c^
**Asthma,** n, n (%)	9, (4.3)	43, (3.6)	9, (6.2)	13, (3.9)	16, (13.6)	3, (2.1)	0.8053	0.584676	**N/A**	**N/A**
**COPD** n, n (%)	1, (0.5)	5, (0.4)	9, (6.2)	15, (4.5)	16, (13.6)	25, (17.1)	<0.0001	<0.0001	0.0127 ^a^ <0.0001 ^b^ 0.2023 ^c^	<0.0001 ^a^ <0.0001 ^b^ <0.0001 ^c^
**Hypothyroidism** n, n (%)	10, (4.8)	62, (5.2)	17, (11.6)	49, (14.5)	33, (28.0)	28, (19.2)	<0.0001	<0.0001	0.0844 ^a^ <0.0001 ^b^ 0.004 ^c^	<0.0001 ^a^ <0.0001 ^b^ 0.7586 ^c^
**Hyperthyroidism** n, n (%)	0, (0)	4, (0.3)	1, (0.7)	9, (2.7)	0, (0)	6, (4.1)	0.5581	<0.0001	**N/A**	0.0011 ^a^ 0.0007 ^b^ 1.0 ^c^

Continuous variables are presented as: mean ± SD; range (minimum–maximum); and number of non-missing values. Categorized variables are presented as: a number with a percentage. Information about the numbers with valid values is provided in the left column. Abbreviations: N—valid measurements; n—number of patients with a parameter above the cut-off point; SD—standard deviation; BMI—body mass index; TIA—transient ischemic attack; COPD—chronic obstructive pulmonary disease; N/A—non-applicable; a—low- vs. medium-risk stratum; b—low- vs. high-risk stratum; c—medium- vs. high-risk stratum; red color text—statistically significant values. Statistically significant differences are marked in red color.

**Table 2:** Patient-reported symptoms, vital signs, and abnormalities measured during physical examination at hospital admission in the diabetic and non-diabetic cohorts.

Variables, Units	Low Risk (0–1)		Medium (2–3)		High Risk (>4)		ANOVA *p* Value		*p* Value for Post Hoc Analysis in Significant ANOVA L–M a L–H b M–H c	
Patient-Reported Symptoms	Diabetes N = 209	Non- Diabetes N = 1183	Diabetes N= 146	Non- Diabetes N = 337	Diabetes N = 118	Non- Diabetes N = 146	Diabetes	Non- Diabetes	Diabetes	Non- Diabetes
**Cough,** n, n (%)	52, (24.9)	392, (33.1)	34, (23.3)	89, (26.4)	25, (21.2)	38, (26)	0.749	0.0236	N/A	N/A
**Dyspnea,** n, n (%)	84, (40.2)	475, (40.2)	62, (42.5)	140, (41.5)	62, (52.5)	79, (54.1)	0.088	0.00546	N/A	1 ^a^ 0.0051 ^b^ 0.0431 ^c^
**Chest pain** n, n (%)	11, (5.3)	88, (7.4)	8, (5.5)	25, (7.4)	17, (14.4)	8, (5.5)	0.006	0.68528	N/A	N/A
**Hemoptysis** n, n (%)	3, (1.4)	6, (0.5)	0, (0)	2, (0.6)	2, (1.69)	2, (1.4)	0.271	0.3467	N/A	N/A
**Smell dysfunction** n, n (%)	2, (1)	57, (4.8)	2, (1.4)	8, (2.4)	3, (2.5)	2, (1.4)	0.552	0.03057	N/A	0.2137 ^a^ 0.2698 ^b^ 1 ^c^
**Taste dysfunction** n, n (%)	3, (1.4)	45, (3.8)	3, (2.1)	7, (2.1)	4, (3.4)	3, (2.1)	0.467	0.2337	N/A	N/A
**Abdominal pain** n, n (%)	21, (10.1)	83, (7.0)	7, (4.8)	19, (5.6)	5, (4.2)	12, (8.2)	0.065	0.53339	N/A	N/A
**Diarrhea** n, n (%)	14, (6.7)	61, (5.2)	9, (6.2)	24, (7.1)	4, (3.4)	15, (10.3)	0.446	0.03065	N/A	N/A
**Nausea/vomiting** n, n (%)	8, (3.8)	48, (4.1)	11, (7.5)	16, (4.7)	5, (4.2)	9, (6.2)	0.262	0.4694	N/A	N/A
**Body temperature**, °C mean ± SD min–max N=	36.9 ± 0.82 34.4–39.5 110	37.1 ± 0.89 35.0–40.5 678	37 ± 1 35–40 75	36.9 ± 0.87 35.5–40.0 154	36.8 ± 0.7 35.2–39.3 63	37.0 ± 0.97 35.9–40.0 71	0.471	0.07369	N/A	N/A
**Heart rate,** beats/minute mean ± SD min–max N=	87.1 ± 17.2 48–150 160	86.3 ± 15.41 48–160 863	85.5 ± 16.2 50–150 122	83.5 ± 16.7 50–160 257	84.6 ± 17.5 47–140 109	84.7 ± 18.97 36–150 121	0.49	0.0486	N/A	0.045^a^ 0.626 ^b^ 0.84 ^c^
**Respiratory rate,** breaths/minute mean ± SD min–max N=	21.1 ± 9.1 12–50 35	17.8 ± 4.65 12–40 167	18 ± 4 12–28 20	18.9 ± 6.05 12–45 44	19.9 ± 7.8 12–50 24	18.9 ± 3.66 12–25 19	0.196	0.3055	N/A	N/A
**Systolic blood pressure**, mmHg mean ± SD min–max N=	132.9 ± 21.6 60–204 160	130.2 ± 21.22 60–240 855	134.3 ± 38.8 50–270 121	134.1 ± 23.39 60–210 256	137.5 ± 23.4 86–210 111	132.6 ± 26.11 70–205 123	0.253	0.05307	N/A	N/A
**Diastolic blood pressure**, mmHg mean ± SD min–max N=	78.8 ± 13.6 40–125 159	78.5 ± 12.5 40–150 853	79.2 ± 14.3 50–150 117	77.6 ± 13.1 45–157 255	76 ± 14.8 44–143 111	76.0 ± 15.66 40–120 123	0.190	0.19414	N/A	N/A
**Pulse pressure** mean ± SD min–max N=	54.6 ± 16 15–110 159	51.9 ± 15.28 11–136 853	57.5 ± 18.6 20–120 117	56.9 ± 18.16 20–120 254	61.6 ± 18 30–130 111	56.6 ± 19.47 20–120 123	0.0049	<0.0001	0.36 ^a^ 0.003 ^b^ 0.212 ^c^	0.0002 ^a^ 0.03^b^ 0.987 ^c^
**SpO_2_ on room air,** % (FiO_2_ = 21%) mean ± SD min–max N=	91.8 ± 6.8 56–100 106	92.9 ± 7.2 48–100 690	88.8 ± 10.2 50–100 89	90.0 ± 9.45 50–99 187	91.5 ± 7.8 60–99 66	88.8 ± 9.13 50–99 92	0.059	<0.0001	N/A	0.0005^a^ 0.0003^b^ 0.559 ^c^
**SpO_2_ < 90%,** n, n (%)	29, (27.4)	153, (22.2)	37, (41.6)	63, (33.7)	19, (28.8)	36, (39.1)	0.081	<0.0001	N/A	0.00496^a^ 0.0018^b^ 1 ^c^
**GCS,** points mean ± SD min–max N=	14.7 ± 1.3 6–15 77	14.6 ± 1.88 3–15 484	14.2 ± 2 3–15 56	14.6 ± 1.52 3–15 130	14.2 ± 2.6 3–15 48	14.0 ± 2.38 3–15 66	0.202	0.15588	N/A	N/A
**Cracles** n, n (%)	33, (15.8)	119, (10.1)	38, (26)	61, (18.1)	33, (28)	32, (21.9)	0.014	<0.0001	N/A	0.00025 ^a^ 0.0001 ^b^ 1 ^c^
**Wheezing** n, n (%)	15, (7.2)	79, (6.7)	20, (13.7)	35, (10.4)	32, (27.1)	35, (24.0)	<0.0001	<0.0001	0.1941 ^a^ <0.0001 ^b^ 0.0305 ^c^	0.0917 ^a^ <0.0001 ^b^ 0.00052 ^c^
**Pulmonary congestion** n, n (%)	37, (17.7)	145, (12.3)	39, (26.7)	62, (18.4)	39, (33.1)	36, (24.7)	0.006	<0.0001	N/A	0.0149 ^a^ 0.0002 ^b^ 0.443 ^c^
**Peripheral edema** n, n (%)	14, (6.7)	61, (5.2)	24, (16.4)	35, (10.4)	29, (24.6)	23, (15.8)	<0.0001	<0.0001	0.0181 ^a^ <0.0001 ^b^ 0.4113 ^c^	0.0024 ^a^ <0.0001 ^b^ 0.3899 ^c^
**Hemiplegia/hemiparesis** n, n (%)	6, (2.9)	23, (1.9)	10, (6.9)	13, (3.9)	6, (5.1)	12, (8.2)	0.209	0.0002	N/A	0.1931 ^a^ 0.0005 ^b^ 0.2122 ^c^
**VES-13,** points mean ± SD min–max N=	5 ± 3.4 1–9 8	3.8 ± 2.63 1–9 20	6.28 ± 3.6 1–12 18	5.1 ± 3.03 1–10 19	6.2 ± 2.66 3–10 10	6.5 ± 3.2 3–13 14	0.671	0.045	N/A	0.363 ^a^ 0.038 ^b^ 0.394 ^c^

Continuous variables are presented as: mean ± SD; range (minimum–maximum); and the number of non-missing values. Categorized variables are presented as: a number with a percentage. Information about the numbers with valid values is provided in the left column. Abbreviations: N—valid measurements; n—number of patients with a parameter above the cut-off point; SD—standard deviation; GCS—Glasgow Coma Scale; VES—Vulnerable Elders Survey; N/A—non-applicable; a—low- vs. medium-risk stratum; b—low- vs. high-risk stratum; c—medium- vs. high-risk stratum; red color text—statistically significant values. Statistically significant differences are marked in red color.

**Table 3:** Applied treatment and procedures in the diabetic and non-diabetic cohorts after C_2_HEST risk stratification.

Variables	Low Risk (0–1)		Medium (2–3)		High Risk (>4)		ANOVA *p* Value		*p* Value for Post Hoc Analysis in Significant ANOVA L–M a L–H b M–H c	
	Diabetes N = 209	Non- Diabetes N = 1183	Diabetes N= 146	Non- Diabetes N = 337	Diabetes N = 118	Non- Diabetes N = 146	Diabetes	Non- Diabetes	Diabetes	Non- Diabetes
**Applied treatment and procedures**										
The most advanced respiratory support applied during the hospitalization							0.0712	<0.0001	N/A	0.0229 ^a^ <0.0001 ^b^ 0.0066 ^c^
**no oxygen**										
n, n (%)	79, (37.8)	648, (54.9)	45, (30.8)	153, (45.4)	37, (31.4)	47, (32.2)				
**low flow oxygen support**										
n, n (%)	79, (37.8)	365, (30.9)	61, (41.8)	125, (37.1)	49, (41.5)	72, (49.3)				
**high flow nasal cannula**										
non-invasive ventilation										
n, n (%)	15, (3.2)	67, (5.7)	20, (4.2)	32, (9.5)	18, (3.8)	20, (13.7)				
**invasive ventilation**										
n, n (%)	36, (17.2)	101, (8.6)	20, (13.7)	27, (8.0)	14, (11.9)	7, (4.8)				
Oxygenation parameters from the period of qualification for advanced respiratory support: SpO_2_, % mean ± SD (min–max) N=	88.4 ± 8.5 (60–98) 54	90.9 ± 7.8 (50–100) 345	86.6 ± 10.8 (57–99) 47	86.9 ± 9.0 (55–99) 82	87.6 ± 8.0 (60–98) 42	83.6 ± 11.4 (59–99) 44	0.6374	<0.0001	N/A	0.0009^a^ 0.0004^b^ 0.224 ^c^
Therapy with catecholamines, n, n (%)	33, (15.8)	95, (8.0)	15, (10.3)	28, (8.3)	22, (18.6)	19, (13.0)	0.1411	0.124667	N/A	N/A
Coronary revascularization or/and an indication for coronary revascularization, n, n (%)	4, (1.9)	4, (0.3)	4, (2.7)	6, (1.8)	5, (4.2)	2, (1.4)	0.4235	0.0092	N/A	0.0317^a^ 0.4015 ^b^ 1 ^c^
Hemodialysis, n, n (%)	15, (7.2)	31, (2.6)	7, (4.8)	5, (1.5)	8, (6.8)	3, (2.1)	0.6466	0.5311	N/A	N/A

Continuous variables are presented as: mean ± S; range (minimum–maximum); and the number of non-missing values. Categorized variables are presented as: a number with a percentage. Information about the numbers with valid values is provided in the left column. Abbreviations: N—valid measurements; n—number of patients with a parameter above cut-off point; SD—standard deviation; ANOVA—analysis of variance; N/A—non-applicable; a—low- vs. medium-risk stratum; b—low- vs. high-risk stratum; c—medium- vs. high-risk stratum. Statistically significant differences are marked in red color.

**Table 4:** Total and in-hospital all-cause mortality in the C_2_HEST risk strata in the diabetic and non-diabetic cohort.

Variables	Low Risk (0–1)		Medium (2–3)		High Risk (>4)		ANOVA *p* Value		*p* Value for Post Hoc Analysis in Significant ANOVA L–M a L–H b M–H c	
	Diabetes N = 209	Non-Diabetes N = 1183	Diabetes N= 146	Non-Diabetes N = 337	Diabetes N = 118	Non-Diabetes N = 146	Diabetes	Non-Diabetes	Diabetes	Non- Diabetes
**All-cause mortality rate**										
In-hospital mortality, n, n (%)	32, (15.3)	85, (7.2)	42, (28.8)	65, (19.3)	41, (34.7)	54, (37.0)	0.00014	<0.0001	0.0099 0.0003 1.0 ^c^	<0.0001 ^a^ <0.0001 ^b^ 0.00017 ^c^
3-month mortality, n, n (%) N=	51, (24.4) 201	150, (12.7) 1116	69, (47.3) 143	125, (37.1) 323	63, (53.4) 117	81, (55.5) 143	<0.0001	<0.0001	<0.0001 ^a^ <0.0001 ^b^ 1.0 ^c^	<0.0001 ^a^ <0.0001 ^b^ 0.0008 ^c^
6-month mortality, n, n (%) N=	55, (37.2) 118	158, (22.3) 447	71, (60.7) 109	133, (51.4) 214	68, (64.8) 91	85, (68.0) 116	<0.0001	<0.0001	0.0007^a^ <0.0001 ^b^ 0.5682 ^c^	<0.0001 ^a^ <0.0001 ^b^ 0.0088 ^c^
**Hospitalization**										
Duration of hospitalization, days (distribution to be verified) mean ± SD (min–max)	16.5 ± 17.7 (1–126)	1.5 ± 12.4 (1–131)	14.4 ± 14.6 (1–72)	12.8 ± 13.3 (1–124)	17.8 ± 18.2 (1–121)	15.3 ± 14.0 (1–82)	0.2293	<0.0001		0.011^a^ 0.0004^b^ 0.181 ^c^
End of hospitalization							0.0003	<0.0001	0.0063 ^a^ 0.0004 ^b^ 1.0 ^c^	<0.0001 ^a^ <0.0001 ^b^ 0.0019 ^c^
**death**										
n, n (%)	32, (15.3)	85, (7.2)	42, (28.8)	65, (19.3)	41, (34.7)	54, (37)				
**discharge to home—full recovery**										
n, n (%)	120, (57.4)	851, (71.9)	56, (38.4)	164, (48.7)	43, (36.4)	56, (38.4)				
**transfer to another hospital—worsening)**										
n, n (%)	28, (13.4)	111, (9.4)	24, (16.4)	70, (20.8)	20, (16.9)	23, (15.8)				
**transfer to another hospital—in recovery**										
n, n (%)	29, (13.9)	136, (11.5)	24, (16.4)	38, (11.3)	14, (11.9)	13, (8.9)				

Categorized variables are presented as: a number with a percentage. Abbreviations: N—valid measurements; n—number of patients with a parameter above the cut-off point; SD—standard deviation; ANOVA—analysis of variance; N/A—non-applicable; a—low- vs. medium-risk stratum; b—low- vs. high-risk stratum; c—medium- vs. high-risk stratum. Statistically significant differences are marked in red color.

**Table 5:** The total all-cause death hazard ratios for C_2_HEST risk stratification in the diabetic cohort.

	Diabetics			Non-Diabetics		
Total Deaths	HR	95% CI	*p*-Value	HR	95% CI	*p*-Value
**Overall**	**1.25**	1.163–1.341	**<0.0001**	**1.45**	1.382–1.525	**<0.0001**
***Risk strata***						
**Medium- vs. low-risk**	**2.34**	1.658–3.315	**<0.0001**	**3.51**	2.795–4.414	**<0.0001**
**High- vs. low-risk**	**2.84**	1.999–4.0329	**<0.0001**	**6.0**	4.628–7.794	**<0.0001**

Bold text—statistically significant values.

**Table 6:** The in-hospital all-cause death hazard ratios for C_2_HEST risk stratification in the diabetic cohort.

	Diabetics			Non-Diabetics		
Total Deaths	HR	95% CI	*p*-Value	HR	95% CI	*p*-Value
**Overall**	**1.19**	1.081–1.314	**<0.0005**	1.294	1.207–1.387	**<0.0001**
***Risk strata***						
**Medium- vs. low-risk**	**2.16**	1.363–3.437	**0.00106**	**2.135**	-	**-**
**High- vs. low-risk**	**2.11**	1.329–3.356	**0.00155**	**3.345**	-	**-**

Bold text—statistically significant values.

**Table 7:** Clinical non-fatal events and hospitalization outcomes in the C_2_HEST risk strata in the diabetic and non-diabetic cohort.

Variables	Low Risk (0–1)		Medium (2–3)		High Risk (>4)		ANOVA *p* Value		*p* Value for Post Hoc Analysis in Significant ANOVA L–M a L–H b M–H c	
	Diabetes N = 209	Non-Diabetes N = 1183	Diabetes N= 146	Non-Diabetes N = 337	Diabetes N = 118	Non-Diabetes N = 146	Diabetes	Non- Diabetes	Diabetes	Non- Diabetes
Aborted cardiac arrest, n, n (%)	2, (1.0)	13, (1.1)	0, (0.0)	3, (0.9)	4, (3.4)	2, (1.4)	0.0573	0.8520	N/A	N/A
Shock, n, n (%)	29, (13.9)	76, (6.4)	14, (9.6)	29, (8.6)	16, (13.6)	16, (11.0)	0.4457	0.0782	N/A	N/A
Hypovolemic shock, n, n (%)	5, (2.4)	17, (1.4)	3, (2.1)	4, (1.2)	1, (0.8)	5, (3.4)	0.6946	0.1623	N/A	N/A
Cardiogenic shock, n, n (%)	2, (1.0)	5, (0.4)	4, (2.7)	7, (2.1)	8, (6.8)	6, (4.1)	0.0132	0.00011	0.7008 ^a^ 0.01599 ^b^ 0.4287 ^c^	0.0208^a^ 0.0014^b^ 0.6787 ^c^
Septic shock, n, n (%)	27, (12.9)	58, (4.9)	10, (6.8)	18, (5.3)	12, (10.2)	9, (6.2)	0.1812	0.7878	N/A	N/A
Venous thromboembolic disease, n, n (%)	13, (6.2),	68, (5.7)	9, (6.2)	21, (6.2)	3, (2.5)	12, (8.2)	0.3067	0.493	N/A	N/A
Pulmonary embolism, n, n (%)	4, (1.9)	28, (2.4)	2, (1.4)	7, (2.1)	3, (2.5)	4, (2.7)	0.98	0.7257	N/A	N/A
Myocardial infarction, n, n (%)	4, (1.9)	4, (0.3)	4, (2.7)	6, (1.8)	4, (3.4)	3, (2.1)	0.657	0.0049	N/A	0.0317^a^ 0.0978 ^b^ 1 ^c^
Myocardial injury, 3x, n, n (%) N	31, (24.6) N = 126	78, (14.4) N = 542	35, (39.3) N = 89	60, (28.7) N = 209	36, (41.4) N = 87	46, (48.4) N = 95	0.0165	<0.0001	0.0934 ^a^ 0.0438 ^b^ 1.0 ^c^	<0.0001 ^a^ <0.0001 ^b^ 0.0039 ^c^
Acute heart failure, n, n (%)	2(1.0)	6, (0.5)	9, (6.2)	13, (3.9)	23, (19.5)	23, (15.8)	<0.0001	<0.0001	0.04 ^a^ <0.0001 ^b^ 0.0056 ^c^	<0.0001 ^a^ <0.0001 ^b^ <0.0001 ^c^
Stroke/TIA, n, n (%)	4, (1.9)	13, (1.1)	8, (5.5)	10, (30.0)	2, (1.7)	3, (2.1)	0.1361	0.0347	N/A	0.062 ^a^ 1.0 ^b^ 1.0 ^c^
Pneumonia, n, n (%)	127, (60.8)	545, (46.1)	90, (61.6)	210, (62.3)	77, (65.3)	102, (69.9)	0.7155	<0.0001	N/A	<0.0001^a^ <0.0001^b^ 0.409 ^c^
Complete respiratory failure, n, n (%) N	17, (45.9) N = 37	39, (47.0) N = 83	20, (62.5) N = 32	26, (46.4) N = 56	21, (70) N = 30	22, (62.9) N = 35	0.1195	0.2344	N/A	N/A
SIRS, n, n (%) N	23, (11.2) N = 206	116, (10.3) N = 1121	17, (11.6) N = 146	25, (97.5) N = 334	17, (14.4) N = 118	19, (13.1) N = 145	0.675	0.132	N/A	N/A
Sepsis, n, n (%) N	2, (2.6) N = 77	7, (1.4) N = 484	4, (7.3) N = 55	2, (1.6) N = 122	2, (3.6) N = 55	5, (7.8) N = 64	0.4404	0.0109	N/A	N/A
Acute kidney injury, n, n (%)	28, (13.4)	81, (6.8)	27, (18.5)	39, (11.6)	31, (26.3)	25, (17.1)	0.0149	<0.0001	0.7422 ^a^ 0.0175 ^b^ 0.5139 ^c^	0.0194 ^a^ <0.0001^b^ 0.396 ^c^
Acute liver dysfunction, n, n (%) N	6, (3.0) N = 198	24, (2.3) N = 1034	5, (3.7) N = 136	17, (5.3) N = 320	7, (6.4) N = 109	7, (5.2) N = 134	0.3388	0.001	N/A	0.0408 ^a^ 0.2311 ^b^ 1 ^c^
Multiple organ dysfunction syndrome, n, n (%)	2, (1.0)	19, (1.6)	3, (2.1)	5, (1.5)	5, (4.2)	3, (2.1)	0.1421	0.8547	N/A	N/A
Lactic acidosis (on admission), n, n (%) N	3, (9.7) N = 31	6, (8.2) N = 73	2, (7.1) N = 28	3, (5.9) N = 51	6, (22.2) N = 27	2, (6.3) N = 32	0.2581	0.9199	N/A	N/A
Hyperlactatemia (on admission) n, n (%) N	20, (64.5) N = 31	58, (79.5) N = 73	17, (60.7) N = 28	35, (68.6) N = 51	16, (59.3) N = 27	21, (65.2) N = 32	0.9124	0.2317	N/A	N/A
Bleeding, n (%) n, n (%)	15, (7.2)	48, (4.1)	9, (6.2)	16, (4.7)	11, (9.3)	14, (9.6)	0.6137	0.0116	N/A	1 ^a^ 0.0162 ^b^ 0.2066 ^c^
Intracranial bleeding, n, n (%)	3, (1.4)	9, (0.8)	3, (2.1)	5, (1.5)	0, (0.0)	1, (0.7)	0.3846	0.3794	N/A	N/A
Respiratory tract bleeding, n, n (%)	6, (2.9)	17, (1.4)	3, (2.1)	1, (0.3)	4, (3.4)	3, (2.1)	0.774	0.1106	N/A	N/A
Gastrointestinal bleeding, n, n (%)	7, (3.3)	13, (1.1)	2, (1.4)	7, (2.1)	5, (4.2)	7, (4.8)	0.4699	0.0031	N/A	0.4279 ^a^ 0.0047 ^b^ 0.3529 ^c^
Urinary tract bleeding, n, n (%)	3, (1.4)	6, (0.5)	2, (1.4)	2, (0.6)	2, (1.7)	3, (2.1)	1.0	0.0955	N/A	N/A

Continuous variables are presented as: mean ± SD range (minimum–maximum) and the number of non-missing values. Categorized variables are presented as: a number with a percentage. Abbreviations: N—valid measurements; n—number of patients with a parameter above the cut-off point; SD—standard deviation; ANOVA—analysis of variance; N/A—not applicable; a—low- vs. medium-risk stratum; b—low- vs. high-risk stratum; c—medium- vs. high-risk stratum; red color text—statistically significant values. Statistically significant differences are marked in red color.

## Data Availability

The datasets used and/or analyzed during the present study are available from the corresponding author upon reasonable request.

## Supplementary Materials

The following supporting information can be downloaded at: https://www.mdpi.com/article/10.3390/jcm11030873/s1, Table S1. Baseline characteristics of the diabetes and non-diabetes cohort - treatment applied before hospitalization. Table S2. Laboratory parameters measured during the hospitalisation in the diabetic and non-diabetic cohort. Table S3. Therapies applied during the hospitalisation in the diabetic and non-diabetic cohort. Table S4. The Log-rank statistics for matching the C_2_HEST risk strata for total mortality in the diabetic and non-diabetic cohort. Table S5. The Log-rank statistics for matching the C_2_HEST risk strata for in-hospital mortality in the diabetic and non-diabetic cohort. Table S6. Odds ratios for quantifying the strength of the association between CH2EST-score and study endpoints and adverse events in the diabetic and non-diabetic cohort. Table S7. Discriminatory performance of the C_2_HEST score on the clinical events in the diabetic and non-diabetic cohort. Table S8. The in-hospital all-cause-death Hazard Ratios for modified C_2_HEST risk stratification—Replace “thyroid disease” by “hypothyroidism” and “age > 75 years” by “age > 65 years” in the diabetic and non-diabetic cohort. Table S9. Odds ratios for quantifying the strength of the association between the modified CH2EST-score and study endpoints and adverse events—replacing “thyroid disease” with “hypothyroidism” and cut-off point for age to “>65 years” as scoring items in the diabetic and non-diabetic cohort. Table S10. Components of C_2_HEST score and the risk of outcomes in univariate Cox proportional hazard model (all-cause death) and competing risk regression model (other outcomes) in diabetes cohort.

## References

[1] Tang X., Du R.H., Wang R., Cao T.Z., Guan L.L., Yang C.Q., Zhu Q., Hu M., Li X.Y., Li Y. (2020). Comparison of Hospitalized Patients with ARDS Caused by COVID-19 and H1N1. Chest.

[2] Zhou F., Yu T., Du R., Fan G., Liu Y., Liu Z., Xiang J., Wang Y., Song B., Gu X. (2020). Clinical course and risk factors for mortality of adult inpatients with COVID-19 in Wuhan, China: A retrospective cohort study. Lancet.

[3] Janus A., Szahidewicz-Krupska E., Mazur G., Doroszko A. (2016). Insulin Resistance and Endothelial Dysfunction Constitute a Common Therapeutic Target in Cardiometabolic Disorders. Mediat. Inflamm..

[4] Hussain A., Bhowmik B., do Vale Moreira N.C. (2020). COVID-19 and diabetes: Knowledge in progress. Diabetes Res. Clin. Pract..

[5] Whyte M.B., Vas P., Heiss C., Feher M.D. (2020). The contribution of diabetic micro-angiopathy to adverse outcomes in COVID-19. Diabetes Res. Clin. Pract..

[6] Katsiki N., Gómez-Huelgas R., Mikhailidis D.P., Pérez-Martínez P. (2021). Narrative review on clinical considerations for patients with diabetes and COVID-19: More questions than answers. Int. J. Clin. Pract..

[7] Liang W., Liang H., Ou L., Chen B., Chen A., Li C., Li Y., Guan W., Sang L., Lu J. (2020). Development and validation of a clinical risk score to predict the occurrence of critical illness in hospitalized patients with COVID-19. JAMA Intern. Med..

[8] Robin X., Turck N., Hainard A., Tiberti N., Lisacek F., Sanchez J.C., Müller M. (2011). pROC: An open-source package for R and S+ to analyze and compare ROC curves. BMC Bioinform..

[9] Therneau T. (2020). A Package for Survival Analysis in R; R Package Version 3.2-7. Mayo Foundation for Medical Education and Research.

[10] Hothorn T., Hornik K., Van De Wiel M.A., Zeileis A. (2006). A lego system for conditional inference. Am. Stat..

[11] Muenchow J., Schratz P., Brenning A. (2017). QGIS: Integrating R with QGIS for statistical geocomputing. R J. (Scopus).

[12] Seiglie J., Platt J., Cromer S.J., Bunda B., Foulkes A.S., Bassett I.V. (2020). Diabetes as a Risk Factor for Poor Early Outcomes in Patients Hospitalized With COVID-19. Diabetes Care.

[13] Huang C., Wang Y., Li X., Ren L., Zhao J., Hu Y. (2020). Clinical features of patients infected with 2019 novel coronavirus in Wuhan China. Lancet.

[14] Wang D., Hu B., Hu C., Zhu F., Liu X., Zhang J. (2020). Clinical Characteristics of 138 Hospitalized Patients With 2019 Novel Coronavirus-Infected Pneumonia in Wuhan China. JAMA.

[15] Abdi A., Jalilian M., Ahmadi P., Vlaisavljevic Z. (2020). Diabetes and COVID-19: A systematic review on the current evidences. Diabetes Res. Clin. Pract..

[16] Huang I., Lim M.A., Pranata R. (2020). Diabetes mellitus is associated with increased mortality and severity of disease in COVID-19 pneumonia—a systematic review, meta-analysis, and meta-regression: Diabetes and COVID-19. Diabetes Metab. Syndr. Clin. Res. Rev..

[17] Volpe C.M.O., Villar-Delfino P.H., Dos Anjos P.M.F., Nogueira-Machado J.A. (2018). Cellular death, reactive oxygen species (ROS) and diabetic complications. Cell Death Dis..

[18] Hu R., Xia C.-Q., Butfiloski E., Clare-Salzler M. (2018). Effect of high glucose on cytokine production by human peripheral blood immune cells and type I interferon signaling in monocytes: Implications for the role of hyperglycemia in the diabetes inflammatory process and host defense against infection. Clin. Immunol..

[19] Guo J., Lin W.W., Zucker J.E., Nandakumar R., Uhlemann A.C., Wang S., Shivakoti R. (2022). Inflammation and mortality in COVID-19 hospitalized patients with and without type 2 diabetes. J. Clin. Endocrinol. Metab..

[20] Sarkar S., Das D., BorsinghWann S., Kalita J., Manna P. (2021). Is diabetes mellitus a wrongdoer to COVID-19 severity?. Diabetes Res. Clin. Pract..

[21] Harbuwono D.S., Handayani D.O.T.L., Wahyuningsih E.S., Supraptowati N., Ananda, Kurniawan F., Wafa S., Kristanti M., Pantoro N.I., Sinto R. (2021). Impact of diabetes mellitus on COVID-19 clinical symptoms and mortality: Jakarta’s COVID-19 epidemiological registry. Prim. Care Diabetes.

[22] Kumar A., Arora A., Sharma P., Anikhindi S.A., Bansal N., Singla V., Khare S., Srivastava A. (2020). Is diabetes mellitus associated with mortality and severity of COVID-19? A meta-analysis. Diabetes Metab. Syndr. Clin. Res. Rev..

[23] Kulcsar K.A., Coleman C.M., Beck S.E., Frieman M.B. (2019). Comorbid diabetes results in immune dysregulation and enhanced disease severity following MERS-CoV infection. JCI Insight.

[24] Alraddadi B.M., Watson J.T., Almarashi A., Abedi G.R., Turkistani A., Sadran M., Housa A., AlMazroa M.A., Alraihan N., Banjar A. (2016). Risk Factors for Primary Middle East Respiratory Syndrome Coronavirus Illness in Humans, Saudi Arabia, 2014. Emerg. Infect. Dis..

[25] Daryabor G., Atashzar M.R., Kabelitz D., Meri S., Kalantar K. (2020). The Effects of Type 2 Diabetes Mellitus on Organ Metabolism and the Immune System. Front. Immunol..

[26] Kumar N.P., Sridhar R., Nair D., Banurekha V.V., Nutman T.B., Babu S. (2015). Type 2 diabetes mellitus is associated with altered CD8+ T and natural killer cell function in pulmonary tuberculosis. Immunology.

[27] Hill M.A., Mantzoros C., Sowers J.R. (2020). Commentary: COVID-19 in patients with diabetes. Metabolism.

[28] Marcolino M.S., Pires M.C., Ramos L.E.F., Silva R.T., Oliveira L.M., Carvalho R.L., Mourato R.L.S., Sánchez-Montalvá A., Raventós B., Anschau F. (2021). ABC2-SPH risk score for in-hospital mortality in COVID-19 patients: Development, external validation and comparison with other available scores. Int. J. Infect. Dis..

