# Supplementary material for: Usefulness of the C2HEST Score in Predicting the Clinical Outcomes of COVID-19 in Diabetic and Non-Diabetic Cohorts
Source: J Clin Med. 2022 Feb 7;11(3):873. doi: 10.3390/jcm11030873 (PMC8836928; doi:10.3390/jcm11030873)
Supplement: Supplementary file 1 [file jcm-11-00873-s001.zip › jcm-1587041-supplementary.pdf]

Supplementary Tables

Supplementary Table S1. Baseline characteristics of the diabetic and non-diabetic cohort - treatment applied before hospitalization

Treatment applied before hospitalization	Low risk [0-1]		Medium [2-3]		High risk [≥4]		ANOVA P value		P value for post-hoc analysis in significant ANOVA L-M a L-H b M-H c	
	Diabetes N=209	Non-Diabetes N=1183	Diabetes N= 146	Non-Diabetes N= 337	Diabetes N=118	Non-Diabetes N= 146	Diabetes	Non-Diabetes	Diabetes	Non-Diabetes
**ACEI,**<br>n<br>n (%)	49<br>(23,4)	64<br>(5,4)	38<br>(26,0)	80<br>(23,7)	48<br>(40,7)	60<br>(41,1)	0,003	<0.0001	1[a]<br>0,0049[b]<br>0,0501[c]	<0.0001[a]<br><0.0001[b]<br>0,00053[c]
**ARB,**<br>n<br>n (%)	24<br>(11,5)	51<br>(4,3)	9<br>(6,2)	29<br>(8,6)	12<br>(10,2)	17<br>(11,6)	0,2342	<0.0001	N/A	0,0088[a]<br>0,00097[b]<br>1[c]
**MRA,**<br>n<br>n (%)	7<br>(3,3)	11<br>(0,9)	13<br>(8,9)	18<br>(5,3)	24<br>(20,3)	23<br>(15,8)	<0.0001	<0.0001	0,1366[a]<br><0.0001[b]<br>0,0391[c]	<0.0001[a]<br><0.0001[b]<br>0,00095[c]
**β-blocker**<br>n<br>n (%)	70<br>(33,5)	123<br>(10,4)	70<br>(47,9)	105<br>(31,2)	72<br>(61,0)	79<br>(54,1)	<0.0001	<0.0001	0,0255[a]<br><0.0001[b]<br>0,1385[c]	<0.0001[a]<br><0.0001[b]<br><0.0001[c]
**Calcium channel blocker (dihydropiridines)**<br>n<br>n (%)	41<br>(19,6)	61<br>(5,2)	30<br>(20,5)	52<br>(15,4)	35<br>(29,7)	37<br>(25,3)	0,0909	<0.0001	N/A	<0.0001[a]<br><0.0001[b]<br>0,0425[c]
**α-adrenergic blocker,**<br>n<br>n (%)	20<br>(9,6)	25<br>(2,1)	9<br>(6,2)	24<br>(7,1)	20<br>(16,9)	18<br>(12,3)	0,0148	<0.0001	N/A	<0.0001[a]<br><0.0001[b]<br>0,2735[c]
**Amiodarone**<br>n<br>n (%)	1<br>(0,5)	0<br>(0,0)	1<br>(0,7)	1<br>(0,3)	1<br>(0,8)	0<br>(0,0)	1	0,29	N/A	N/A
**Thiazide or thiazide-like diuretic**<br>n<br>n (%)	28<br>(13,4)	38<br>(3,2)	18<br>(12,3)	28<br>(8,3)	16<br>(13,6)	16<br>(11,0)	0,9444	<0.0001	N/A	0,00029[a]<br><0.0001[b]<br>1[c]
**Loop diuretic,**<br>n<br>n (%)	17<br>(8,1)	22<br>(1,9)	26<br>(17,8)	37<br>(11,0)	46<br>(39,0)	29<br>(19,9)	<0.0001	<0.0001	0,0293[a]<br><0.0001[b]<br>0,00064[a]	<0.0001[a]<br><0.0001[b]<br>0,041[c]
**Statin,**<br>n<br>n (%)	46<br>(22,0)	54<br>(4,6)	47<br>(32,2)	69<br>(20,5)	58<br>(49,2)	61<br>(41,8)	<0.0001	<0.0001	0,1288[a]<br><0.0001[b]<br>0,0226[c]	<0.0001[a]<br><0.0001[b]<br><0.0001[c]
**Acetylsalicylic acid,**<br>n<br>n (%)	35<br>(16,7)	44<br>(3,7)	38<br>(26,0)	53<br>(15,7)	39<br>(33,1)	37<br>(25,3)	2,83x10^-3	<0.0001	0,138[a]<br>0,0035[b]<br>0,7983[c]	<0.0001[a]<br><0.0001[b]<br>0,0541[c]
**The second antiplatelet drug,**<br>n<br>n (%)	4<br>(1,9)	3<br>(0,3)	3<br>(2,1)	7<br>(2,1)	14<br>(11,9)	6<br>(4,1)	<0.0001	<0.0001	1[a]<br>0,0012[b]<br>0,0088[c]	0,0057[a]<br>0,00011[b]<br>0,8205[c]
**LMWH**<br>n<br>n (%)	17<br>(8,1)	56<br>(4,7)	15<br>(10,3)	25<br>(7,4)	8<br>(6,8)	16<br>(11,0)	0,5829	0,0038	N/A	0,2164[a]<br>0,0098[b]<br>0,8083[c]
**VKA,**<br>n<br>n (%)	1<br>(0,5)	9<br>(0,8)	4<br>(2,7)	8<br>(2,4)	11<br>(9,3)	10<br>(6,8)	0,00018	<0.0001	0,4912[a]<br>0,00025[b]<br>0,0915[c]	0,1002[a]<br><0.0001[b]<br>0,0971[c]
**NOAC,**<br>n<br>n (%)	8<br>(3,8)	9<br>(0,8)	10<br>(6,8)	26<br>(7,7)	25<br>(21,2)	25<br>(17,1)	<0.0001	<0.0001	0,907558[a]<br><0.0001[b]<br>0,0037[c]	<0.0001[a]<br><0.0001[b]<br>0,0102[c]
**Insulin,**<br>n<br>n (%)	57<br>(27,3)	2<br>(0,2)	27<br>(18,5)	2<br>(0,6)	39<br>(33,1)	1<br>(0,7)	0,0235	0,17708	0,2212[a]<br>0,988[b]<br>0,0303[c]	N/A
**Metformin,**<br>n<br>n (%)	98<br>(46,9)	2<br>(0,2)	58<br>(39,7)	7<br>(2,1)	48<br>(40,7)	1<br>(0,7)	0,335615	0,0008	N/A	0,0018[a]<br>0,8847[b]<br>1[c]
**SGLT2 inhibitor**<br>n<br>n (%)	11<br>(5,3)	0<br>(0,0)	5<br>(3,4)	2<br>(0,6)	9<br>(7,6)	0<br>(0,0)	0,31623	0,083928	N/A	N/A

**Oral antidiabetics other than SGLT2 inhibitor and metformin,**<br>n<br>n (%)	26<br>(12,4)	1<br>(0,1)	31<br>(21,2)	3<br>(0,9)	25<br>(21,2)	2<br>(1,4)	0,0436	0,0065	0,1144 [a]<br>0,159 [b]<br>1 [c]	0,1084 [a]<br>0,1001 [b]<br>1 [c]
**Proton pump inhibitor,**<br>n<br>n (%)	27<br>(12,9)	61<br>(5,2)	28<br>(19,2)	42<br>(12,5)	43<br>(36,4)	35<br>(24,0)	<0.0001	<0.0001	0,4372 [a]<br><0.0001 [b]<br>0,00796 [c]	<0.0001 [a]<br><0.0001 [b]<br>0,0071 [c]
**Oral corticosteroid**<br>n<br>n (%)	13<br>(6,2)	48<br>(4,1)	8<br>(5,5)	16<br>(4,7)	4<br>(3,4)	1<br>(0,7)	0,542619	0,093044	N/A	N/A
**Immunosuppression other than oral corticosteroid,**<br>n<br>n (%)	11<br>(5,3)	37<br>(3,1)	5<br>(3,4)	17<br>(5,0)	2<br>(1,7)	0<br>(0,0)	0,263014	0,00661	N/A	0,291 [a]<br>0,0836 [b]<br>0,00753 [c]

Supplementary Table S2. Laboratory parameters measured during the hospitalisation in the diabetic and non-diabetic cohort.

Parameter	Time of assessment	Units	Low risk<br>[0-1]<br>n=209		Medium<br>[2-3]<br>n= 146		High risk<br>[≥4]<br>n=118		**ANOVA**<br>**P value**		**P value for post-hoc analysis in significant ANOVA**<br>**L-M a**<br>**L-H b**<br>**M-H c**	
**Morphology**			Diabetes<br>n=209	Non-Diabetes	Diabetes<br>n= 146	Non-Diabetes	Diabetes<br>n=118	Non-Diabetes	Diabetes	Non-Diabetes	Diabetes	Non-Diabetes
**Leucocytes**<br>n (%)<br>(N)	**On admission**	>12 x10³/µl	n=38<br>18,7<br>(203)	n=159<br>14,8<br>(1073)	n=29<br>20,1<br>(144)	n=53<br>16,2<br>(327)	n=26<br>22,4<br>(116)	n=26<br>18,3<br>(142)				
		4-12 x10³/µl	n=149<br>73,4<br>(203)	n=802<br>74,7<br>(1073)	n=106<br>73,6<br>(144)	n=233<br>71,3<br>(327)	n=81<br>69,8<br>(116)	n=101<br>71,1<br>(142)	0,9091	0,6033	N/A	N/A
		<4 x10³/µl	n=16<br>7,9<br>(203)	n=112<br>10,4<br>(1073)	n=9<br>6,3<br>(144)	n=41<br>12,5<br>(327)	n=9<br>7,8<br>(116)	n=15<br>10,6<br>(142)				
	**On discharge**	>12 x10³/µl	n=44<br>21,7<br>(203)	n=154<br>14,4<br>(1073)	n=37<br>25,7<br>(144)	n=64<br>19,6<br>(327)	n=28<br>24,1<br>(116)	n=35<br>24,6<br>(142)				
		4-12 x10³/µl	n=145<br>71,4<br>(203)	n=849<br>79,1<br>(1073)	n=102<br>70,8<br>(144)	n=228<br>69,7<br>(327)	n=81<br>69,8<br>(116)	n=99<br>39,7<br>(142)	0,6562	0,0003	N/A	0,0043 [a]<br>0,019 [b]<br>0,409 [c]
		<4 x10³/µl	n=14<br>6,9<br>(203)	n=70<br>6,5<br>(1073)	n=5<br>3,5<br>(144)	n=35<br>10,7<br>(327)	n=7<br>6,0<br>(116)	n=8<br>5,6<br>(142)				
**Lymphocytes**<br>n (%)<br>(N)	**On admission**	<1 x10³/µl,	n=70<br>45,8<br>(153)	n=310<br>51,7<br>(600)	n=54<br>51,0<br>(106)	n=118<br>53,2<br>(222)	n=48<br>54,5<br>(88)	n=66<br>62,3<br>(106)				
		1-5 10³/µl	n=80<br>53,2<br>(153)	n=286<br>47,7<br>(600)	n=52<br>49,1<br>(106)	n=100<br>45,0<br>(222)	n=40<br>45,5<br>(88)	n=39<br>36,8<br>(106)	0,3773	0,131	N/A	N/A
		>5 x10³/µl	n=3<br>2,0<br>(153)	n=4<br>0,7<br>(600)	n=0<br>0<br>(106)	n=4<br>1,8<br>(222)	n=0<br>0<br>(88)	n=1<br>0,9<br>(106)				
n (%)<br>(N)	**On discharge**	<1 x10³/µl,	n=39<br>25,5<br>(153)	n=136<br>22,7<br>(600)	n=44<br>41,5<br>(106)	n=67<br>30,2<br>(222)	n=48<br>54,5<br>(88)	n=57<br>53,8<br>(106)				
		1-5 10³/µl	n=212<br>73,2<br>(153)	n=457<br>76,2<br>(600)	n=62<br>58,5<br>(106)	n=150<br>67,6<br>(222)	n=38<br>43,2<br>(88)	n=48<br>45,3<br>(106)	<0.0001	<0.0001	0,0272 [a]<br><0.0001 [b]<br>0,0929 [c]	0,0895 [a]<br><0.0001 [b]<br>0,0003 [c]
		>5 x10³/µl	n=2<br>1,3<br>(153)	n=7<br>1,2<br>(600)	n=0<br>0<br>(106)	n=5<br>2,3<br>(222)	n=2<br>2,3<br>(88)	n=1<br>0,9<br>(106)				
**Neutrophiles**<br>n (%)<br>(N)	**On admission**	<1.5 x10³/µl	n=3<br>2,0<br>(153)	n=18<br>3,0<br>(601)	n=2<br>1,9<br>(107)	n=7<br>3,1<br>(223)	n=3<br>3,4<br>(88)	n=2<br>1,9<br>(106)				
		1.5-8x10³/µl	n=108<br>70,6<br>(153)	n=434<br>72,2<br>(601)	n=67<br>62,6<br>(107)	n=163<br>73,1<br>(223)	n=59<br>67,0<br>(88)	n=76<br>71,7<br>(106)	0,6073	0,973	N/A	N/A
		>8 x10³/µl	n=42<br>27,5<br>(153)	n=149<br>24,8<br>(601)	n=38<br>35,5<br>(107)	n=53<br>23,8<br>(223)	n=26<br>29,5<br>(88)	n=28<br>26,4<br>(106)				
n (%)<br>(N)	**On discharge**	<1.5 x10³/µl	n=5<br>3,3<br>(153)	n=11<br>1,8<br>(601)	n=1<br>0,9<br>(107)	n=6<br>2,7<br>(223)	n=2<br>2,3<br>(88)	n=1<br>0,9<br>(106)	0,3634	0,0281	N/A	1 [a]<br>0,021 [b]<br>0,24 [c]
		1.5-8x10³/µl	n=106<br>69,3	n=460<br>76,5	n=66<br>61,7	n=162<br>72,6	n=55<br>62,5	n=67<br>63,2				

			(153)	(601)	(107)	(223)	(88)	(106)				
		>8 x10³/µl	n=42<br>27,5<br>(153)	n=130<br>21,6<br>(601)	n=40<br>37,4<br>(107)	n=55<br>24,7<br>(223)	n=31<br>35,2<br>(88)	n=38<br>35,8<br>(106)				
**Haemoglobin,**<br>n (%)<br>(N)	**On admission**	<12 g/dl – females,<br><13 g/dl – males<br>(anaemia)	n=74<br>36,5<br>(203)	n=268<br>25,0<br>(1073)	n=66<br>45,8<br>(144)	n=123<br>37,6<br>(327)	n=79<br>68,1<br>(116)	n=64<br>45,1<br>(142)	<0.0001	<0.0001	0,3006 [a]<br><0.0001 [b]<br>0,0016 [c]	<0.0001 [a]<br><0.0001 [b]<br>0,473 [c]
		≥12 g/dl F<br>≥13 g/dl M	n=129<br>63,5<br>(203)	n=805<br>75,0<br>(1073)	n=78<br>54,2<br>(144)	n=204<br>62,4<br>(327)	n=37<br>31,9<br>(116)	n=78<br>54,9<br>(142)				
n (%)<br>(N)	**On discharge**	<12 g/dl – females,<br><13 g/dl – males<br>(anaemia)	n=110<br>54,2<br>(203)	n=387<br>36,1<br>(1073)	n=86<br>59,7<br>(144)	n=166<br>50,8<br>(327)	n=90<br>77,6<br>(116)	n=76<br>53,5<br>(142)	0,00016	<0.0001	1 [a]<br>0,0002 [b]<br>0,01 [c]	<0.0001 [a]<br>0,0003 [b]<br>1,0 [c]
		≥12 g/dl F<br>≥13 g/dl M	n=93<br>45,8<br>(203)	n=686<br>63,9<br>(1073)	n=58<br>40,8<br>(144)	n=161<br>49,2<br>(327)	n=26<br>22,4<br>(116)	n=66<br>46,5<br>(142)				
**Reticulocytes**<br>mean±SD<br>min-max<br>(N)	**On admission**	x 10⁹/L	56,8±34,4<br>4<br>8,1-137,6<br>(19)	60,4±33,69<br>5,3-132,7<br>(84)	57,0±27,79<br>19,0-109,7<br>(23)	55,0±38,39<br>10,1-224,4<br>(40)	61,2±40,46<br>4,5-142,3<br>(14)	58,2±32,4<br>16,4-123,3<br>(14)	0,937	0,753	N/A	N/A
mean±SD<br>min-max<br>(N)	**On discharge**	x 10⁹/L	61,0±37,9<br>3<br>8,1-137,6<br>(203)	61,4±32,63<br>4,9-132,7<br>(84)	59,3±41,47<br>19,0-216,4<br>(23)	57,9±40,61<br>7-230,3<br>(40)	53,2±37,73<br>9,1-162,4<br>(14)	58,7±36,39<br>12,9-123,3<br>(14)	0,837	0,8817	N/A	N/A
**Platelets**<br>mean±SD<br>min-max<br>(N)	**On admission**		243,6±10<br>0,91<br>0-565,0<br>(203)	234,6±110,7<br>1<br>4,0-1356,0<br>(1073)	242,5±116,1<br>0<br>53,0-730,0<br>(144)	224,7±112,3<br>5<br>3,0-740,0<br>(327)	216,2±88,74<br>8,0-578,0<br>(116)	215,6±96,6<br>4<br>15,0-537,0<br>(142)	0,028	0,0586	N/A	0,995 [a]<br>0,032 [b]<br>0,098 [c]
n (%)<br>(N)		>400 x10³ /µl	n=15<br>7,4<br>(203)	n=72<br>6,7<br>(1073)	n=12<br>8,3<br>(144)	n=22<br>6,7<br>(327)	n=4<br>3,4<br>(116)	n=6<br>4,2<br>(142)	0,225	0,0969	N/A	N/A
		150-400 x10³ / µl	n=157<br>77,3<br>(203)	n=815<br>76,0<br>(1073)	n=104<br>72,2<br>(144)	n=234<br>71,6<br>(327)	n=86<br>74,1<br>(116)	n=102<br>71,8<br>(142)				
		100- 150 x10³ /µl	n=24<br>11,8<br>(203)	n=145<br>13,5<br>(1073)	n=20<br>13,9<br>(144)	n=48<br>14,7<br>(327)	n=20<br>17,2<br>(116)	n=20<br>14,1<br>(142)				
		<100 x10³ /µl	n=7<br>3,4<br>(203)	n=6<br>0,6<br>(1073)	n=8<br>5,6<br>(144)	n=6<br>1,8<br>(327)	n=6<br>5,2<br>(116)	n=3<br>2,1<br>(142)				
mean±SD<br>min-max<br>(N)	**On discharge**		267,9±13<br>2,63<br>6,0-691,0<br>(203)	272,3±128,4<br>4<br>2,0-1101,0<br>(1071)	252,1±116,1<br>15,0-606,0<br>(144)	241,9±122,9<br>3,0-694,0<br>(327)	211,4±98,04<br>4,0-592,0<br>(116)	213,1±99,5<br>1<br>15,0-521,0<br>(142)	<0.0001	<0.0001	0,465 [a]<br><0.0001 [b]<br>0,007 [c]	0,0004 [a]<br><0.0001 [b]<br>0,021 [c]
n (%)<br>(N)		>400 x10³ /µl	n=36<br>17,7<br>(203)	n=159<br>14,8<br>(1071)	n=15<br>10,4<br>(144)	n=34<br>10,4<br>(327)	n=5<br>4,3<br>(116)	n=5<br>3,5<br>(142)	0,912	<0.0001	N/A	0,0076 [a]<br><0.0001 [b]<br>0,2898 [c]
		150-400 x10³ / µl	n=133<br>65,5<br>(203)	n=768<br>71,7<br>(1071)	n=103<br>71,5<br>(144)	n=226<br>69,1<br>(327)	n=81<br>69,8<br>(116)	n=96<br>67,6<br>(142)				
		100-150 x10³ /µl	n=12<br>5,9<br>(203)	n=102<br>9,5<br>(1071)	n=14<br>9,7<br>(144)	n=36<br>11,0<br>(327)	n=18<br>15,5<br>(116)	n=24<br>16,9<br>(142)				
		<100 x10³ /µl	n=22<br>10,8<br>(203)	n=16<br>1,5<br>(1071)	n=12<br>8,3<br>(144)	n=10<br>3,1<br>(327)	n=12<br>10,3<br>(116)	n=5<br>3,5<br>(142)				
**Acid -base balance in the arterial blood gas**												
**PH**<br>mean±SD<br>min-max<br>(N)	**On admission**		7,42±0,09<br>7,19-7,58<br>(37)	7,43±0,08<br>7,04-7,57<br>(83)	7,42±0,06<br>7,29-7,53<br>(32)	7,43±0,07<br>7,10-7,54<br>(56)	7,39±0,09<br>7,09-7,52<br>(30)	7,43±0,06<br>7,26-7,54<br>(35)	0,293	0,866	N/A	N/A
n (%)<br>(N)		<7.35,<br>(acidosis)	n=6<br>16,2<br>(37)	n=8<br>9,6<br>(83)	n=3<br>9,4<br>(32)	n=4<br>7,1<br>(56)	n=7<br>23,3<br>(30)	n=3<br>8,6<br>(35)	0,332	0,9377	N/A	N/A
		≥7.35	n=31<br>83,8<br>(37)	n=75<br>90,4<br>(83)	n=29<br>90,6<br>(32)	n=52<br>92,9<br>(56)	n=23<br>76,7<br>(30)	n=32<br>91,4<br>(35)				
mean±SD<br>min-max<br>(N)	**On discharge**		7,42±0,08<br>7,16-7,54<br>(37)	7,42±0,08<br>7,06-7,54<br>(83)	7,42±0,06<br>7,30-7,54<br>(32)	7,43±0,08<br>7,01-7,55<br>(56)	7,42±0,06<br>7,33-7,56<br>(30)	7,42±0,06<br>7,25-7,52<br>(35)	0,979	0,8499	N/A	N/A
n (%)<br>(N)		<7.35,<br>(acidosis)	n=4<br>10,8<br>(37)	n=8<br>9,6<br>(83)	n=5<br>15,6<br>(32)	n=4<br>7,1<br>(56)	n=1<br>13,3<br>(30)	n=3<br>8,6<br>(35)	0.597	0,9377	N/A	N/A
		≥7.35	n=33<br>89,2<br>(37)	n=75<br>90,4<br>(83)	n=27<br>84,4<br>(32)	n=52<br>92,9<br>(56)	n=29<br>96,7<br>(30)	n=32<br>91,4<br>(35)			N/A	N/A
**PaO2**<br>mean±SD<br>min-max<br>(N)	**On admission**		70,8±33,2<br>0<br>23,5-<br>207,0	73,1±24,51<br>12,8-156,0<br>(83)	79,4±55,62<br>28,3-298,0<br>(32)	74,5±42,85<br>28,6-286,0<br>(56)	66,8±36,24<br>23,7-222,0<br>(30)	72,5±33,74<br>32,8-211,0<br>(35)	0,574	0,965	N/A	N/A

			(37)									
n (%)<br>(N)		<60 mmHg, (respiratory insufficiency)	n=13<br>35,1<br>(37)	n=24<br>28,9<br>(83)	n=14<br>43,8<br>(32)	n=19<br>33,9<br>(56)	n=16<br>53,3<br>(30)	n=14<br>40,0<br>(35)	0,327	0,491	N/A	N/A
		≥60 mmHg	n=24<br>64,9<br>(37)	n=59<br>71,1<br>(83)	n=18<br>56,3<br>(32)	n=37<br>66,1<br>(56)	n=14<br>46,7<br>(30)	n=21<br>60,0<br>(35)				
mean±SD<br>min-max<br>(N)	**On discharge**		77,0±30,21<br>23,5-207,0<br>(37)	74,8±25,02<br>12,8-165,0<br>(83)	84,9±58,08<br>23,3-298,0<br>(32)	73,5±42,15<br>28,6-286,0<br>(56)	61,7±19,56<br>31,8-114,0<br>(30)	70,8±33,20<br>23,5-207,0<br>(35)	0,015	0,5447	0,771 [a]<br>0,04 [b]<br>0,097 [c]	N/A
n (%)<br>(N)		<60 mmHg, (respiratory insufficiency)	n=8<br>21,6<br>(37)	n=20<br>24,1<br>(83)	n=12<br>37,5<br>(32)	n=18<br>32,1<br>(56)	n=18<br>60,0<br>(30)	n=14<br>40,0<br>(35)	0,0057	0,20469	0,7096 [a]<br>0,0094 [b]<br>0,3875 [c]	N/A
		≥60 mmHg	n=29<br>78,4<br>(37)	n=63<br>75,9<br>(83)	n=20<br>62,5<br>(32)	n=38<br>67,9<br>(56)	n=12<br>40,0<br>(30)	n=21<br>60,0<br>(35)				
**PaCO2**<br>mean±SD<br>min-max<br>(N)	**On admission**		36,7±9,80<br>20,2-61,4<br>(37)	38,7±10,51<br>25,7-82,4<br>(83)	36,4±8,67<br>20,9-67,0<br>(32)	36,8±9,89<br>25,4-79,4<br>(56)	38,2±10,13<br>25,7-74,9<br>(30)	38,9±11,99<br>19,7-88,4<br>(35)	0,741	0,5124	N/A	N/A
n (%)<br>(N)		≥ 45 mmHg, (hypercapnia)	n=7<br>18,9<br>(37)	n=16<br>19,3<br>(83)	n=4<br>12,5<br>(32)	n=6<br>10,7<br>(56)	n=6<br>20,0<br>(30)	n=6<br>17,1<br>(35)	0,691	0,3962	N/A	N/A
		< 45 mmHg	n=30<br>81,1<br>(37)	n=67<br>80,7<br>(83)	n=28<br>87,5<br>(32)	n=50<br>89,3<br>(56)	n=24<br>80,0<br>(30)	n=29<br>82,9<br>(35)				
mean±SD<br>min-max<br>(N)	**On discharge**		36,2±9,64<br>20,2-59,4<br>(37)	39,5±9,81<br>26,8-75,5<br>(83)	37,5±7,63<br>22,4-59,5<br>(32)	38,2±12,53<br>20,9-88,4<br>(56)	37,2±6,20<br>25,7-50,6<br>(30)	40,0±12,76<br>25,0-88,4<br>(35)	0,816	0,7567	N/A	N/A
n (%)<br>(N)		≥ 45 mmHg, (hypercapnia)	n=6<br>16,2<br>(37)	n=14<br>16,9<br>(83)	n=4<br>12,5<br>(32)	n=7<br>12,5<br>(56)	n=3<br>10,0<br>(30)	n=7<br>20,0<br>(35)	0,814	0,6163	N/A	N/A
		< 45 mmHg	n=31<br>83,8<br>(37)	n=69<br>83,1<br>(83)	n=28<br>87,5<br>(32)	n=49<br>87,5<br>(56)	n=27<br>90,0<br>(30)	n=28<br>80,0<br>(35)				
**PaO2 and PaCO2**<br>n (%)<br>(N)	**On admission**	PaO2 <60 mmHg and PaCO2 ≥ 45 mmHg, (complete respiratory insufficiency)	n=3<br>8,1<br>(37)	n=4<br>4,8<br>(83)	n=3<br>9,4<br>(32)	n=3<br>5,4<br>(56)	n=5<br>16,7<br>(30)	n=4<br>11,4<br>(35)	0,525	0,417	N/A	N/A
		PaO2 ≥60 mmHg and PaCO2 <45 mmHg	n=34<br>91,9<br>(37)	n=79<br>95,2<br>(83)	n=29<br>90,6<br>(32)	n=53<br>94,6<br>(56)	n=25<br>83,3<br>(30)	n=31<br>88,6<br>(35)				
n (%)<br>(N)	**On discharge**	PaO2 <60 mmHg and PaCO2 ≥ 45 mmHg, (complete respiratory insufficiency)	n=1<br>2,7<br>(37)	n=3<br>3,6<br>(83)	n=1<br>3,1<br>(32)	n=3<br>5,4<br>(56)	n=2<br>6,7<br>(30)	n=4<br>11,4<br>(35)	0,684	0.2473	N/A	N/A
		PaO2 ≥60 mmHg and PaCO2 <45 mmHg	n=36<br>97,3<br>(37)	n=80<br>96,4<br>(83)	n=31<br>96,9<br>(32)	n=53<br>94,6<br>(56)	n=28<br>93,3<br>(30)	n=31<br>88,6<br>(35)				
**HCO3 standard**<br>mean±SD<br>min-max<br>(N)	**On admission**	mmol/l	24,0±4,82<br>12,1-32,9<br>(37)	25,3±3,15<br>15,6-32,7<br>(82)	23,8±4,07<br>14,3-32,7<br>(32)	24,7±4,26<br>15,5-39,5<br>(53)	22,6±3,72<br>13,5-30,7<br>(30)	25,4±4,94<br>17,5-38,6<br>(35)	0,301	0,6971	N/A	N/A
mean±SD<br>min-max<br>(N)	**On discharge**		23,9±4,28<br>12,1-31,2<br>(37)	25,4±3,51<br>13,1-35,7<br>(82)	24,4±4,14<br>13,7-33,9<br>(32)	25,8±6,27<br>14,0-51,7<br>(53)	24,2±3,54<br>17,4-30,7<br>(30)	25,4±4,36<br>18,5-36,7<br>(35)	0,895	0,89765	N/A	N/A
**BE**<br>mean±SD<br>min-max<br>(N)	**On admission**		[-]1,0±5,97<br>[-]15,7-10,5<br>(15)	2,1±3,64<br>[-]8,4-7,9<br>(25)	[-]0,1±5,66<br>[-]12,5-9,0<br>(19)	3,1±4,62<br>[-]4,3-15,7<br>(24)	1,0±3,37<br>[-] 3,3-7,0<br>(12)	3,2±7,15<br>[-]7,4-14,6<br>(10)	0,533	0,64847	N/A	N/A

mean±SD<br>min-max<br>(N)	**On discharge**		[-]1,4±6,09<br>[-]15,7-6,0<br>(15)	2,0±4,80<br>[-]11,0-11,9<br>(25)	0,8±5,51<br>[-]13,6-10,1<br>(19)	3,6±6,23<br>[-]14,7-17,1<br>(24)	0,6±4,14<br>[-] 5,3-7,0<br>(12)	2,1±5,96<br>[-]7,4-13,2<br>(10)	0,509	0,5946	N/A	N/A
**Lactates**<br>mean±SD<br>min-max<br>(N)	**On admission**		2,8±2,59<br>0,8-12,8<br>(31)	2,3±0,89<br>0,6-4,9<br>(73)	1,9±0,77<br>0,5-3,7<br>(28)	2,07±0,91<br>0,6-5,7<br>(51)	2,8±2,31<br>0,6-12,0<br>(27)	2,3±1,31<br>0,8-6,0<br>(32)	0,047	0,3344	0,15 [a]<br>0,998 [b]<br>0,155 [c]	N/A
mean±SD<br>min-max<br>(N)	**On discharge**		2,7±2,58<br>0,9-12,8<br>(31)	2,4±0,89<br>0,7-5,1<br>(73)	1,9±0,81<br>0,5-4,2<br>(28)	2,2±1,13<br>0,6-6,4<br>(51)	2,5±1,10<br>0,8-4,5<br>(27)	2,4±1,32<br>0,8-6,0<br>(32)	0,072	0,5348	N/A	N/A
**Lactate acidosis**<br>n (%)<br>(N)		pH <7.35 and lactates >1.6 mmol/L	n=3<br>9,7<br>(31)	n=6<br>8,2<br>(73)	n=2<br>7,1<br>(28)	n=3<br>5,9<br>(51)	n=6<br>22,2<br>(27)	n=2<br>6,25<br>(32)	0,258	0,9199	N/A	N/A
		pH >7.35 or and lactates < 1.6 mmol/L	n=28<br>90,3<br>(31)	n=67<br>91,8<br>(73)	n=26<br>92,9<br>(28)	n=48<br>94,1<br>(51)	n=21<br>77,8<br>(27)	n=30<br>93,75<br>(32)				
**Osmolarity**<br>mean±SD<br>min-max<br>(N)	**On admission**	mOsm	310,3±17,14<br>282-370<br>(29)	301,5±16,24<br>247,0-362,0<br>(94)	319,6±24,7<br>292-373<br>(11)	301,2±25,95<br>233,0-357,0<br>(30)	311,6±18,13<br>277,5-344,5<br>(14)	298,8±25,8<br>245,8-343,0<br>(18)	0,542	0,9174	N/A	N/A
mean±SD<br>min-max<br>(N)	**On discharge**		306,6±19,79<br>270-348<br>(29)	298,0±15,35<br>272,0-362,0<br>(94)	314,2±28,51<br>281,4-373<br>(11)	300,5±19,33<br>263,0-356,0<br>(30)	311,1±20,39<br>287,1-346,6<br>(14)	299,8±24,49<br>265,0-353,0<br>(18)	0,642	0,8035	N/A	N/A
**Electrolytes, inflammatory and iron biomarkers**												
**Na**<br>mean±SD<br>min-max<br>(N)	**On admission**	mmol/l	137,4±4,65<br>121-150<br>(200)	138,4±4,31<br>106,0-159,0<br>(1064)	137,9±6,36<br>106-157<br>(141)	137,7±7,29<br>101,0-175,0<br>(325)	137,7±6,37<br>124-174<br>(116)	138,0±7,24<br>108,0-163,0<br>(142)	0,767	0,2376	N/A	N/A
mean±SD<br>min-max<br>(N)	**On discharge**		137,4±4,7<br>121-150<br>(200)	139,0±4,11<br>109,0-167,0<br>(1064)	137,9±6,6<br>106-157<br>(141)	138,8±7,12<br>101,0-172,0<br>(325)	137,7±6,37<br>124-172<br>(116)	140,3±6,95<br>124,0-170,0<br>(142)	0,395	0,0689	N/A	N/A
**K**<br>mean±SD<br>min-max<br>(N)	**On admission**	mmol/l	4,2±0,74<br>2,5-7,5<br>(202)	4,0±0,54<br>2,0-6,5<br>(1067)	4,3±0,77<br>2,42±6,8<br>(142)	4,1±0,66<br>2,4-7,0<br>(326)	4,5±0,85<br>2,6-8,7<br>(116)	4,1±0,75<br>2,5-7,4<br>(142)	0,029	0,1221	0,72 [a]<br>0,022 [b]<br>0,155 [c]	N/A
mean±SD<br>min-max<br>(N)	**On discharge**		4,4±0,7<br>2,5-7,4<br>(202)	4,2±0,56<br>2,0-6,3<br>(1067)	4.4±0,82<br>2,3-6,9<br>(142)	4,3±0,73<br>2,4-7,0<br>(326)	4,5±0,69<br>2,8-6,6<br>(116)	4,4±0,69<br>2,53-6,43<br>(142)	0,319	0,0007	N/A	0,028 [a]<br>0,006 [b]<br>0,533 [c]
**Mg**<br>mean±SD<br>min-max<br>(N)	**On admission**	mg/dl	2,0±0,38<br>0,85-2,9<br>(163)	2,1±0,34<br>0,95-5,24<br>(744)	2,0±0,43<br>1,3-3,4<br>(105)	2,1±0,45<br>1,26-4,1<br>(232)	2,0±0,43<br>1,1-3,4<br>(96)	2,1±0,38<br>1,28-4,02<br>(106)	0,774	0,7404	N/A	N/A
mean±SD<br>min-max<br>(N)	**On discharge**		2,0±0,39<br>0,8-3,4<br>(163)	2,1±0,31<br>0,9-5,24<br>(744)	2,0±0,53<br>1,1-4,2<br>(105)	2,1±0,39<br>1,16-4,1<br>(232)	1,9±0,35<br>1,3-2,8<br>(96)	2,1±0,44<br>1,01-4,02<br>(106)	0,621	0,7510	N/A	N/A
**CRP**<br>mean±SD<br>min-max<br>(N)	**On admission**	mg/l	96,2±88,25<br>0,39-531,6<br>(203)	73,4±83,2<br>0,13-496,98<br>(1049)	89,5±101,94<br>0,75-487,4<br>(143)	80,8±79,92<br>0,29-538,55<br>(325)	76,6±81,61<br>0,4-345<br>(116)	78,7±0,4-<br>390,9<br>(142)	0,136	0,3103	N/A	N/A
mean±SD<br>min-max<br>(N)	**On discharge**		68,1±98,39<br>0,5-494,7<br>(203)	44,8±74,68<br>0,13-496,98<br>(1049)	80,0±97,25<br>0,22-432,9<br>(143)	67,9±91,47<br>0,25-538,55<br>(325)	68,4±75,45<br>0,42-289,5<br>(116)	81,1±96,86<br>0,4-431,9<br>(142)	0,465	<0.0001	N/A	0,00012 [a]<br><0.0001 [b]<br>0,358 [c]
**Procalcitonin**<br>mean±SD<br>min-max<br>(N)	**On admission**	ng/ml	1,5±6,28<br>0,01-61,28<br>(163)	0,72±4,03<br>0,01-55,01<br>(736)	2,1±9,56<br>0.02-72,61<br>(111)	1,73±13,2<br>0,01-196,04<br>(228)	1,0±2,64<br>0,01-17,48<br>(97)	2,0±8,21<br>0,01-60,77<br>(106)	0,399	0,1596	N/A	N/A
mean±SD<br>min-max<br>(N)	**On discharge**		1,9±8,92<br>0,01-75,16<br>(163)	0,70±3,80<br>0,01-44,39<br>(736)	2,36±9,01<br>0,01-81,09<br>(111)	1,10±3,88<br>0,01-30,67<br>(228)	1,1±3,62<br>0,01-27,61<br>(97)	1,27±6,13<br>0,02-60,77<br>(106)	0,323	0,2963	N/A	N/A
**IL-6 !!!**<br>mean±SD<br>min-max<br>(N)	**On admission**	pg/ml	52,3±128,18<br>2-1000<br>(74)	64,1±463,3<br>2,0-9099,0<br>(397)	43,2±67,97<br>2,33-398<br>(34)	42,8±62,71<br>2,0-499,0<br>(108)	65,0±94,78<br>2,51-421<br>(36)	63,2±98,39<br>2,0-373,0<br>(43)	0,541	0,3417	N/A	N/A
mean±SD<br>min-max<br>(N)	**On discharge**		39,9±120,82<br>2-1000<br>(74)	66,4±473,84<br>2,0-9099,0<br>(397)	36,9±69,08<br>2-398<br>(34)	40,6±76,18<br>2,0-449,0<br>(108)	99,2±217,1<br>2,51-1000<br>(36)	57,7±83,59<br>2,0-369,0<br>(43)	0,268	0,3484	N/A	N/A
**ESR**<br>mean±SD<br>min-max<br>(N)	**On admission**	mm/h	48,6±40,77<br>5-139<br>(12)	37,2±32,41<br>2,0-137,0<br>(52)	41,4±31,29<br>2-100<br>(15)	23,3±18,58<br>1,0-78,0 (24)	50,1±36,44<br>5-128<br>(15)	34,8±29,20<br>3,0-102,0<br>(11)	0,762	0,0733	N/A	N/A
mean±SD<br>min-max<br>(N)	**On discharge**		44,5±37,80<br>5-139<br>(12)	33,0±29,33<br>1,0-122,0<br>(52)	42,3±33,51<br>2-100<br>(15)	24,5±18,64<br>1,0-78,0<br>(24)	50±36,13<br>5-126<br>(15)	38,1±24,09<br>7,0-75,0<br>(11)	0,832	0,1690	N/A	N/A

**D-dimer**<br>mean±SD<br>min-max<br>(N)	**On admission**	μg/ml	3,0±6,94<br>0,21-69,6<br>(163)	3,9±13,05<br>0,15-132,8<br>(820)	7,6±20,65<br>0.2-127.2<br>(105)	6,2±15,17<br>0,23-107,7<br>(261)	3,9±14,18<br>0.22-128.0<br>(88)	6,1±18,32<br>0,24-128,0<br>(109)	0,085	0,05811	N/A	N/A
mean±SD<br>min-max<br>(N)	**On discharge**		2,4±4,43<br>0,21-32,37<br>(163)	3,4±11,68<br>0,15-128,0<br>(820)	6,7±16,8<br>0,21-106,02<br>(105)	5,3±11,78<br>0,22-89,96<br>(261)	3,1±5,21<br>0,22-27,91<br>(88)	3,9±10,99<br>0,21-107,54<br>(109)	0,027	0,0844	N/A	N/A
**INR**<br>mean±SD<br>min-max<br>(N)	**On admission**		1,2±0,30<br>0,88-3,6<br>(196)	1,1±0,51<br>0,82-15,2<br>(1008)	1,3±0,53<br>0,87-5,58<br>(133)	1,3±0,62<br>0,88-7,8<br>(303)	2,0±3,02<br>0,9-21,1<br>(111)	1,6±1,91<br>0,89-18,74<br>(131)	0,007	0,0002	0,301 [a]<br>0,013 [b]<br>0,029 [c]	0,005 [a]<br>0,012 [b]<br>0,087 [c]
n (%)<br>(N)		>1.5	n=9<br>4,6<br>(196)	n=32<br>3,2<br>(1008)	n=13<br>9,8<br>(133)	n=31<br>10,2<br>(303)	n=21<br>18,9<br>(111)	n=32<br>24,4<br>(131)	0,0003	<0.0001	0,3145 [a]<br>0,0003 [b]<br>0,185 [c]	<0.0001<br><0.0001<br>0,0006 [c]
		≤1.5	n=187<br>95,4<br>(196)	n=976<br>86,8<br>(1008)	n=120<br>90,2<br>(133)	n=272<br>89,8<br>(303)	n=90<br>91,1<br>(111)	n=99<br>75,6<br>(131)				
mean±SD<br>min-max<br>(N)	**On discharge**		1,2±0,63<br>0,88-9,18<br>(196)	1,1±0,32<br>0,82-6,82<br>(1008)	1,3±0,49<br>0,88-6,0<br>(133)	1,3±0,85<br>0,88-13,09<br>(303)	1,6±2,0<br>0,93-21,1<br>(111)	1,3±0,75<br>0,87-7,99<br>(131)	0,170	0,000103	N/A	0,011 [a]<br>0,003 [b]<br>0,609 [c]
n (%)<br>(N)		>1.5	n=15<br>7,7<br>(196)	n=33<br>3,3<br>(1008)	n=12<br>9,0<br>(133)	n=25<br>8,3<br>(303)	n=20<br>18,0<br>(111)	n=22<br>16,8<br>(131)	0,014	<0.0001	1 [a]<br>0,0315 [b]<br>0,1793 [c]	0,0012 [a]<br><0.0001<br>0,0416 [c]
		≤1.5	n=181<br>92,3<br>(196)	n=975<br>96,7<br>(1008)	n=121<br>91,0<br>(133)	n=278<br>91,7<br>(303)	n=91<br>82,0<br>(111)	n=109<br>83,2<br>(131)				
**aPTT**,<br>n (%)<br>(N)	**On admission**	>60 s	n=5<br>2,6<br>(189)	n=22<br>2,2<br>(979)	n=1<br>0,8<br>(128)	n=6<br>2,0<br>(294)	n=9<br>8,3<br>(109)	n=2<br>1,6<br>(126)	0,008	0,9999	1 [a]<br>0,1296 [b]<br>0,0192 [c]	N/A
		≤60 s	n=184<br>97,4<br>(189)	n=957<br>97,8<br>(979)	n=127<br>99,2<br>(128)	n=288<br>98,0<br>(294)	n=100<br>91,7<br>(109)	n=124<br>98,4<br>(126)				
n (%)<br>(N)	**On discharge**	>60 s	n=13<br>6,9<br>(189)	n=33<br>3,4<br>(979)	n=1<br>0,8<br>(128)	n=6<br>2,0<br>(294)	n=11<br>10,1<br>(109)	n=1<br>0,8<br>(126)	0,007	0,2189	0,062 [a]<br>1 [b]<br>0,0092 [c]	N/A
		<60 s	n=176<br>93,1<br>(189)	n=946<br>96,6<br>(979)	n=127<br>99,2<br>(128)	n=288<br>98,0<br>(294)	n=98<br>89,9<br>(109)	n=125<br>99,2<br>(126)				
**Fibrinogen**<br>mean±SD<br>min-max<br>(N)	**On admission**	g/dl	4,8±1,88<br>0,35-8,75<br>(46)	4,9±1,82<br>0,44-10,0<br>(228)	5,4±1,88<br>1,74-9,04<br>(21)	4,6±1,67<br>0,37-9,2<br>(55)	4,6±1,73<br>1,78-9,1<br>(29)	4,4±1,62<br>2,1-8,11<br>(24)	0,364	0,3363	N/A	N/A
mean±SD<br>min-max<br>(N)	**On discharge**		5,1±2,27<br>1,76-10,0<br>(46)	4,7±1,92<br>0,44-10,0<br>(228)	5,6±2,23<br>1,52-10,0<br>(21)	4,7±2,01<br>0,37-9,2<br>(55)	4,9±1,83<br>1,53-8,71<br>(29)	4,9±2,13<br>2,1-9,04<br>(24)	0,477	0,8916	N/A	N/A
**Ferritin**<br>mean±SD<br>min-max<br>(N)	**On admission**	ng/ml	1047,7±1064,24<br>55,3-5430<br>(113)	952,7±1863,83<br>9,4-37400,0<br>(502)	745,1±917,14<br>9,0-6226<br>(66)	833,9±992,64<br>8,0-7316,0<br>(149)	672,8±909,67<br>15,7-5910<br>(57)	894,8±823,99<br>19,0-3560,0<br>(67)	0,035	0,5949	0,114 [a]<br>0,047 [b]<br>0,9 [c]	N/A
mean±SD<br>min-max<br>(N)	**On discharge**		961,6±976,85<br>55,3-5430<br>(113)	912,5±1980,9<br>9,4-3740,0<br>(502)	777,2±962,56<br>9,0-6226<br>(66)	822,8±1067,7<br>8,0-7970,0<br>(149)	3408,6±15286,5<br>22,4-103000<br>(57)	974,0±921,85<br>17,0-4602,0<br>(67)	0,224	0,5481	N/A	N/A
**TSAT**<br>(Fe/TIBC)<br>mean±SD<br>min-max<br>(N)	**On admission**	%	22,4±12,23<br>6,09-45,18<br>(18)	26,4±26,10<br>2,38-200,0<br>(68)	19,2±13,72<br>3,51-58,52<br>(25)	22,0±17,65<br>0-68,29<br>(25)	17,0±13,09<br>0-44,17<br>(17)	28,8±15,23<br>6,88-61,74<br>(17)	0,462	0,4142	N/A	N/A
mean±SD<br>min-max<br>(N)	**On discharge**		24,3±12,37<br>4,0-45,18<br>(18)	24,8±16,93<br>3,1-86,94<br>(68)	20,5±14,30<br>3,51-59,26<br>(25)	23,0±18,03<br>0-68,29 (25)	17,2±14,08<br>0-48,33<br>(17)	24,6±12,98<br>6,89-50,22<br>(17)	0,301	0,9133	N/A	N/A
**Fe**<br>mean±SD<br>min-max<br>(N)	**On admission**		56,2±33,52<br>14,0-151,9<br>(31)	55,9±41,87<br>10,0-178,0<br>(94)	45,3±43,94<br>10,0-197,0<br>(35)	47,7±34,31<br>10,0-155,25<br>(42)	37,6±23,54<br>10,0-101,0<br>(25)	58,7±54,13<br>16,2-314,0<br>(32)	0,061	0,4197	N/A	N/A
mean±SD<br>min-max<br>(N)	**On discharge**		59,8±35,77<br>10,0-151,9<br>(31)	58,6±51,33<br>10,0-366,6<br>(94)	41,3±36,14<br>10,0-197,0<br>(35)	52,0±39,16<br>10,0-169,8<br>(42)	39,0±27,46<br>5,0-107,78<br>(25)	56,7±55,11<br>11,2-320,0<br>(32)	0,041	0,7159	0,099 [a]<br>0,044 [b]<br>0,959 [c]	N/A
**sTfR**<br>mean±SD<br>min-max<br>(N)	**On admission**	mg/l	1,2±0,37<br>0,8-1,81<br>(9)	2,1±1,46<br>0,4-5,21<br>(8)	1,9±0,83<br>1,02-2,94<br>(6)	1,3±0,41<br>0,79-2,3<br>(14)	2,1±1,03<br>1,02-4,01<br>(6)	1,4±0,65<br>0,94-2,71<br>(6)	0,093	0,3524	N/A	N/A
mean±SD<br>min-max<br>(N)	**On discharge**		1,2±0,37<br>0,8-1,81<br>(9)	2,1±1,46<br>0,4-5,21<br>(8)	1,8±0,73<br>1,18-2,94<br>(6)	1,3±0,41<br>0,79-2,3<br>(14)	2,1±1,03<br>1,02-4,01<br>(6)	1,4±0,65<br>0,94-2,71<br>(6)	0,089	0,3548	N/A	N/A
**Vitamin B12**<br>mean±SD<br>min-max<br>(N)	**On admission**	pg/ml	507,5±322,13<br>131,0-1171,0<br>(34)	849,7±1266,1<br>150,0-6000,0<br>(89)	459,0±307,13<br>115,0-1500,0<br>(29)	624,5±919,3<br>109,0-5907,0<br>(52)	556,5±501,86<br>109,0-2659,0<br>(37)	613,7±773,5<br>155,0-4344,0<br>(29)	0,612	0,3831	N/A	N/A
mean±SD<br>min-max<br>(N)	**On discharge**		495,2±311,33	793,2±1061,9<br>147,0-6000,0	520,1±308,41<br>128,0-1500,0	703,2±928,8<br>109,0-5907,0<br>(52)	611,2±481,85<br>109,0-2659,0	624,3±768,91	0,478	0,6449	N/A	N/A

			131,0-<br>1171,0<br>(34)	(89)	(29)		(37)	155,0-<br>4344,0<br>(29)				
**Folic acid**<br>mean±SD<br>min-max<br>(N)	**On admission**	ng/ml	7,3±5,46<br>2,18-24,0<br>(29)	7,3±4,41<br>1,5-20,0<br>(92)	8,6±5,11<br>1,8-20,0<br>(30)	7,1±5,43<br>2,6-24,0<br>(49)	7,5±5,29<br>1,6-24,0<br>(38)	8,1±7,04<br>1,0-24,0<br>(31)	0,590	0,8009	N/A	N/A
mean±SD<br>min-max<br>(N)	**On discharge**		6,7±4,68<br>2,18-24,0<br>(29)	7,6±4,59<br>1,6-24,0<br>(92)	9,3±5,21<br>2,5-20,0<br>(30)	7,8±5,95<br>1,4-24,0<br>(49)	8,7±6,18<br>1,6-24,0<br>(38)	9,2±7,44<br>1,0-24,0<br>(31)	0,102	0,5253	N/A	N/A
	**Biochemistry**											
**Glucose**<br>mean±SD<br>min-max<br>(N)	**On admission**	mg/dl	207,2±12<br>0,86<br>28-733<br>(186)	119,1±48,6<br>50,0-933,0<br>(864)	208,9±136,2<br>47-1026<br>(132)	126,0±45,14<br>61,0-396,0<br>(309)	189,3±134,8<br>37-1064<br>(108)	119,7±34,1<br>49,0-292,0<br>(129)	0,455	0,0725	N/A	N/A
mean±SD<br>min-max<br>(N)	**On discharge**		173,3±92,<br>95<br>44-637<br>(186)	113,3±60,22<br>37,0-1444,0<br>(864)	187,1±113,2<br>47-596<br>(132)	123,0±55,15<br>59,0-484,0<br>(309)	174,8±97,68<br>14-685<br>(108)	120,4±45,3<br>49,0-341,0<br>(129)	0,495	0,0225	N/A	0,026 [a]<br>0,249 [b]<br>0,871 [c]
**Glycated hemoglobin (HbA1c)**<br>mean±SD<br>min-max<br>(N)	**On admission**	%	8,6±2,26<br>5,7-14,9<br>(65)	6,2±1,37<br>4,2-11,2<br>(58)	8,3±2,37<br>5,6-16,6<br>(47)	6,3±0,92<br>4,8-8,5<br>(27)	7,5±1,62<br>5,1-11,9<br>(42)	6,8±2,24<br>5,1-13,7<br>(15)	0,002	0,6869	0,438 [a]<br>0,002 [b]<br>0,163 [c]	N/A
mean±SD<br>min-max<br>(N)	**On discharge**		8,7±2,20<br>5,7-14,9<br>(65)	6,2±1,37<br>4,2-11,2<br>(58)	8,2±2,40<br>5,6-16,8<br>(47)	6,3±0,95<br>4,7-8,7<br>(27)	7,6±1,63<br>5,1-11,9<br>(42)	6,8±2,25<br>5,1-13,7<br>(15)	0,011	0,6709	0,544 [a]<br>0,007 [b]<br>0,266 [c]	N/A
**Urea**<br>mean±SD<br>min-max<br>(N)	**On admission**	mg/dl	60,0±43,9<br>1<br>11-301<br>(191)	39,6±33,38<br>5,0-307,0<br>(936)	74,6±56,42<br>12-353<br>(137)	60,0±45,94<br>8,0-298,0<br>(309)	80,2±51,15<br>19-336<br>(109)	73,2±50,73<br>12,0-289<br>(139)	0,0009	<0.0001	0,032 [a]<br>0,002 [b]<br>0,961 [c]	<0.0001 [a]<br><0.0001 [b]<br>0,025 [c]
mean±SD<br>min-max<br>(N)	**On discharge**		51,9±38,1<br>9<br>9-240<br>(191)	39,0±30,24<br>5,0-307,0<br>(936)	79,4±69,02<br>12-396<br>(137)	60,6±45,74<br>10,0-244,0<br>(309)	83,1±56,85<br>21-342<br>(109)	73,6±47,87<br>15,0-296,0<br>(139)	<0.0001	<0.0001	0,00011 [a]<br><0.0001 [b]<br>0,889 [c]	<0.0001 [a]<br><0.0001 [b]<br>0,019 [c]
**Creatinine**<br>mean±SD<br>min-max<br>(N)	**On admission**		1,5±1,53<br>0,39-<br>14,77<br>(201)	1,1±1,1<br>0,26-14,87<br>(995)	1,7±1,36<br>0,61-9,56<br>(143)	1,4±1,26<br>0,56-12,66<br>(326)	2,0±1,50<br>0,49-9,27<br>(116)	1,6±1,59<br>0,44-11,3<br>(142)	0,014	<0.0001	0,445 [a]<br>0,01 [b]<br>0,16 [c]	0,003 [a]<br>0,0003 [b]<br>0,119 [c]
n (%)<br>(N)		<1.2 mg/dl,	n=125<br>62,2<br>(201)	n=831<br>83,5<br>(995)	n=71<br>49,7<br>(143)	n=217<br>66,6<br>(326)	n=46<br>39,7<br>(116)	n=75<br>52,8<br>(142)	0,003	<0.0001	0,5261 [a]<br>0,0007 [b]<br>0,4284 [c]	<0.0001 [a]<br><0.0001 [b]<br>0,0872 [c]
		1.2-1.9 mg/dl	n=49<br>24,4<br>(201)	n=107<br>10,8<br>(995)	n=44<br>30,8<br>(143)	n=69<br>21,2<br>(326)	n=32<br>27,6<br>(116)	n=37<br>26,1<br>(142)				
		2.0-3.5 mg/dl	n=14<br>7,0<br>(201)	n=30<br>3,0<br>(995)	n=15<br>10,5<br>(143)	n=23<br>7,1<br>(326)	n=23<br>19,8<br>(116)	n=24<br>14,8<br>(142)				
		3.5-4.9 mg/dl	n=8<br>4,0<br>(201)	n=9<br>0,9<br>(995)	n=6<br>4,2<br>(143)	n=8<br>2,5<br>(326)	n=9<br>7,8<br>(116)	n=4<br>2,8<br>(142)				
		≥5 mg/dl	n=5<br>2,5<br>(201)	n=18<br>1,8<br>(995)	n=7<br>4,9<br>(143)	n=9<br>2,8<br>(326)	n=6<br>5,2<br>(116)	n=5<br>3,5<br>(142)				
mean±SD<br>min-max<br>(N)	**On discharge**		1,3±1,51<br>0,4-14,82<br>(201)	1,0±0,94<br>0,26-14,87<br>(995)	1,6±1,40<br>0,57-7,72<br>(143)	1,3±1,32<br>0,36-12,35<br>(326)	1,8±1,58<br>0,43-9,27<br>(116)	1,5±1,31<br>0,43-8,2<br>(142)	0,019	<0.0001	0,138 [a]<br>0,021 [b]<br>0,585 [c]	0,0004 [a]<br><0.0001 [b]<br>0,286 [c]
n (%)<br>(N)		<1.2 mg/dl	n=147<br>73,1<br>(201)	n=858<br>86,2<br>(995)	n=85<br>59,4<br>(143)	n=227<br>69,6<br>(326)	n=51<br>44,0<br>(116)	n=79<br>55,6<br>(142)	<0.0001	<0.0001	0,104 [a]<br><0.0001 [b]<br>0,1 [c]	<0.0001 [a]<br><0.0001 [b]<br>0,0663 [c]
		1.2-1.9 mg/dl	n=30<br>14,9<br>(201)	n=84<br>8,4<br>(995)	n=26<br>18,2<br>(143)	n=59<br>18,1<br>(326)	n=38<br>32,8<br>(116)	n=36<br>25,4<br>(142)				
		2.0-3.4 mg/dl	n=14<br>7,0<br>(201)	n=28<br>2,8<br>(995)	n=15<br>10,5<br>(143)	n=22<br>6,7<br>(326)	n=12<br>10,3<br>(116)	n=19<br>13,4<br>(142)				
		3.5-4.9 mg/dl	n=5<br>2,5<br>(201)	n=11<br>1,1<br>(995)	n=12<br>8,4<br>(143)	n=10<br>3,1<br>(326)	n=9<br>7,8<br>(116)	n=3<br>2,1<br>(142)				
		≥5 mg/dl	n=5<br>2,5<br>(201)	n=14<br>1,4<br>(995)	n=5<br>3,5<br>(143)	n=8<br>2,5<br>(326)	n=6<br>5,2<br>(116)	n=5<br>3,5<br>(142)				
**eGFR**<br>mean±SD<br>min-max<br>(N)	**On admission**	ml/min/1,73 m²	70,2±34,7<br>3,0-239,0<br>(201)	87,7±33,63<br>0-433,0<br>(990)	55,1±27,37<br>4,0-131,0<br>(143)	65,2±28,64<br>4,0-149,0<br>(326)	47,0±38,34<br>6,0-180,0<br>(116)	57,4±30,44<br>5,0-145,0<br>(142)	<0.0001	<0.0001	<0.0001 [a]<br><0.0001 [b]<br>0,667 [c]	<0.0001 [a]<br><0.0001 [b]<br>0,029 [c]
n (%)<br>(N)		<60 ml/min/1,73 m2, n (%)	n=82<br>40,8<br>(201)	n=153<br>15,5<br>(990)	n=77<br>53,8<br>(143)	n=131<br>40,2<br>(326)	n=80<br>69,0<br>(116)	n=80<br>56,3<br>(142)	<0.0001	<0.0001	0,067 [a]<br><0.0001 [b]<br>0,057 [c]	<0.0001 [a]<br><0.0001 [b]<br>0,0053 [c]
		≥60 ml/min/1,73 m2, n (%)	n=119<br>59,2<br>(201)	n=837<br>84,5<br>(990)	n=66<br>46,2<br>(143)	n=195<br>59,8<br>(326)	n=36<br>31,0<br>(116)	n=62<br>43,7<br>(142)				

mean±SD<br>min-max<br>(N)	**On discharge**		78,2±35,15<br>3,0-232,0<br>(201)	91,1±34,20<br>0-433,0<br>(990)	59,4±30,06<br>7,0-151,0<br>(143)	68,2±30,78<br>4,0-208,0<br>(326)	55,9±35,58<br>5,0-209,0<br>(116)	60,8±31,36<br>5,0-148,0<br>(142)	<0.0001	<0.0001	<0.0001 [a]<br><0.0001 [b]<br>0,667 [c]	<0.0001 [a]<br><0.0001 [b]<br>0,05 [c]
n (%)<br>(N)		<60 ml/min/1,73 m2, n (%)	n=56<br>27,9<br>(201)	n=133<br>13,4<br>(990)	n=66<br>46,2<br>(143)	n=116<br>35,6<br>(326)	n=69<br>59,5<br>(116)	n=70<br>49,3<br>(142)	<0.0001	<0.0001	0,002 [a]<br><0.0001 [b]<br>0,133 [c]	<0.0001 [a]<br><0.0001 [b]<br>0,0218 [c]
		≥60 ml/min/1,73 m2, n (%)	n=145<br>72,1<br>(201)	n=857<br>86,6<br>(990)	n=77<br>53,8<br>(143)	n=210<br>64,4<br>(326)	n=47<br>40,5<br>(116)	n=72<br>50,7<br>(142)				
**Total protein**<br>mean±SD<br>min-max<br>(N)	**On admission**	g/l	6,0±0,92<br>4,0-8,1<br>(60)	6,1±0,82<br>3,5-8,2<br>(260)	6,1±0,87<br>4,6-8,7<br>(45)	5,9±0,95<br>3,6-9,5<br>(104)	5,7±0,79<br>3,6-7,8<br>(58)	5,7±0,96<br>3,3-8,2<br>(61)	0,056	0,0198	N/A	N/A
mean±SD<br>min-max<br>(N)	**On discharge**		6,0±0,92<br>4,0-8,1<br>(60)	6,0±0,87<br>3,0-8,2 (260)	5,9±0,86<br>4,5-8,7<br>(45)	5,8±0,95<br>3,7-9,1<br>(104)	5,6±0,88<br>3,4-7,8<br>(58)	5,5±0,93<br>3,3-8,1<br>(61)	0,108	0,0004	N/A	N/A
**Albumin**<br>mean±SD<br>min-max<br>(N)	**On admission**	g/l	3,1±0,58<br>1,6-4,4<br>(73)	3,2±0,61<br>1,5-5,1<br>(291)	3,1±0,53<br>2,1-4,4<br>(52)	3,1±0,57<br>1,1-4,3<br>(105)	3,0±0,58<br>0,7-4,0<br>(68)	2,9±0,62<br>1,5-4,9<br>(58)	0,236	0,021	N/A	N/A
mean±SD<br>min-max<br>(N)	**On discharge**		2,9±0,66<br>1,6-4,3<br>(73)	3,1±0,67<br>0,4-5,1<br>(291)	3,0±0,59<br>1,7-4,4<br>(52)	3,0±0,54<br>1,9-4,3<br>(105)	2,8±0,57<br>1,4-4,5<br>(68)	2,7±0,63<br>0,9-4,2<br>(58)	0,494	0,0009	N/A	0,668 [a]<br>0,0007 [b]<br>0,009 [c]
**Uric acid**<br>mean±SD<br>min-max<br>(N)	**On admission**	mg/dl	6,2±2,93<br>1,9-17,3<br>(66)	5,3±2,4<br>1,4-23,6<br>(274)	6,6±2,69<br>2,7-14,1<br>(49)	6,4±3,11<br>1,7-18,4<br>(109)	6,8±2,57<br>1,7-13,6<br>(59)	6,2±2,25<br>1,9-12,6<br>(51)	0,443	0,0005	N/A	0,002 [a]<br>0,022 [b]<br>0,886 [c]
mean±SD<br>min-max<br>(N)	**On discharge**		5,4±2,62<br>1,1-16,5<br>(66)	5,2±2,22<br>1,6-20,4<br>(274)	5,8±2,51<br>2,0-12,5<br>(49)	6,4±3,22<br>1,9-18,6<br>(109)	6,6±2,56<br>1,7-13,2<br>(59)	5,8±1,88<br>1,9-10,4<br>(51)	0,039	0,0013	0,689 [a]<br>0,031 [b]<br>0,242 [c]	0,002 [a]<br>0,152 [b]<br>0,264 [c]
**AST**<br>mean±SD<br>min-max<br>(N)	**On admission**	IU/L	54,6±71,07<br>5-630<br>(162)	62,0±123,0<br>6,0-2405,0<br>(703)	50,5±46,8<br>10-260<br>(107)	73,6±312,72<br>7,0-4776,0<br>(234)	11,9±409,3<br>10-3866<br>(260)	66,4±237,45<br>8,0-2518,0<br>(110)	0,311	0,8456	N/A	N/A
mean±SD<br>min-max<br>(N)	**On discharge**		43,6±57,14<br>5-446<br>(162)	105,2±943,6<br>8,0-23896,0<br>(703)	112,2±634,5<br>8-6591<br>(107)	52,8±93,48<br>11,0-1224,0<br>(234)	150,4±574,0<br>7-4019<br>(260)	101,6±579,98<br>8,0-6088,0<br>(110)	0,109	0,24498	N/A	N/A
**ALT**<br>mean±SD<br>min-max<br>(N)	**On admission**	IU/L	48,1±66,15<br>4-604<br>(171)	56,7±98,47<br>4,0-1411,0<br>(782)	36,6±28,76<br>6-187<br>(122)	55,2±231,13<br>4,0-3700,0<br>(262)	60,5±168,2<br>5-1361<br>(103)	44,8±121,58<br>6,0-1315,0<br>(117)	0,049	0,5983	0,091 [a]<br>0,755 [b]<br>0,318 [c]	N/A
mean±SD<br>min-max<br>(N)	**On discharge**		49,5±70,10<br>4-604<br>(171)	75,8±213,24<br>4,0-5163,0<br>(782)	55,9±120,40<br>5-1247<br>(122)	47,4±73,84<br>5,0-938,0<br>(262)	85,3±255,10<br>5-1570<br>(103)	63,1±274,32<br>8,0-2985,0<br>(117)	0,353	0,0063	N/A	0,004 [a]<br>0,882 [b]<br>0,814 [c]
**Bilirubin**<br>mean±SD<br>min-max<br>(N)	**On admission**		0,9±1,43<br>0,1-15,1<br>(151)	0,8±1,46<br>0,1-19,1<br>(684)	0,8±0,47<br>0,2-2,8<br>(106)	0,9±0,82<br>0,2-9,2<br>(239)	0,8±0,81<br>0,1-6,6<br>(88)	0,9±0,63<br>0,3-4,2<br>(109)	0,510	0,6969	N/A	N/A
mean±SD<br>min-max<br>(N)	**On discharge**		1,0±2,40<br>0,2-25,9<br>(151)	0,8±1,66<br>0,1-19,0<br>(684)	0,8±0,49<br>0,2-2,8<br>(106)	0,9±2,31<br>0,2-35,3<br>(239)	1,0±1,39<br>0,2-12,8<br>(88)	0,9±0,76<br>0,3-6,1<br>(109)	0,234	0,8534	N/A	N/A
**GGTP**<br>mean±SD<br>min-max<br>(N)	**On admission**	U/L	98,7±159,88<br>10,0-1255,0<br>(144)	90,2±163,22<br>6,0-2532,0<br>(669)	84,1±110,5<br>10,0-696,0<br>(96)	71,3±104,85<br>8,0-975,0<br>(231)	70,2±102,9<br>9,0-625,0<br>(83)	76,3±114,4<br>9,0-687,0<br>(104)	0,265	0,1189	N/A	N/A
mean±SD<br>min-max<br>(N)	**On discharge**		92,1±129,04<br>11,0-91,0<br>(144)	96,0±138,44<br>6,0-1771,0<br>(669)	100,2±140,1<br>10,0-733,0<br>(96)	75,2±112,0<br>7,0-1091,0<br>(231)	62,8±59,61<br>6,0-296,0<br>(83)	87,3±146,95<br>7,0-1207,0<br>(104)	0,012	0,0765	0,892 [a]<br>0,054 [b]<br>0,048 [c]	N/A
**LDH**<br>mean±SD<br>min-max<br>(N)	**On admission**	U/L	433,5±237,88<br>124,0-1521,0<br>(136)	430,4±404,7<br>50,0-7100,0<br>(625)	381,7±197,7<br>134,0-1172,0<br>(88)	391,9±201,09<br>44,0-1357,0<br>(195)	391,6±248,4<br>71,0-1863,0<br>(78)	489,8±986,38<br>106,0-9505,0<br>(89)	0,187	0,1572	N/A	N/A
mean±SD<br>min-max<br>(N)	**On discharge**		369,9±243,39<br>93,0-2133,0<br>(136)	395,2±620,66<br>50,0-11227,0<br>(625)	392,4±217,3<br>118,0-1189,0<br>(88)	357,2±201,12<br>44,0-1584,0<br>(195)	438,0±584,7<br>113,0-5037,0<br>(78)	470,2±983,39<br>97,0-9505,0<br>(89)	0,539	0,2591	N/A	N/A
**Cardiac biomarkers**												
**BNP**<br>mean±SD<br>min-max<br>(N)	**On admission**	pg/ml	188,1±221,49<br>20,0-880,7<br>(37)	232,7±723,72<br>1,7-6924,2<br>(122)	758,3±1070,0<br>3,0-4890,6<br>(35)	275,7±568,93<br>10,3-3153,2<br>(64)	945,2±1242,0<br>12,4-4993,0<br>(47)	1090,3±2576,53<br>5,9-13368,4<br>(51)	<0.0001	0,0699	0,01 [a]<br>0,0005 [b]<br>0,747 [c]	N/A
mean±SD<br>min-max<br>(N)	**On discharge**		170,0±210,86<br>20,0-787,1<br>(37)	236,5±724,47<br>1,7-6924,2<br>(122)	870,3±1868,0<br>3,0-10622,8<br>(35)	250,7±512,08<br>10,3-3153,2<br>(64)	810,6±999,0<br>11,9-3796,0<br>(47)	1047,4±2478,98<br>16,6-13368,4<br>(51)	<0.0001	0,0769	0,084 [a]<br>0,0002 [b]<br>0,984 [c]	N/A

**NT-proBNP**<br>mean±SD<br>min-max<br>(N)	**On admission**	ng/ml	4424±131<br>71<br>29,7-<br>70000<br>(29)	1406,3±<br>6196,8<br>12,0-70000,0<br>(138)	10758,9±163<br>02<br>315,3-70000<br>(30)	76939,7±140<br>78,6<br>18,2-70000,0<br>(77)	15869±1985<br>0<br>119,6-70000<br>(50)	12975,7±19<br>271,1<br>279,9-<br>70000,0<br>(42)	0,012	<0.0001	0,236 [a]<br>0,008 [b]<br>0,429 [c]	0,001 [a]<br>0,001 [b]<br>0,262 [c]
mean±SD<br>min-max<br>(N)	**On discharge**		2802±658<br>2,1<br>29,7-<br>31739,0<br>(29)	1649,0±6991<br>,06<br>12,0-70000,0<br>(138)	12625,5±182<br>84<br>315,3-70000<br>(30)	8025,9±1468<br>3,7<br>18,2-70000,0<br>(77)	14859±1830<br>4<br>119,6-70000<br>(50)	12104,5±17<br>602,5<br>360,8-<br>70000,0<br>(42)	<0.0001	<0.0001	0,027 [a]<br>0,0002 [b]<br>0,858 [c]	0,002 [a]<br>0,001 [b]<br>0,412 [c]
**Troponin I,**<br>mean±SD<br>min-max<br>(N)	**On admission**		256,6±10<br>35,3<br>1,3-<br>7376,0<br>(126)	110,4±751,6<br>0-11758,0<br>(542)	2830,9±1528<br>7<br>2,4-109360,0<br>(89)	1245,6±9904<br>,81<br>1,0-125593,0<br>(209)	1152,0±3112<br>,0<br>3,3-18309,0<br>(87)	427,1±2200<br>,64<br>4,0-21022,9<br>(95)	0,013	0,1022	0,257 [a]<br>0,03 [b]<br>0,569 [c]	N/A
mean±SD<br>min-max<br>(N)	**On discharge**		192,0±94<br>0,39<br>1,9-<br>7396,0<br>(126)	101,2±806,3<br>5<br>0,2-12392,0<br>(542)	3105,5±1537<br>6<br>2,6-109360<br>(89)	1360,1±1253<br>3,8<br>0,8-17463,0<br>(209)	1107,0±4014<br>,0<br>3,9-29828,0	261,1±722,<br>41<br>1,8-5477,3<br>(95)	0,027	0,05673	0,18 [a]<br>0,098 [b]<br>0,464 [c]	N/A
n (%)<br>(N)		≤3-fold upper range<br>K 46.8<br>M 102.6	n=95<br>75,4<br>(126)	n=464<br>85,6<br>(542)	n=54<br>60,7<br>(89)	n=149<br>71,3<br>(209)	n=51<br>58,6<br>(87)	n=49<br>51,6<br>(95)	0,016	<0.0001	0,0934 [a]<br>0,0438 [b]<br>1 [c]	<0.0001 [a]<br><0.0001 [b]<br>0,0039 [c]
		> 3-fold upper range<br>K 46.8<br>M 102.6	n=31<br>24,6<br>(126)	n=78<br>14,4<br>(542)	n=35<br>39,3<br>(89)	n=60<br>28,7<br>(209)	n=36<br>41,4<br>(87)	n=46<br>48,4<br>(95)				
**LDL-cholesterol,**<br>mean±SD<br>min-max<br>(N)		mg/dl	96,3±50,1<br>3<br>27,0-<br>248,0<br>(58)	99,8±50,74<br>6,0-510,0<br>(170)	86,1±42,39<br>37,0-187,0<br>(42)	87,9±39,67<br>17,0-230,0<br>(85)	70,5±41,49<br>14,0-187,0<br>(39)	76,5±43,76<br>6,0-210,0<br>(46)	0,025	0,0063	0,52 [a]<br>0,019 [b]<br>0,219 [c]	0,103 [a]<br>0,008 [b]<br>0,312 [c]
n (%)<br>(N)		>115 mg/dL	n=18<br>31,0<br>(58)	n=53<br>31,2<br>(170)	n=12<br>28,6<br>(42)	n=17<br>20,0<br>(85)	n=7<br>17,9<br>(39)	n=7<br>15,2<br>(46)	0,339	0,0337	N/A	0,2475 [a]<br>0,151 [b]<br>1 [c]
		≤115 mg/dL	n=40<br>69,0<br>(58)	n=117<br>68,8<br>(170)	n=30<br>71,4<br>(42)	n=68<br>80,0<br>(85)	n=32<br>82,1<br>(39)	n=39<br>84,8<br>(46)				
**HDL-cholesterol**<br>mean±SD<br>min-max<br>(N)		mg/dl	38,8±14,7<br>1<br>15,0-82,0<br>(58)	39,9±16,43<br>2,0-120,0<br>(174)	39,2±13,23<br>17,0-73,0<br>(42)	40,7±16,29<br>7,0-110,0<br>(85)	34,5±13,81<br>17,0-68,0<br>(38)	38,2±14,85<br>8,0-79,0<br>(45)	0,237	0,6911	N/A	N/A
**Triglycerides**<br>mean±SD<br>min-max<br>(N)		mg/dl	187,8±10<br>7,13<br>44,0-<br>760,0<br>(90)	173,6±127,2<br>8<br>40,0-1100,0<br>(261)	146,4±77,58<br>54,0-507,0	144,3±105,4<br>4<br>48,0-595,0<br>(110)	130,6±48,92<br>53,0-282,0	125,9±71,2<br>1<br>16,0-413,0<br>(58)	0,0002	0,00051	0,025 [a]<br><0.0001<br>[b]<br>0,434 [c]	0,058 [a]<br>0,00042 [b]<br>0,379 [c]
n (%)<br>(N)		>150 mg/dL	n=52<br>57,8<br>(90)	n=117<br>44,8<br>(261)	n=20<br>39,2<br>(51)	n=30<br>27,3<br>(110)	n=20<br>36,4<br>(55)	n=15<br>25,9<br>(58)	0,019	0,0008	0,1559 [a]<br>0,0592 [b]<br>1 [c]	0,007 [a]<br>0,0367 [b]<br>1 [c]
		≤150 mg/dL	n=38<br>42,2<br>(90)	n=144<br>55,2<br>(261)	n=31<br>60,8<br>(51)	n=80<br>72,7<br>(110)	n=35<br>63,6<br>(55)	n=43<br>74,1<br>(58)				
**Hormones**												
**25-hydroxyvitamin D**<br>mean±SD<br>min-max<br>(N)		ng/ml	24,1±14,5<br>7<br>4,0-73,2<br>(56)	25,0±18,33<br>3,5-146,1<br>(243)	23,8±15,84<br>4,9-75,6<br>(35)	25,1±17,12<br>3,5-77,7<br>(71)	16,4±11,86<br>3,5-44,6<br>(29)	21,7±16,69<br>3,5-63,5<br>(32)	0,025	0,5616	0,995 [a]<br>0,029 [b]<br>0,092 [c]	N/A
**TSH**<br>mean±SD<br>min-max<br>(N)		mIU/l	1,4±2,56<br>0,08-18,6<br>(86)	1,3±1,18<br>0-8,09<br>(346)	2,1±3,98<br>0,01-28,81<br>(68)	1,3±1,32<br>0,01-12,1<br>(159)	1,7±1,56<br>0-7,86<br>(70)	2,8±5,39<br>0-38,24<br>(72)	0,403	0,0739	N/A	N/A
**fT4**<br>mean±SD<br>min-max<br>(N)		pmol/L,	12,4±2,20<br>6,79-<br>15,96<br>(35)	12,8±3,1<br>6,68-33,47<br>(144)	12,9±2,49<br>5,92-18,54<br>(31)	12,7±3,87<br>6,18-36,6<br>(58)	13,32±3,31<br>8,15-22,42<br>(32)	13,5±4,92<br>7,87-35,46<br>(28)	0,380	0,739	N/A	N/A
**fT3**<br>mean±SD<br>min-max<br>(N)		pmol/l	2,0±0,65<br>0,96-3,64<br>(34)	2,4±2,11<br>0,95-25,25<br>(138)	1,7±0,61<br>0,95-3,01<br>(28)	1,9±0,82<br>0,95-4,45<br>(53)	1,8±0,67<br>0,95-4,13<br>(28)	2,3±1,33<br>0,98-6,85<br>(23)	0,206	0,0338	N/A	0,03 [a]<br>0,9 [b]<br>0,393 [c]
**Cortisol**<br>mean±SD<br>min-max<br>(N)		µg/dl	13,4±14,8<br>9<br>0,8-57,5<br>(13)	13,8±16,64<br>0,1-119,6<br>(65)	18,8±19,01<br>1,0-59,8<br>(7)	12,7±10,56<br>1,9-42,3<br>(15)	14,9±9,30<br>0,9-29,6<br>(9)	9,6±4,96<br>2,3-17,4<br>(8)	0,816	0,3064	N/A	N/A

Suppl. Table S3. Therapies applied during the hospitalisation in the diabetic and non-diabetic cohort.

**Variables, units**	**Low risk [0-1]**		**Medium [2-3]**		**High risk [≥4]**		**ANOVA P value**		**P value for post-hoc analysis in significant ANOVA L-M a L-H b M-H c**	
**Applied treatment and procedures**	Diabetes N=209	Non-Diabetes N=1183	Diabetes N= 146	Non-Diabetes N=337	Diabetes N=118	Non-Diabetes N=146	Diabetes	Non-Diabetes	Diabetes	Non-Diabetes
**Systemic corticosteroid,** n n (%)	117 (56,0)	579 (48,9)	77 (52,7)	165 (49,0)	57 (48,3)	81 (55,5)	0,407973	0,320829	N/A	N/A
**Convalescent plasma,** n n (%)	31 (14,8)	134 (11,3)	15 (10,3)	26 (7,7)	14 (11,9)	17 (11,6)	0,42653	0,151155	N/A	N/A
**Tocilizumab,** n n (%)	2 (1,0)	20 (1,7)	1 (0,7)	1 (0,3)	1 (0,8)	0 (0,0)	1,0	0,054935	N/A	N/A
**Remdesivir,** n n (%)	38 (18,2)	196 (16,6)	14 (9,6)	56 (16,6)	14 (11,9)	21 (14,4)	0,053466	0,790951	N/A	N/A
**Antibiotic,** n n (%)	139 (66,5)	592 (50,0)	100 (68,5)	200 (59,3)	82 (69,5)	103 (70,5)	0,841033	<0.0001	N/A	0,0094 [a] <0.0001 [b] 0,0761 [c]

Suppl. Table S4. The Log-rank statistics for matching the C2HEST risk strata for total mortality in diabetic and non-diabetic cohort.

DIABETICS	h2	h3	h4	h5	**h6**	h7	h8
m1	38.8304	35.091	20.0129	16.6071	15.5571	4.7492	2.1734
**m2**		41.2091	40.319	41.4568	**42.7869**	38.9894	6.2263
m3			35.3244	36.5235	37.9713	35.3315	5.9124
m4				19.3965	20.8289	18.7107	4.2634
m5					15.2157	14.8423	3.7087
m6						14.603	3.4843
m7							0.2581

NON-DIABETICS	h2	h3	**h4**	h5	h6	h7	h8
m1	214.2007	182.8439	193.6593	164.2116	132.8852	134.8876	11.4564
**m2**		208.9715	**227.9922**	217.4597	205.7872	207.3228	14.344
m3			157.3889	146.7787	138.1358	139.0401	11.7401

m4				122.9856	127.4897	123.0078	11.0815
m5					73.2758	66.1284	8.1231
m6						10.0848	2.8032
m7							2.5976

m-medium, h-high

Suppl. Table S5. The Log-rank statistics for matching the C2HEST risk strata for in-hospital mortality in diabetic and non-diabetic cohort.

DIABETICS	h2	h3	h4	h5	**h6**	h7	h8
m1	14.4123	13.6351	4.4525	3.4907	8.9395	1.2788	0.8757
**m2**		15.3486	14.0526	14.2558	**18.2982**	14.1303	3.7454
m3			13.7242	13.5861	17.0953	13.6078	3.6824
m4				4.4599	9.1179	4.3109	2.0553
m5					8.7966	3.106	1.7534
m6						9.0862	2.9295
m7							0.7543

NON-DIABETIC	h2	h3	**h4**	h5	h6	h7	h8
m1	51.6561	51.3363	58.8525	47.6171	27.0761	34.1247	4.9027
**m2**		57.4548	**68.9848**	64.3097	52.347	59.3837	7.1777
m3			59.5348	56.4524	48.0836	54.6571	6.9204
m4				52.1425	51.4903	55.4325	7.1361
m5					35.9538	37.6049	5.8164
m6						12.8644	2.385
m7							3.4673

m-medium, h-high

Suppl. Table S6. Odds ratios for quantifying the strength of the association between CH2EST-score and study endpoints and adverse events in diabetic and non-diabetic cohort

Endpoint – DIABETIC cohort	Comparison	OR	CI min.	CI max.	P value
End of hospitalization - death	overall	0.817	0.733	0.908	0.0002
	low vs medium	0.461	0.299	0.709	0.0004
	low vs high	0.425	0.266	0.674	0.0003

End of hospitalization – deterioration	overall	1.044	0.906	1.198	0.546
	low vs medium	1.272	0.700	2.297	0.4259
	low vs high	1.319	0.699	2.453	0.3844
End of hospitalization - rehabilitation	overall	0.979	0.842	1.131	0.778
	low vs medium	1.221	0.674	2.195	0.5054
	low vs high	0.836	0.412	1.627	0.6056
End of hospitalization – full recovery	overall	1.269	1.130	1.428	< 0.0001
	low vs medium	2.234	1.332	3.778	0.0024
	low vs high	2.945	1.732	5.054	< 0.0001
Shock – all cause	overall	0.976	0.832	1.137	0.7636
	low vs medium	0.658	0.326	1.274	0.2256
	low vs high	0.974	0.495	1.857	0.9365
Cardiogenic shock	overall	1.532	1.163	2.044	0.0026
	low vs medium	2.915	0.561	21.234	0.2202
	low vs high	7.527	1.848	50.448	0.0116
Septic shock	overall	0.919	0.767	1.089	0.3441
	low vs medium	0.496	0.222	1.028	0.0698
	low vs high	0.763	0.359	1.537	0.4623
Pulmonary embolism	overall	0.902	0.698	1.136	0.4016
	low vs medium	0.990	0.399	2.361	0.9829
	low vs high	0.393	0.089	1.251	0.1519
Deep vein thrombosis	overall	0.764	0.286	1.485	0.4975
	low vs medium	0.714	0.033	7.519	0.7839
	low vs high	NA	NA	NA	NA
Myocardial infarction	overall	1.152	0.838	1.552	0.3616
	low vs medium	1.444	0.336	6.196	0.6078
	low vs high	1.798	0.418	7.735	0.4129
Myocardial injury	overall	1.173	1.033	1.334	0.0142
	low vs medium	1.986	1.106	3.591	0.0221
	low vs high	2.163	1.204	3.917	0.0102
Acute heart failure	overall	1.943	1.586	2.425	< 0.0001
	low vs medium	6.799	1.720	45.041	0.0152
	low vs high	25.058	7.215	158.171	< 0.0001
Stroke/TIA	overall	1.066	0.784	1.413	0.6662
	low vs medium	2.971	0.917	11.308	0.0801
	low vs high	0.884	0.121	4.598	0.8874
Complete respiratory failure	overall	1.294	1.022	1.668	0.0377
	low vs medium	1.961	0.754	5.245	0.1712
	low vs high	2.745	1.016	7.826	0.0509
SIRS	overall	1.053	0.900	1.225	0.5087
	low vs medium	1.049	0.532	2.033	0.8891
	low vs high	1.339	0.675	2.613	0.3945
SEPSiS	overall	1.154	0.802	1.639	0.4213
	low vs medium	2.941	0.553	21.786	0.2228
	low vs high	1.415	0.166	12.097	0.7325
Acute kidney injury	overall	1.205	1.061	1.369	0.0041
	low vs medium	1.467	0.821	2.617	0.1933
	low vs high	2.303	1.301	4.097	0.0042
Acute liver dysfunction.	overall	1.193	0.924	1.524	0.1626
	low vs medium	1.221	0.346	4.136	0.7455
	low vs high	2.196	0.712	6.986	0.1673
MODS	overall	1.310	0.940	1.813	0.101
	low vs medium	2.171	0.356	16.641	0.399
	low vs high	4.580	0.970	32.334	0.0717
All bleedings	overall	1.050	0.863	1.264	0.6167
	low vs medium	0.850	0.348	1.966	0.7087
	low vs high	1.330	0.576	2.982	0.4922
Intracranial bleeding	overall	0.822	0.449	1.312	0.4594
	low vs medium	1.441	0.263	7.879	0.6577

	low vs high	NA	NA	NA	NA
Respiratory tract bleeding	overall	1.005	0.721	1.353	0.9759
	low vs medium	0.710	0.148	2.737	0.6319
	low vs high	1.187	0.298	4.242	0.7937
Upper-GI-tract bleeding	overall	0.938	0.640	1.306	0.7218
	low vs medium	0.470	0.068	2.073	0.3592
	low vs high	0.883	0.184	3.412	0.8617
Lower-GI- tract bleeding	overall	1.202	0.623	2.171	0.5408
	low vs medium	NA	NA	NA	NA
	low vs high	3.586	0.340	77.658	0.2992
Urinary tract bleeding	overall	1.111	0.721	1.638	0.6056
	low vs medium	0.954	0.124	5.825	0.9589
	low vs high	1.184	0.154	7.243	0.8544
Pneumonia	overall	1.076	0.968	1.199	0.1778
	low vs medium	1.038	0.673	1.605	0.8673
	low vs high	1.213	0.760	1.949	0.4212

Endpoint – NON-DIABETIC cohort	Comparison	OR	CI min.	CI max.	P value
End of hospitalization - death	overall	0.706	0.658	0.756	< 0.0001
	low vs medium	0.370	0.288	0.474	< 0.0001
	low vs high	0.243	0.169	0.346	< 0.0001
End of hospitalization – deterioration	overall	1.197	1.096	1.303	< 0.0001
	low vs medium	2.532	1.819	3.507	< 0.0001
	low vs high	1.806	1.088	2.890	0.0172
End of hospitalization - rehabilitation	overall	0.961	0.863	1.063	0.4537
	low vs medium	0.978	0.660	1.420	0.9108
	low vs high	0.752	0.396	1.319	0.3503
End of hospitalization – full recovery	overall	1.582	1.455	1.721	< 0.0001
	low vs medium	3.087	2.172	4.372	< 0.0001
	low vs high	7.582	5.062	11.326	< 0.0001
Shock – all cause	overall	1.172	1.049	1.302	0.004
	low vs medium	1.371	0.866	2.119	0.1651
	low vs high	1.793	0.983	3.090	0.0443
Cardiogenic shock	overall	1.636	1.307	2.041	< 0.0001
	low vs medium	4.998	1.585	16.981	0.0063
	low vs high	10.097	3.004	35.441	0.0002
Septic shock	overall	1.103	0.961	1.253	0.1462
	low vs medium	1.094	0.619	1.847	0.7446
	low vs high	1.274	0.578	2.504	0.5119
Pulmonary embolism	overall	1.117	0.981	1.260	0.0838
	low vs medium	1.062	0.611	1.766	0.8227
	low vs high	1.592	0.800	2.925	0.1562
Deep vein thrombosis	overall	1.098	0.797	1.432	0.5243
	low vs medium	0.876	0.199	2.778	0.8389
	low vs high	1.355	0.210	5.035	0.6925
Myocardial infarction	overall	1.471	1.113	1.906	0.0042
	low vs medium	5.343	1.517	21.002	0.0098
	low vs high	6.184	1.208	28.319	0.0178
Myocardial injury	overall	1.444	1.312	1.593	< 0.0001
	low vs medium	2.395	1.629	3.514	< 0.0001
	low vs high	5.585	3.496	8.940	< 0.0001
Acute heart failure	overall	2.066	1.767	2.439	< 0.0001
	low vs medium	7.871	3.085	22.552	< 0.0001
	low vs high	36.682	15.579	100.860	< 0.0001
Stroke/TIA	overall	1.216	0.968	1.490	0.073
	low vs medium	2.752	1.165	6.313	0.0173
	low vs high	1.888	0.429	5.942	0.3256

Complete respiratory failure	overall	1.167	0.995	1.377	0.0614
	low vs medium	0.978	0.494	1.930	0.9483
	low vs high	1.909	0.859	4.378	0.1176
SIRS	overall	0.998	0.893	1.109	0.9781
	low vs medium	0.701	0.438	1.082	0.1222
	low vs high	1.306	0.757	2.149	0.313
SEPSiS	overall	1.432	1.097	1.836	0.0054
	low vs medium	1.136	0.168	4.770	0.8749
	low vs high	5.775	1.664	18.671	0.0036
Acute kidney injury	overall	1.299	1.180	1.427	< 0.0001
	low vs medium	1.781	1.180	2.647	0.005
	low vs high	2.811	1.701	4.513	< 0.0001
Acute liver dysfunction.	overall	1.300	1.109	1.510	0.0008
	low vs medium	2.361	1.233	4.427	0.008
	low vs high	2.320	0.908	5.227	0.0557
MODS	overall	1.106	0.864	1.369	0.3872
	low vs medium	0.923	0.304	2.314	0.8737
	low vs high	1.285	0.299	3.830	0.6892
All bleedings	overall	1.211	1.061	1.371	0.0034
	low vs medium	1.179	0.641	2.058	0.5781
	low vs high	2.508	1.301	4.556	0.0038
Intracranial bleeding	overall	1.168	0.849	1.528	0.291
	low vs medium	1.965	0.600	5.726	0.2289
	low vs high	0.900	0.049	4.837	0.9203
Respiratory tract bleeding	overall	0.989	0.715	1.287	0.938
	low vs medium	0.204	0.011	0.999	0.1232
	low vs high	1.439	0.333	4.348	0.5651
Upper-GI-tract bleeding	overall	1.492	1.202	1.834	0.0002
	low vs medium	2.662	0.871	7.708	0.0716
	low vs high	7.397	2.558	20.912	0.0001
Lower-GI- tract bleeding	overall	1.020	0.536	1.598	0.9403
	low vs medium	0.701	0.037	4.367	0.7463
	low vs high	NA	NA	NA	NA
Urinary tract bleeding	overall	1.305	0.932	1.746	0.0901
	low vs medium	1.171	0.171	5.110	0.847
	low vs high	4.115	0.861	15.777	0.0471
Pneumonia	overall	1.350	1.259	1.451	< 0.0001
	low vs medium	1.936	1.512	2.485	< 0.0001
	low vs high	2.714	1.884	3.968	< 0.0001

Supplementary Table S7. Discriminatory performance of the C_2HEST score on the clinical events in diabetic and non-diabetic cohorts

CLINICAL EVENT – DIABETIC COHORT	AUC	SENSITIVITY	SPECIFICITY
**End of hospitalization - full recovery**	0.6024071	0.5479452	0.6496063
**End of hospitalization - deterioration**	0.5254052	0.6111111	0.4513716
**End of hospitalization - rehabilitation**	0.4946327	0.9104478	0.1083744
**End of hospitalization - death**	0.6240952	0.7217391	0.4944134
**All-cause shock**	0.4875133	0.2711864	0.7536232
**Hypovolemic shock**	0.6439176	0.8888889	0.4525862
**Cardiogenic shock**	0.7252568	0.5714286	0.7603486
**Septic shock**	0.5416827	0.5510204	0.5707547
**Pulmonary embolism**	0.5465179	0.8800000	0.2566964
**Deep vein thrombosis**	0.6007092	1.0000000	0.2510638
**Venous thromboembolic disease**	0.5465179	0.8800000	0.2566964
**Myocardial infarction**	0.5928236	0.6666667	0.5596529
**Myocardial injury**	0.5962990	0.6960784	0.4750000
**Acute heart failure**	0.8186051	0.8823529	0.5876993
**Stroke/TIA**	0.5565671	0.7142857	0.4466231

CLINICAL EVENT – DIABETIC COHORT	AUC	SENSITIVITY	SPECIFICITY
Complete respiratory failure	0.6213204	0.5862069	0.6341463
SIRS	0.5165456	0.5087719	0.5593220
Sepsis	0.5834497	0.7500000	0.4189944
Acute kidney injury	0.6031639	0.5813953	0.5839793
Acute liver dysfunction	0.5929412	0.2777778	0.8729412
MODS	0.6596112	0.8000000	0.5615551
All bleedings	0.5166667	0.1428571	0.9337900
Intracranial bleeding	0.5635261	1.0000000	0.2526767
Respiratory tract bleeding	0.4903010	0.1538462	0.9304348
Upper-GI-tract bleeding	0.5453562	0.5454545	0.5606061
Lower-GI-tract-bleeding	0.6312057	0.6666667	0.7531915
Urinary tract bleeding	0.5234519	0.2857143	0.9313305
Pneumonia	0.5348402	0.4659864	0.5865922

CLINICAL EVENT – NON-DIABETIC COHORT	AUC	SENSITIVITY	SPECIFICITY
End of hospitalization - full recovery	0.6449670	0.7945845	0.4420168
End of hospitalization - deterioration	0.5979705	0.4558824	0.7332421
End of hospitalization - rehabilitation	0.4948965	0.5026738	0.5043949
End of hospitalization - death	0.7165027	0.5833333	0.7510260
All-cause shock	0.5923052	0.6776860	0.5177994
Hypovolemic shock	0.6020403	0.6923077	0.5067073
Cardiogenic shock	0.7493595	0.7222222	0.7148058
Septic shock	0.5668229	0.6588235	0.5123340
Pulmonary embolism	0.5395282	0.2446809	0.8237913
Deep vein thrombosis	0.4982521	0.1176471	0.9836264
Venous thromboembolic disease	0.5342675	0.2376238	0.8236422
Myocardial infarction	0.7249058	0.6923077	0.7132486
Myocardial injury	0.6678092	0.5760870	0.7009063
Acute heart failure	0.8675009	0.8571429	0.7247537
Stroke/TIA	0.6088415	0.5000000	0.7134146
Complete respiratory failure	0.5578676	0.2183908	0.9425287
SIRS	0.5165473	0.5312500	0.5187500
Sepsis	0.6915287	0.5000000	0.8201220
Acute kidney injury	0.6272756	0.6896552	0.5220250
Acute liver dysfunction	0.6590205	0.8125000	0.4868056
MODS	0.5569792	0.6296296	0.5057962
All bleedings	0.5724504	0.2948718	0.8255668
Intracranial bleeding	0.5760549	0.4000000	0.8219261
Respiratory tract bleeding	0.4778550	0.1428571	0.9130699
Upper-GI-tract bleeding	0.6978289	0.6190476	0.7142857
Lower-GI-tract-bleeding	0.5912149	0.8333333	0.5048193
Urinary tract bleeding	0.5825872	0.2727273	0.9558912
Pneumonia	0.6273602	0.6137690	0.6279357

Supplementary Table S8. The in-hospital all-cause-death Hazard Ratios for modified C2HEST risk stratification - Replace "thyroid disease" by "hypothyroidism" and "age>75 years" by "age>65 years" in diabetes cohort

Total deaths – DIABETIC COHORT	HR	CI min.	CI max.	p-value
Overall	1.207286	1.0841998	1.344345	0.0005959
low vs medium	1.683207	0.6456338	4.388225	0.2868538
low vs high	2.499916	1.0123108	6.173576	0.0469788

Total deaths – NON-DIABETIC COHORT	HR	CI min.	CI max.	p-value
Overall	1.336532	1.246332	1.43326	0
low vs medium	2.458144	NA	NA	NA
low vs high	4.014245	NA	NA	NA

Suppl. Table S9. Odds ratios for quantifying the strength of the association between the modified CH2EST-score and study endpoints and adverse events - replacing "thyroid disease" with "hypothyroidism" and cut-off point for age to ">65 years" as scoring items in diabetic and non-diabetic cohorts.

Endpoint – DIABETIC COHORT	Comparison	OR	CI min.	CI max.	P value
End of hospitalization - death	overall	1.256	1.107	1.431	0.0005
	low vs medium	2.112	0.812	6.595	0.1535
	low vs high	2.938	1.221	8.743	0.0285
End of hospitalization - deterioration	overall	1.074	0.927	1.246	0.3443
	low vs medium	0.798	0.314	2.213	0.646
	low vs high	1.004	0.446	2.572	0.993
End of hospitalization - rehabilitation	overall	0.935	0.802	1.089	0.3913
	low vs medium	0.975	0.393	2.665	0.9582
	low vs high	0.815	0.358	2.102	0.6452
End of hospitalization - full recovery	overall	0.841	0.753	0.937	0.0019
	low vs medium	0.760	0.376	1.517	0.4384
	low vs high	0.581	0.304	1.095	0.0951
All-cause shock	overall	1.006	0.856	1.181	0.9441
	low vs medium	0.630	0.239	1.786	0.3618
	low vs high	0.769	0.337	1.989	0.5566
Hypovolemic shock	overall	0.699	0.455	1.041	0.0864
	low vs medium	0.712	0.134	5.269	0.7008
	low vs high	0.207	0.033	1.607	0.0897
Cardiogenic shock	overall	1.432	1.042	2.000	0.0296
	low vs medium	0.717	0.067	15.650	0.7878
	low vs high	1.598	0.299	29.572	0.6575
Septic shock	overall	0.973	0.817	1.158	0.7582
	low vs medium	0.628	0.223	1.927	0.3894
	low vs high	0.737	0.307	2.057	0.5226
pulmonary embolism	overall	0.920	0.723	1.166	0.494

Endpoint – DIABETIC COHORT	Comparison	OR	CI min.	CI max.	P value
	low vs medium	1.278	0.295	8.809	0.7651
	low vs high	1.155	0.313	7.467	0.8515
deep vein thrombosis	overall	0.829	0.403	1.622	0.5874
	low vs medium	NA	NA	NA	NA
	low vs high	NA	NA	NA	NA
myocardial infarction	overall	1.180	0.842	1.664	0.3378
	low vs medium	0.717	0.067	15.650	0.7878
	low vs high	1.299	0.235	24.243	0.8064
myocardial injury	overall	1.189	1.032	1.376	0.018
	low vs medium	3.798	0.968	25.322	0.0921
	low vs high	4.937	1.367	31.659	0.0358
acute heart failure	overall	1.703	1.364	2.158	< 0.0001
	low vs medium	1.458	0.208	28.939	0.7393
	low vs high	4.486	0.921	80.942	0.1451
stroke/TIA	overall	1.048	0.766	1.435	0.7664
	low vs medium	1.084	0.135	22.235	0.9449
	low vs high	1.448	0.267	26.899	0.7274
complete respiratory failure	overall	1.143	0.915	1.442	0.2447
	low vs medium	0.738	0.151	3.436	0.6979
	low vs high	1.367	0.312	5.653	0.6637
SIRS	overall	1.070	0.909	1.261	0.4156
	low vs medium	0.679	0.261	1.912	0.4406
	low vs high	0.683	0.297	1.775	0.3967
Sepsis	overall	1.361	0.901	2.138	0.1558
	low vs medium	NA	NA	NA	NA
	low vs high	NA	NA	NA	NA
Acute kidney injury	overall	1.186	1.033	1.366	0.0165
	low vs medium	1.845	0.645	6.657	0.2917
	low vs high	2.582	0.994	8.834	0.0807
Acute liver dysfunction	overall	0.985	0.742	1.303	0.913
	low vs medium	2.923	0.512	55.140	0.3195
	low vs high	1.208	0.218	22.593	0.8593
MODS	overall	1.848	1.247	2.872	0.0035
	low vs medium	NA	NA	NA	NA
	low vs high	NA	NA	NA	NA
All bleedings	overall	0.975	0.796	1.194	0.8094
	low vs medium	0.547	0.172	1.902	0.3148
	low vs high	0.602	0.231	1.878	0.333
Intracranial bleeding	overall	0.779	0.468	1.256	0.3139
	low vs medium	0.355	0.014	9.117	0.4679
	low vs high	0.568	0.082	11.243	0.6163
Respiratory tract bleeding	overall	0.940	0.676	1.300	0.7079
	low vs medium	0.083	0.004	0.578	0.0277
	low vs high	0.268	0.080	1.039	0.0379
Upper-GI-tract bleeding	overall	0.729	0.497	1.043	0.0912
	low vs medium	0.897	0.186	6.431	0.8994
	low vs high	0.277	0.052	2.043	0.1456
Lower-GI-tract-bleeding	overall	0.829	0.403	1.622	0.5874
	low vs medium	NA	NA	NA	NA
	low vs high	NA	NA	NA	NA
Urinary tract bleeding	overall	0.948	0.605	1.471	0.8113
	low vs medium	NA	NA	NA	NA
	low vs high	0.348	0.072	2.481	0.2154
Pneumonia	overall	1.092	0.979	1.220	0.1177
	low vs medium	1.360	0.677	2.727	0.3846
	low vs high	1.659	0.873	3.138	0.119

Endpoint – NON-DIABETIC COHORT	Comparison	OR	CI min.	CI max.	P value
End of hospitalization - death	overall	1.641	1.509	1.787	< 0.0001
	low vs medium	3.844	2.470	6.110	< 0.0001
	low vs high	8.892	5.891	13.809	< 0.0001
End of hospitalization - deterioration	overall	1.212	1.116	1.314	< 0.0001
	low vs medium	3.094	2.159	4.472	< 0.0001
	low vs high	2.442	1.663	3.601	< 0.0001
End of hospitalization - rehabilitation	overall	1.055	0.965	1.152	0.2327
	low vs medium	1.233	0.855	1.769	0.257
	low vs high	1.259	0.864	1.823	0.2256
End of hospitalization - full recovery	overall	0.674	0.631	0.718	< 0.0001
	low vs medium	0.333	0.259	0.428	< 0.0001
	low vs high	0.224	0.173	0.290	< 0.0001
All-cause shock	overall	1.233	1.114	1.362	< 0.0001
	low vs medium	1.858	1.162	2.976	0.0096
	low vs high	2.518	1.603	3.984	< 0.0001
Hypovolemic shock	overall	1.183	0.951	1.449	0.1158
	low vs medium	0.915	0.280	2.666	0.8744
	low vs high	2.497	1.048	6.164	0.0398
Cardiogenic shock	overall	1.608	1.274	2.032	< 0.0001
	low vs medium	2.767	0.676	13.541	0.1648
	low vs high	6.261	1.903	28.040	0.0055
Septic shock	overall	1.191	1.055	1.339	0.004
	low vs medium	1.894	1.101	3.276	0.0209
	low vs high	2.210	1.290	3.813	0.0039
pulmonary embolism	overall	1.204	1.074	1.347	0.0013
	low vs medium	1.210	0.699	2.065	0.4881
	low vs high	2.167	1.334	3.534	0.0018
deep vein thrombosis	overall	1.099	0.824	1.420	0.493
	low vs medium	2.219	0.767	6.779	0.1422
	low vs high	0.920	0.193	3.505	0.9063
myocardial infarction	overall	1.581	1.202	2.079	0.0009
	low vs medium	6.644	0.979	130.177	0.0907
	low vs high	14.993	2.738	278.625	0.0108
myocardial injury	overall	1.457	1.324	1.609	< 0.0001
	low vs medium	2.743	1.728	4.441	< 0.0001
	low vs high	4.601	2.955	7.339	< 0.0001
acute heart failure	overall	2.147	1.814	2.574	< 0.0001
	low vs medium	16.826	3.208	309.310	0.0072
	low vs high	61.523	13.148	1097.226	< 0.0001
stroke/TIA	overall	1.236	1.000	1.510	0.042
	low vs medium	1.900	0.678	5.451	0.2179
	low vs high	2.944	1.150	8.053	0.0267
complete respiratory failure	overall	1.221	1.036	1.450	0.0192
	low vs medium	0.703	0.306	1.609	0.4045
	low vs high	1.574	0.734	3.403	0.2443
SIRS	overall	1.026	0.930	1.127	0.6051
	low vs medium	0.964	0.648	1.422	0.8563
	low vs high	1.022	0.682	1.515	0.9156
Sepsis	overall	1.363	1.034	1.774	0.0227
	low vs medium	0.345	0.018	2.163	0.3333
	low vs high	3.220	1.057	10.807	0.0432
Acute kidney injury	overall	1.354	1.235	1.484	< 0.0001
	low vs medium	2.826	1.797	4.507	< 0.0001
	low vs high	4.010	2.590	6.323	< 0.0001
Acute liver dysfunction	overall	1.258	1.076	1.463	0.0033
	low vs medium	2.473	1.127	5.691	0.0265
	low vs high	3.780	1.817	8.425	0.0006

Endpoint – NON-DIABETIC COHORT	Comparison	OR	CI min.	CI max.	P value
MODS	overall	1.067	0.847	1.315	0.5619
	low vs medium	1.325	0.502	3.382	0.5564
	low vs high	1.671	0.659	4.178	0.2679
All bleedings	overall	1.153	1.014	1.304	0.0264
	low vs medium	0.844	0.455	1.512	0.5769
	low vs high	1.602	0.949	2.688	0.0747
Intracranial bleeding	overall	1.152	0.857	1.503	0.3188
	low vs medium	0.548	0.080	2.388	0.4621
	low vs high	2.167	0.715	6.771	0.167
Respiratory tract bleeding	overall	1.038	0.793	1.318	0.7708
	low vs medium	0.747	0.234	2.067	0.5905
	low vs high	0.835	0.262	2.312	0.7394
Upper-GI-tract bleeding	overall	1.407	1.127	1.746	0.0021
	low vs medium	2.914	0.875	11.176	0.0893
	low vs high	4.690	1.558	17.181	0.0094
Lower-GI-tract-bleeding	overall	1.105	0.666	1.679	0.6639
	low vs medium	4.972	0.635	100.678	0.1654
	low vs high	3.695	0.353	79.639	0.2865
Urinary tract bleeding	overall	1.378	1.009	1.851	0.0357
	low vs medium	0.411	0.021	2.788	0.4269
	low vs high	2.787	0.792	10.952	0.1139
Pneumonia	overall	1.333	1.253	1.421	< 0.0001
	low vs medium	2.704	2.137	3.429	< 0.0001
	low vs high	3.133	2.449	4.021	< 0.0001

Suppl. Table S10. Components of C_2HEST score and the risk of outcomes in univariate Cox proportional hazard model (all-cause death) and competing risk regression model (other outcomes) in diabetes cohort.

Endpoint – DIABETIC COHORT	Component	OR	CI min.	CI max.	P value
End of hospitalization - death	Coronary artery disease	2.504	1.450	4.322	0.001
	COPD	0.802	0.270	2.088	0.6684
	Age>75	3.014	1.912	4.780	< 0.0001
	Thyroid disease	0.431	0.191	0.887	0.0304
	Hypertension	0.541	0.312	0.950	0.0299
	HFrEF	1.118	0.638	1.927	0.6919
End of hospitalization - rehabilitation	Coronary artery disease	1.184	0.590	2.282	0.6234
	COPD	1.441	0.455	3.819	0.4927
	Age>75	0.597	0.320	1.069	0.0925
	Thyroid disease	1.712	0.813	3.393	0.1372
	Hypertension	1.802	0.883	4.084	0.1277
	HFrEF	0.808	0.391	1.591	0.549
End of hospitalization - full recovery	Coronary artery disease	0.608	0.366	0.998	0.051
	COPD	0.639	0.261	1.477	0.306
	Age>75	0.480	0.319	0.718	0.0004
	Thyroid disease	1.201	0.685	2.106	0.5216
	Hypertension	1.056	0.656	1.703	0.8234
	HFrEF	0.934	0.573	1.521	0.7843
All-cause shock	Coronary artery disease	0.895	0.419	1.807	0.7654
	COPD	0.496	0.077	1.785	0.3575
	Age>75	0.628	0.326	1.157	0.1476
	Thyroid disease	0.880	0.346	1.949	0.7685
	Hypertension	0.788	0.408	1.609	0.4929
	HFrEF	1.832	0.913	3.591	0.0815
Hypovolemic shock	Coronary artery disease	NA	NA	NA	NA
	COPD	3.577	0.175	26.205	0.2716
	Age>75	NA	NA	NA	NA
	Thyroid disease	3.874	0.763	16.190	0.0719

Endpoint – **DIABETIC COHORT**	Component	OR	CI min.	CI max.	P value
	Hypertension	0.328	0.071	1.506	0.1399
	HFrEF	2.842	0.372	15.153	0.2458
Cardiogenic shock	Coronary artery disease	1.711	0.478	5.861	0.3946
	COPD	2.017	0.273	9.129	0.4141
	Age>75	2.953	0.956	10.108	0.0658
	Thyroid disease	0.656	0.089	2.817	0.6192
	Hypertension	0.398	0.117	1.581	0.1544
	HFrEF	3.055	0.867	11.051	0.0809
Septic shock	Coronary artery disease	0.854	0.366	1.844	0.6994
	COPD	0.308	0.017	1.548	0.2579
	Age>75	0.487	0.227	0.968	0.0497
	Thyroid disease	0.796	0.264	1.949	0.647
	Hypertension	1.283	0.596	3.090	0.5476
pulmonary embolism	HFrEF	1.490	0.681	3.120	0.3015
	Coronary artery disease	0.327	0.050	1.226	0.1512
	COPD	0.864	0.047	4.505	0.8891
	Age>75	1.180	0.480	2.743	0.7065
	Thyroid disease	0.642	0.101	2.283	0.5572
	Hypertension	1.369	0.497	4.836	0.5787
	HFrEF	0.783	0.211	2.293	0.681
deep vein thrombosis	Coronary artery disease	NA	NA	NA	NA
	COPD	NA	NA	NA	NA
	Age>75	1.117	0.051	11.866	0.9284
	Thyroid disease	NA	NA	NA	NA
	Hypertension	NA	NA	NA	NA
	HFrEF	NA	NA	NA	NA
myocardial infarction	Coronary artery disease	0.327	0.050	1.226	0.1512
	COPD	0.864	0.047	4.505	0.8891
	Age>75	1.180	0.480	2.743	0.7065
	Thyroid disease	0.642	0.101	2.283	0.5572
	Hypertension	1.369	0.497	4.836	0.5787
	HFrEF	0.783	0.211	2.293	0.681
myocardial injury	Coronary artery disease	1.269	0.297	4.778	0.7316
	COPD	NA	NA	NA	NA
	Age>75	0.731	0.184	2.494	0.629
	Thyroid disease	0.565	0.030	3.050	0.591
	Hypertension	0.511	0.137	2.445	0.3447
	HFrEF	4.048	1.051	15.611	0.0391
acute heart failure	Coronary artery disease	0.848	0.451	1.561	0.6023
	COPD	0.952	0.307	2.698	0.9276
	Age>75	1.590	0.950	2.656	0.0766
	Thyroid disease	0.482	0.198	1.074	0.0873
	Hypertension	0.948	0.475	1.964	0.8829
	HFrEF	2.121	1.170	3.876	0.0136
stroke/TIA	Coronary artery disease	1.863	0.806	4.249	0.1406
	COPD	0.632	0.090	2.703	0.5822
	Age>75	5.580	2.483	13.820	< 0.0001
	Thyroid disease	0.845	0.256	2.338	0.7621
	Hypertension	1.188	0.405	4.380	0.7709
	HFrEF	4.984	2.182	11.862	0.0002
complete respiratory failure	Coronary artery disease	0.587	0.084	2.499	0.5208
	COPD	1.560	0.083	8.678	0.6781
	Age>75	2.080	0.685	6.317	0.1876
	Thyroid disease	0.524	0.028	2.766	0.5405
	Hypertension	1.370	0.357	9.007	0.687
	HFrEF	0.851	0.171	3.147	0.8227
pneumonia	Coronary artery disease	1.693	0.601	5.016	0.3253
	COPD	0.934	0.225	4.172	0.925

Endpoint – **DIABETIC COHORT**	Component	OR	CI min.	CI max.	P value
	Age>75	1.498	0.611	3.751	0.3793
	Thyroid disease	1.800	0.524	7.057	0.3672
	Hypertension	2.252	0.761	7.041	0.1474
	HFrEF	1.265	0.425	3.895	0.6736
SIRS	Coronary artery disease	1.058	0.503	2.122	0.8777
	COPD	0.897	0.205	2.761	0.8655
	Age>75	1.077	0.586	1.934	0.8079
	Thyroid disease	0.431	0.126	1.121	0.1224
	Hypertension	0.767	0.391	1.604	0.4589
	HFrEF	1.608	0.797	3.156	0.1742
sepsis	Coronary artery disease	0.859	0.100	5.006	0.8738
	COPD	3.526	0.151	37.012	0.3271
	Age>75	1.647	0.332	8.237	0.5309
	Thyroid disease	NA	NA	NA	NA
	Hypertension	0.477	0.090	3.570	0.4055
	HFrEF	2.272	0.393	12.257	0.3355
acute kidney injury	Coronary artery disease	1.959	1.095	3.461	0.0216
	COPD	1.951	0.752	4.674	0.1466
	Age>75	1.415	0.857	2.318	0.1705
	Thyroid disease	0.555	0.230	1.186	0.1548
	Hypertension	1.338	0.702	2.736	0.3974
	HFrEF	1.155	0.634	2.060	0.6296
acute liver dysfunction	Coronary artery disease	1.075	0.298	3.395	0.9055
	COPD	0.771	0.040	4.417	0.8122
	Age>75	1.827	0.667	4.994	0.2337
	Thyroid disease	1.169	0.255	3.841	0.8162
	Hypertension	0.362	0.130	1.098	0.0573
	HFrEF	2.087	0.642	6.427	0.2054
MODS	Coronary artery disease	1.542	0.363	6.719	0.5527
	COPD	NA	NA	NA	NA
	Age>75	0.244	0.035	1.070	0.0903
	Thyroid disease	NA	NA	NA	NA
	Hypertension	0.412	0.076	3.106	0.3232
	HFrEF	21.436	4.221	166.456	0.0007
all bleedings	Coronary artery disease	0.971	0.372	2.305	0.949
	COPD	0.897	0.136	3.359	0.8895
	Age>75	0.963	0.443	1.999	0.9209
	Thyroid disease	1.709	0.652	3.964	0.2379
	Hypertension	0.792	0.354	1.955	0.5883
	HFrEF	1.301	0.526	3.020	0.5516
intracranial bleeding	Coronary artery disease	1.293	0.066	8.421	0.8177
	COPD	NA	NA	NA	NA
	Age>75	1.184	0.161	6.270	0.848
	Thyroid disease	1.592	0.081	10.470	0.6775
	Hypertension	1.423	0.217	27.913	0.7522
	HFrEF	NA	NA	NA	NA
respiratory tract bleeding	Coronary artery disease	1.392	0.331	5.061	0.6275
	COPD	NA	NA	NA	NA
	Age>75	0.263	0.039	1.046	0.0938
	Thyroid disease	2.195	0.473	7.630	0.2511
	Hypertension	0.702	0.192	3.336	0.6151
	HFrEF	2.629	0.674	9.569	0.1468
upper-GI-tract bleeding	Coronary artery disease	NA	NA	NA	NA
	COPD	1.916	0.091	12.720	0.5743
	Age>75	1.183	0.281	4.358	0.8049
	Thyroid disease	2.536	0.521	9.464	0.1932
	Hypertension	0.294	0.082	1.081	0.0556
	HFrEF	2.199	0.426	9.070	0.2967

Endpoint – **DIABETIC COHORT**	Component	OR	CI min.	CI max.	P value
lower-GI-tract-bleeding	Coronary artery disease	12.993	1.152	291.830	0.0422
	COPD	NA	NA	NA	NA
	Age>75	4.394	0.385	98.367	0.2408
	Thyroid disease	2.676	0.111	31.874	0.4513
	Hypertension	0.434	0.036	10.223	0.5196
	HFrEF	NA	NA	NA	NA
urinary tract bleeding	Coronary artery disease	0.436	0.020	3.285	0.4861
	COPD	2.777	0.134	19.292	0.378
	Age>75	1.466	0.274	7.037	0.6307
	Thyroid disease	2.536	0.339	12.702	0.2892
	Hypertension	0.656	0.133	4.781	0.6261
	HFrEF	1.255	0.144	7.291	0.813
pneumonia	Coronary artery disease	0.828	0.505	1.364	0.4553
	COPD	1.361	0.587	3.429	0.4878
	Age>75	1.054	0.704	1.586	0.7983
	Thyroid disease	0.937	0.537	1.659	0.8194
	Hypertension	1.591	0.992	2.547	0.053
	HFrEF	1.277	0.780	2.116	0.3355

Endpoint – **NON-DIABETIC COHORT**	Component	OR	CI min.	CI max.	P value
End of hospitalization - death	Coronary artery disease	2.121	1.300	3.411	0.0022
	COPD	1.033	0.463	2.142	0.9342
	Age>75	2.450	1.742	3.430	< 0.0001
	Thyroid disease	0.439	0.220	0.801	0.0118
	Hypertension	1.976	1.413	2.765	< 0.0001
	HFrEF	2.446	1.544	3.841	0.0001
End of hospitalization - rehabilitation	Coronary artery disease	0.422	0.166	0.921	0.0452
	COPD	2.242	0.975	4.691	0.0416
	Age>75	0.639	0.404	0.983	0.0482
	Thyroid disease	1.079	0.630	1.759	0.7695
	Hypertension	1.259	0.895	1.761	0.1809
	HFrEF	1.193	0.602	2.228	0.5942
End of hospitalization - full recovery	Coronary artery disease	0.658	0.424	1.021	0.062
	COPD	0.594	0.308	1.126	0.1134
	Age>75	0.378	0.290	0.491	< 0.0001
	Thyroid disease	1.665	1.145	2.462	0.0089
	Hypertension	0.744	0.591	0.939	0.0122
	HFrEF	0.573	0.375	0.871	0.0095
All-cause shock	Coronary artery disease	1.022	0.495	1.971	0.951
	COPD	0.802	0.233	2.102	0.6876
	Age>75	0.675	0.405	1.089	0.1186
	Thyroid disease	0.717	0.342	1.347	0.3355
	Hypertension	2.280	1.522	3.422	< 0.0001
	HFrEF	1.949	1.034	3.549	0.0333
Hypovolemic shock	Coronary artery disease	NA	NA	NA	NA
	COPD	1.269	0.069	6.604	0.8205
	Age>75	1.744	0.674	4.235	0.2309
	Thyroid disease	1.170	0.272	3.480	0.8024
	Hypertension	1.433	0.596	3.386	0.4132
	HFrEF	2.330	0.623	7.070	0.1623
Cardiogenic shock	Coronary artery disease	1.572	0.411	5.410	0.4858
	COPD	3.340	0.686	12.100	0.0903
	Age>75	1.697	0.583	4.796	0.3191
	Thyroid disease	0.514	0.028	2.650	0.526
	Hypertension	0.988	0.342	2.883	0.9819
	HFrEF	5.808	1.674	19.682	0.0048
Septic shock	Coronary artery disease	1.392	0.609	2.915	0.4044

Endpoint – **NON-DIABETIC COHORT**	Component	OR	CI min.	CI max.	P value
	COPD	0.563	0.089	1.946	0.4425
	Age>75	0.459	0.233	0.838	0.0161
	Thyroid disease	0.686	0.281	1.431	0.3578
	Hypertension	2.954	1.842	4.770	< 0.0001
pulmonary embolism	HFrEF	1.209	0.524	2.565	0.6372
	Coronary artery disease	0.707	0.272	1.594	0.4353
	COPD	0.883	0.207	2.566	0.8406
	Age>75	0.946	0.544	1.589	0.8388
	Thyroid disease	0.471	0.163	1.074	0.1098
	Hypertension	1.603	1.013	2.523	0.0423
	HFrEF	1.876	0.894	3.711	0.0813
deep vein thrombosis	Coronary artery disease	1.195	0.153	6.043	0.844
	COPD	1.929	0.100	11.401	0.5493
	Age>75	0.682	0.145	2.353	0.5787
	Thyroid disease	1.313	0.204	4.805	0.7218
	Hypertension	0.745	0.227	2.189	0.6052
	HFrEF	2.838	0.488	12.584	0.2007
myocardial infarction	Coronary artery disease	0.652	0.252	1.466	0.335
	COPD	0.850	0.200	2.466	0.7931
	Age>75	0.936	0.544	1.554	0.8027
	Thyroid disease	0.541	0.207	1.164	0.1554
	Hypertension	1.441	0.921	2.237	0.1062
	HFrEF	2.035	0.993	3.956	0.0426
myocardial injury	Coronary artery disease	1.061	0.148	4.792	0.9439
	COPD	NA	NA	NA	NA
	Age>75	2.149	0.612	7.322	0.2196
	Thyroid disease	0.635	0.034	3.344	0.6667
	Hypertension	1.774	0.510	6.541	0.3696
	HFrEF	3.351	0.755	13.261	0.0918
acute heart failure	Coronary artery disease	1.793	1.015	3.123	0.041
	COPD	1.340	0.586	2.929	0.4719
	Age>75	2.324	1.604	3.358	< 0.0001
	Thyroid disease	0.954	0.520	1.672	0.8727
	Hypertension	1.208	0.839	1.735	0.3069
	HFrEF	2.173	1.280	3.663	0.0037
stroke/TIA	Coronary artery disease	1.035	0.443	2.319	0.9355
	COPD	0.458	0.068	1.819	0.3293
	Age>75	2.389	1.158	4.989	0.0189
	Thyroid disease	0.305	0.047	1.121	0.1235
	Hypertension	0.995	0.454	2.197	0.9893
	HFrEF	29.622	13.006	71.493	< 0.0001
complete respiratory failure	Coronary artery disease	0.790	0.119	3.029	0.765
	COPD	NA	0.000	NA	NA
	Age>75	1.690	0.671	4.004	0.2447
	Thyroid disease	0.664	0.105	2.295	0.5835
	Hypertension	2.247	0.963	5.332	0.061
	HFrEF	1.050	0.227	3.542	0.9423
pneumonia	Coronary artery disease	1.937	0.735	5.324	0.1855
	COPD	0.886	0.204	3.989	0.8699
	Age>75	1.443	0.744	2.815	0.2786
	Thyroid disease	0.532	0.168	1.560	0.2589
	Hypertension	0.580	0.297	1.115	0.1054
	HFrEF	2.454	0.994	6.387	0.0561
SIRS	Coronary artery disease	0.812	0.377	1.608	0.5718
	COPD	1.684	0.664	3.721	0.2293
	Age>75	0.974	0.623	1.488	0.9058
	Thyroid disease	0.673	0.336	1.220	0.2237

Endpoint – **NON-DIABETIC COHORT**	Component	OR	CI min.	CI max.	P value
	Hypertension	0.811	0.555	1.172	0.2712
	HFrEF	1.582	0.826	2.896	0.1499
sepsis	Coronary artery disease	0.265	0.013	1.712	0.2399
	COPD	2.201	0.107	15.897	0.4974
	Age>75	1.049	0.261	3.773	0.9434
	Thyroid disease	1.173	0.174	4.707	0.8417
	Hypertension	1.710	0.465	6.418	0.4125
	HFrEF	6.983	1.623	29.784	0.0077
acute kidney injury	Coronary artery disease	1.232	0.667	2.179	0.488
	COPD	0.709	0.236	1.720	0.488
	Age>75	1.176	0.774	1.760	0.4381
	Thyroid disease	0.430	0.189	0.851	0.0262
	Hypertension	2.536	1.740	3.711	< 0.0001
	HFrEF	1.839	1.056	3.127	0.0274
acute liver dysfunction	Coronary artery disease	1.069	0.371	2.648	0.8924
	COPD	NA	0.000	NA	NA
	Age>75	1.476	0.755	2.800	0.2415
	Thyroid disease	0.659	0.195	1.677	0.4362
	Hypertension	2.393	1.271	4.594	0.0074
	HFrEF	1.770	0.715	4.029	0.1916
MODS	Coronary artery disease	0.256	0.013	1.458	0.2094
	COPD	2.439	0.370	9.279	0.2544
	Age>75	0.612	0.192	1.630	0.3599
	Thyroid disease	0.710	0.113	2.450	0.6458
	Hypertension	1.912	0.825	4.391	0.125
	HFrEF	2.406	0.619	7.502	0.1588
all bleedings	Coronary artery disease	1.178	0.488	2.583	0.6972
	COPD	0.697	0.110	2.427	0.6306
	Age>75	0.988	0.541	1.733	0.9669
	Thyroid disease	2.035	1.063	3.653	0.023
	Hypertension	1.297	0.779	2.142	0.3113
	HFrEF	2.085	0.956	4.309	0.0545
intracranial bleeding	Coronary artery disease	NA	NA	NA	NA
	COPD	NA	NA	NA	NA
	Age>75	1.474	0.416	4.627	0.52
	Thyroid disease	NA	NA	NA	NA
	Hypertension	2.794	0.915	8.892	0.0709
	HFrEF	1.696	0.245	7.240	0.5212
respiratory tract bleeding	Coronary artery disease	1.887	0.349	7.650	0.4123
	COPD	1.432	0.075	8.240	0.7417
	Age>75	0.136	0.007	0.700	0.0571
	Thyroid disease	0.920	0.144	3.279	0.9128
	Hypertension	1.340	0.494	3.425	0.5488
	HFrEF	1.982	0.361	8.267	0.3843
upper-GI-tract bleeding	Coronary artery disease	1.715	0.424	5.655	0.4064
	COPD	1.074	0.057	5.814	0.947
	Age>75	2.084	0.783	5.373	0.1306
	Thyroid disease	2.700	0.857	7.190	0.0616
	Hypertension	1.728	0.656	4.738	0.2724
	HFrEF	1.735	0.469	5.637	0.3787
lower-GI-tract-bleeding	Coronary artery disease	NA	NA	NA	NA
	COPD	NA	NA	NA	NA
	Age>75	1.150	0.057	8.042	0.9031
	Thyroid disease	10.294	1.836	57.601	0.0054
	Hypertension	0.771	0.098	4.348	0.7781
	HFrEF	NA	NA	NA	NA
urinary tract bleeding	Coronary artery disease	1.546	0.200	8.040	0.6313

Endpoint – **NON-DIABETIC COHORT**	Component	OR	CI min.	CI max.	P value
	COPD	NA	NA	NA	NA
	Age>75	0.898	0.178	3.567	0.885
	Thyroid disease	0.795	0.043	4.309	0.8293
	Hypertension	1.593	0.411	6.181	0.4921
	HFrEF	3.527	0.600	17.218	0.1335
pneumonia	Coronary artery disease	0.962	0.618	1.509	0.8653
	COPD	1.902	0.964	4.034	0.0756
	Age>75	1.281	0.980	1.677	0.0706
	Thyroid disease	1.251	0.887	1.773	0.2042
	Hypertension	2.432	1.947	3.045	< 0.0001
	HFrEF	1.376	0.892	2.151	0.1538
